# Sensing of DNA double-strand breaks by the NHEJ system stabilizes RORγt transcriptional activity and shapes Th17 pathogenicity in autoimmunity

**DOI:** 10.1038/s41422-025-01204-6

**Published:** 2026-01-07

**Authors:** Guan-Yu Chen, Wen-Jie Zhu, Zhuang Li, Yun-Wei Hu, Xiao-Shuang Luo, Zhi-Qing Mai, Yuan Pan, Yu-Xun Shi, Zuo-Yi Li, Jun Huang, Pei-Dong Yuan, Zhi-Qiang Xiao, Qian Chen, Yan-Yan Xie, Hai-Xiang Huang, Yu-Xi Chen, Yao Lu, Min-Zhen Wang, Yi-Wen Xia, Xiao-Qing Chen, Dong-Ming Kuang, Dan Liang

**Affiliations:** 1 State Key Laboratory of Ophthalmology, Zhongshan Ophthalmic Center, Sun Yat-sen University, Guangdong Provincial Key Laboratory of Ophthalmology and Visual Science, Guangzhou, Guangdong China; 2 Department of Ophthalmology, The Second Affiliated Hospital of Nanchang University, Nanchang, Jiangxi China; 3 Department of Ophthalmology, The Seventh Affiliated Hospital, Sun Yat-sen University, Shenzhen, Guangdong China; 4 Department of Ophthalmology, The Third Affiliated Hospital, Sun Yat-sen University, Guangzhou, Guangdong China; 5 Department of Ophthalmology, Shanghai General Hospital, Shanghai Jiao Tong University School of Medicine, Shanghai, China; 6 Guangdong Province Key Laboratory of Pharmaceutical Functional Genes, MOE Key Laboratory of Gene Function and Regulation, School of Life Sciences, Sun Yat-sen University, Guangzhou, Guangdong China

**Keywords:** Autoimmunity, Mechanisms of disease

## Abstract

Robust mitochondrial ROS production induces extensive double-strand breaks (DSBs) in telomeric DNA of effector T cells, where the DNA repair machinery is rapidly hyper-evoked to sense and ligate DSBs during the respiratory burst. However, whether effector T cells can exploit the DNA repair system to simultaneously potentiate their functional activation remains largely unknown, especially in the context of autoimmunity. Here, we demonstrate that non-homologous end joining (NHEJ), a predominant mechanism of DNA repair, is highly activated in pathogenic T helper 17 (pTh17) cells and exerts a previously unrecognized effect on shaping the pathogenic nature of pTh17s to trigger autoimmunity. Mechanistically, the perception of DSBs by KU proteins facilitates auto-phosphorylation of DNA-dependent protein kinase catalytic subunit (DNA-PKcs), which stabilizes RORγt to bind to the promoters of effector-gene loci, thus initiating the pTh17 effector program to induce autoimmunity. Using mass spectrometry and transcriptome analyses, we identified IER2 as a novel NHEJ factor that potentiates DNA-PKcs kinase activity in response to IL-23R stimulation, which is necessary for shaping Th17 pathogenicity. Therefore, targeting the immuno-pattern of the NHEJ system shows potential for the treatment of autoimmune diseases.

## Introduction

T helper 17 (Th17) cells, which are governed by the master transcription factor (TF) RORγt, play a vital role in mediating antimicrobial responses to protect organisms from infection.^1,2^ Although Th17 cells are beneficial partners in the physiological immune environment, sustained Th17 activation can be pathogenic, directly inducing autoimmunity and contributing to chronic inflammation.^3^ However, the mechanism underlying this pathogenic conversion is complex, impeding the effective treatment of autoimmune diseases.^4,5^ Therefore, understanding the intrinsic pathogenic program of Th17 cells is essential, as it may guide the development of more effective therapeutic strategies aimed at alleviating autoimmunity and restoring immune tolerance.

An increase in DNA double-strand breaks (DSBs) in T cells is a classical hallmark of autoimmune diseases.^6,7^ Upon stimulation by a self-antigen, accelerated mitochondrial metabolism leads to a massive release of reactive oxygen species (ROS), which trigger DSBs in auto-reactive T cells.^8,9^ Although the accumulation of DSBs is usually lethal to the cell, certain cellular programs triggered by DSB perception can endow T cells with a pro-inflammatory phenotype, which is strongly associated with disease development and chronicity.^10,11^ These findings suggest that DSB-derived signals may be necessary for the differentiation of pathogenic effector T cells. In fact, a higher Th17 frequency has been observed in radiation-treated mice, indicating that sensing of DSBs might be a prerequisite for Th17 differentiation.^12^ In addition, Th17 cells are more active in response to DSBs and exhibit less susceptibility to DSB-induced cell death than other Th subsets.^13,14^ These findings imply that the occurrence of DSBs might be critical for the Th17-mediated pathogenic T cell response. Therefore, targeting DSB perception and its subsequent responses could represent a novel approach for the treatment of autoimmune diseases.

In general, DSB ends marked by γH2AX phosphorylation will recruit several factors to sense DSBs and initiate the DSB repair process. To date, a number of independent DSB repair pathways have been identified, including homologous recombination, non-homologous end joining (NHEJ), alternative end joining, and single-strand annealing.^15^ NHEJ is particularly noteworthy, as it operates throughout the cell cycle and resolves 25%–50% of genomic DSBs in mammalian cells.^16^ The core NHEJ enzyme, DNA-dependent protein kinase catalytic subunit (DNA-PKcs), is related to the expression of GM-CSF, IL-21, CCR4, and IL-1 receptor,^17^ and these molecules are known to be associated with Th17 effector function.^18,19^ Hence, essential questions include whether NHEJ might bias differentiation towards the Th17 program to elicit its pathogenesis in autoimmunity and, if so, how NHEJ influences Th17 effector function.

In this study, we reveal that sensing of DSBs by the NHEJ system controls the expression of Th17 pathogenic cytokines through stabilization of RORγt binding to effector-gene loci. DNA-PKcs, which receives DSB signals from KUs, shapes the pathogenic Th17 (pTh17) effector program and triggers autoimmunity in mice, whereas DNA ligase IV-dependent DSB ligation is largely dispensable. Mechanistically, we found that DNA-PKcs not only initiates end joining for DSB clearance but also acts as a novel immuno-booster that stabilizes RORγt to bind to effector-gene loci. Moreover, we identified a novel NHEJ factor, immediate early response 2 (IER2), that responds to IL-23R signaling and enhances the kinase activity of DNA-PKcs to promote the two processes above. These findings suggest that the perception of DSBs by the NHEJ system dominates the effector function of pathogenic Th17 cells, thereby triggering inflammation in autoimmunity.

## Results

### DSB sensing controls the effector function of Th17 cells

**Fig. 1:**
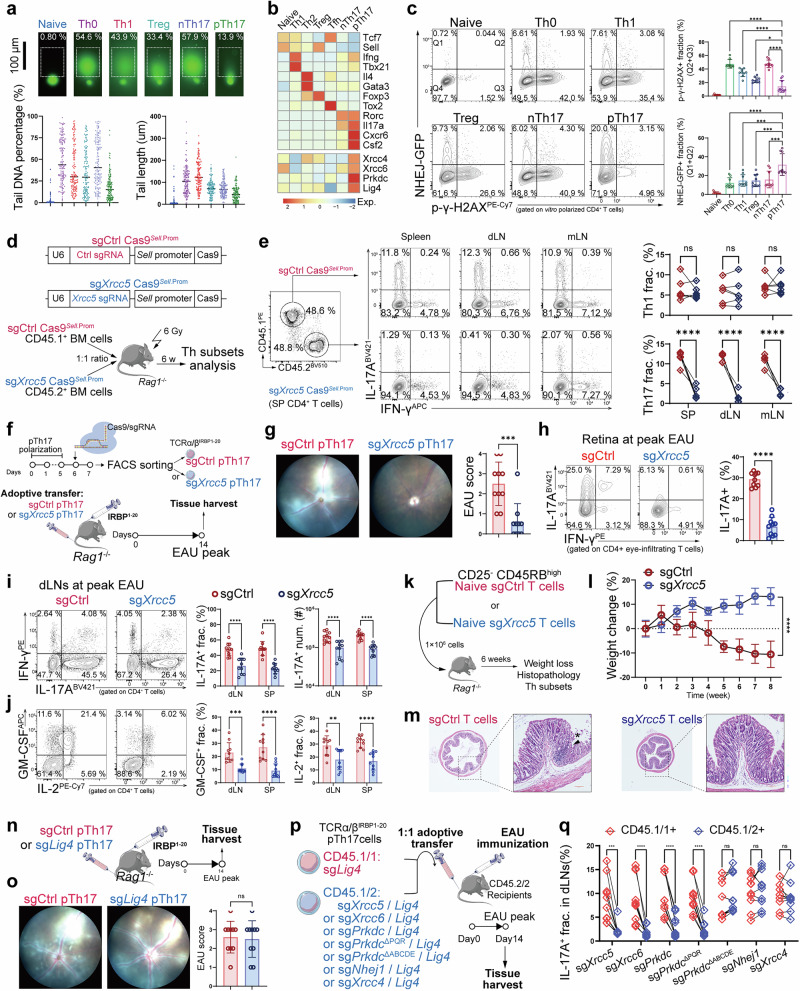
DSB sensing controls the effector function of Th17 cells in autoimmunity. **a** Representative images and statistical graphs of the comet assay for each CD4^+^ T subset, showing the extent of DNA damage after 5 days of polarization of human naïve T cells (scale bar: 100 μm). The experiment was repeated three times, and 50 individual cells were analyzed each time (*n* = 150). **b** Heatmap derived from murine scRNA-seq data from EAU mice, showing the expression of NHEJ genes in each CD4^+^ T subset. **c** Flow cytometry (FC) analysis of an NHEJ assay, showing the NHEJ activity and p-γ-H2AX^+^ fraction of each CD4^+^ T subset after a 5-day polarization of human naïve T cells (*n* = 9). **d** Experimental scheme showing the generation of a mixed BM chimera using CD45.1/1^+^ BM and CD45.2/2^+^ BM transfected with sgCtrl-Cas9^*Sell*.Promoter^ and sg*Xrcc5*-Cas9^*Sell*.Promoter^ lentiviral vectors, respectively. Six weeks after reconstitution, the proportion of each Th subset in lymph organs was analyzed. Data were combined from two independent experiments with *n* = 6. **e** FC analysis gated on CD4^+^ T cells, showing the IFN-γ^+^ and IL-17A^+^ fraction of chimera mice reconstituted with mixed BM (*n* = 6). **f** Experimental scheme to examine the role of the NHEJ pathway in Th17 effector function by adoptively transferring sgCtrl or sg*Xrcc5* pTh17 to EAU *Rag1*^−/−^ mice. At day 14 (EAU peak), retina, dLNs, and SPs were harvested for FC assays. Data were combined from three independent experiments with *n* = 10. **g** Fundus images showing the ocular fundus of EAU *Rag1*^−/−^ mice transferred with sgCtrl or sg*Xrcc5* pTh17, with the related statistical graph (*n* = 10). **h** FC analysis of the retina-infiltrating Th17 fraction of mice that received sgCtrl or sg*Xrcc5* pTh17 cells (*n* = 10). **i** FC analysis gated on CD4^+^ T cells showing the fraction and number of IL-17A^+^ cells in lymph organs of EAU *Rag1*^−/−^ mice transferred with sgCtrl or sg*Xrcc5* pTh17 (*n* = 10). **j** FC analysis gated on CD4^+^ T cells showing the fraction of GM-CSF^+^ and IL-2^+^ cells in lymph organs of EAU *Rag1*^−/−^ mice transferred with sgCtrl or sg*Xrcc5* pTh17 (*n* = 10). **k** Experimental scheme showing adoptive transfer of WT and *Xrcc5*-deficient CD25^−^ CD45RB^high^ naive CD4^+^ T cells into *Rag1*^−/−^ mice. Data were combined from two independent experiments with *n* = 6. **l** Weight loss of *Rag1*^−/−^ mice after transfer of sgCtrl or sg*Xrcc5* naive CD4^+^ T cells (*n* = 6). **m** Representative H&E images of colon segments (*n* = 6). **n** Experimental scheme to examine the role of DSB ligation in Th17 effector function by adoptively transferring sgCtrl or sg*Lig4* pTh17 to EAU *Rag1*^−/−^ mice. At day 14 (EAU peak), retina, dLNs, and SPs were harvested for FC assays. Data were combined from three independent experiments with *n* = 10. **o** Fundoscopic images showing the ocular fundus of EAU *Rag1*^−/−^ mice transferred with sgCtrl or sg*Lig4* pTh17, with the related statistical graph (*n* = 10). **p** Experimental scheme showing adoptive transfer of CD45.1/1^+^ sg*Lig4* polarized-pTh17 cells and CD45.1/2^+^ polarized-pTh17 cells with double knockout of *Lig4* and individual NHEJ factors to CD45.2/2^+^ EAU recipients. Data were combined from three independent experiments with *n* = 10. **q** Statistical chart showing the frequency of IL-17A-secreting cells gated in the transferred CD45.1/1^+^ or CD45.1/2^+^ cells in **p** (*n* = 10). Significance was assessed by paired or unpaired Student’s *t*-test or one-way analysis of variance, followed by Tukey’s test or two-way analysis of variance, followed by Bonferroni’s test. Error bars represent mean ± SD. Comet assay data (non-parametric) are shown as individual values with median, and multiple comparisons were performed by one-way analysis of variance followed by Dunn’s test. ^*^P < 0.05, ^**^P < 0.01, ^***^P < 0.001, ^****^P < 0.0001. See also Supplementary information, Figs. S1–S3.

Activated T cells experience large numbers of DSBs owing to the release of ROS.^20^ We first examined the heterogeneity in DSB susceptibility among Th subsets using an in vitro neutral comet assay. We found that one type of polarized Th17 cell exhibited a shorter tail length and lower tail DNA percentage than the other Th subsets (Fig. 1a). Only Th17 induced by IL-6, IL-1β, and IL-23, also known as pTh17, showed resistance to DSB formation, in contrast to its counterpart, non-pathogenic Th17 (nTh17) induced by IL-6 and TGF-β. Single-cell RNA sequencing (scRNA-seq) of murine memory CD4^+^ T cells revealed higher expression of NHEJ component genes (e.g., *Xrcc4*, *Xrcc6*, *Prkdc*, and *Lig4*) in local pTh17 cells (Fig. 1b; Supplementary information, Fig. S1a, b). We observed the same DSB levels in each effector Th subset when we ablated the intrinsic DSB-ligating molecules Ligase IV for NHEJ and RAD51 for homologous recombination, indicating that the generation of DSBs was the same (Supplementary information, Fig. S1c). pTh17 also exhibited the strongest NHEJ activity and resistance to DSB accumulation in an NHEJ assay (Fig. 1c). Immunofluorescence showed that DSBs were predominantly localized to telomeres in human *LIG4*^KO^ pTh17 cells, suggesting that the NHEJ system was strongly activated by telomeric DSBs (Supplementary information, Fig. S1d). We confirmed that pTh17 cells were resistant to DSB accumulation brought on by autoimmunity in vivo, because transferred pTh17 cells exhibited the lowest DSB level at the peak of experimental autoimmune uveitis (EAU) (Supplementary information, Figs. S1e, f and S11c). These data reveal that the elevated NHEJ activity in pTh17 cells confers greater resistance to DSB accumulation.

We next asked whether NHEJ could maintain the persistence and pathogenicity of Th17 cells. The *Xrcc5* gene encodes the KU80 protein, an initiating factor of NHEJ responsible for sensing DSBs. First, we generated mixed bone marrow (BM) chimeric mice by reconstituting CD45.1/1^+^ wild-type (WT) and CD45.2/2^+^ sg*Xrcc5* BM cells with Cas9 working in a mature T lineage (Fig. 1d). We found that IL-17A^+^ cells were mainly found among CD45.1/1^+^ WT T cells (Fig. 1e), whereas thymocyte development and the proportion of Treg, Th1, and Th2 cells were almost unbiased (Supplementary information, Fig. S1g, h), suggesting that the Th17 response might require the NHEJ system. Next, an in vitro assay showed that *XRCC5* knockout restricted the production of IL-17A, IL-2, and GM-CSF, as well as the expression of activation markers after pTh17 differentiation, but neither were impaired under Th1 or nTh17 polarizing conditions (Supplementary information, Fig. S1i–k). In vivo, *Rag1*^−/−^ mice that received *Xrcc5*^KO^ pTh17 showed limited EAU immunopathology (Fig. 1f, g). Both the proportion and number of intraocular and interlymphoid IL-17A-secreting cells were reduced (Fig. 1h, i), as was the secretion of pro-inflammatory cytokines, including IL-2 and GM-CSF (Fig. 1j; Supplementary information, Fig. S1l). Pharmacological inhibition of KU–DSB complex formation using STL127705^21^ also attenuated EAU pathology and suppressed Th17 response (Supplementary information, Fig. S1m–r). Moreover, in an autoimmune colitis model, mice that received *Xrcc5*^KO^ T cells showed attenuation of weight loss, colon bleeding, and epithelial inflammation (Fig. 1k–m; Supplementary information, Fig. S1s, t). In line with these findings, transplanted *Xrcc5*^KO^ T cells showed a lower proportion of both Th17 and Th1, but especially Th17 (Supplementary information, Fig. S1u). Collectively, these data demonstrate that NHEJ is specifically required for the pathogenicity and effector function of Th17 cells in autoimmunity.

The final step of the NHEJ pathway, rejoining the two ends of the damaged DNA strands, is performed by DNA ligase IV (encoded by *LIG4*).^22^ However, we excluded the possibility that NHEJ-mediated DSB ligation sustained Th17 pathogenicity because we observed that neither transfer of *Lig4*^KO^ pTh17 nor pharmacological inhibition of DNA Ligase IV activity prevented autoimmunity (Fig. 1n, o; Supplementary information, Fig. S2a–g). In vitro, *LIG4* knockout did not impair pTh17 differentiation and activation (Supplementary information, Fig. S2h, i). Although either *XRCC5* or *LIG4* knockout could inhibit cell proliferation, the secretion of IL-17A was reduced only in the case of *XRCC5* knockout (Supplementary information, Fig. S3a). To further confirm that pTh17 function relied on KU-dependent DSB sensing rather than DNA ligase IV-mediated DSB ligation, we knocked out each NHEJ factor in *LIG4*-KO T cells whose DSB capacity was saturated. Interestingly, we noticed that the ablation of DSB-sensing factors, including KU70, KU80, and DNA-PKcs, still reduced *LIG4*-KO pTh17 differentiation, suggesting that pTh17 differentiation was dependent on DSB sensing but not on DNA ligase IV-mediated DSB ligation (Supplementary information, Fig. S3b–d). Similarly, in vivo-transplanted *Lig4*-deficient pTh17 with subsequent knockout of *Xrcc5*, *Xrcc6*, and *Prkdc* also exhibited impaired IL-17A secretion and weakened retinal infiltration, but subsequent knockout of *Nhej1* or *Xrcc4* did not affect this process, supporting the conclusions derived from the in vitro experiments (Fig. 1p, q; Supplementary information, Fig. S3e–g). Thus, these data suggest that NHEJ-dependent DSB perception, not end ligation, determines the effector function of Th17 cells.

### DNA-PKcs stabilizes the binding of RORγt to effector-gene loci to maintain Th17 pathogenicity

**Fig. 2:**
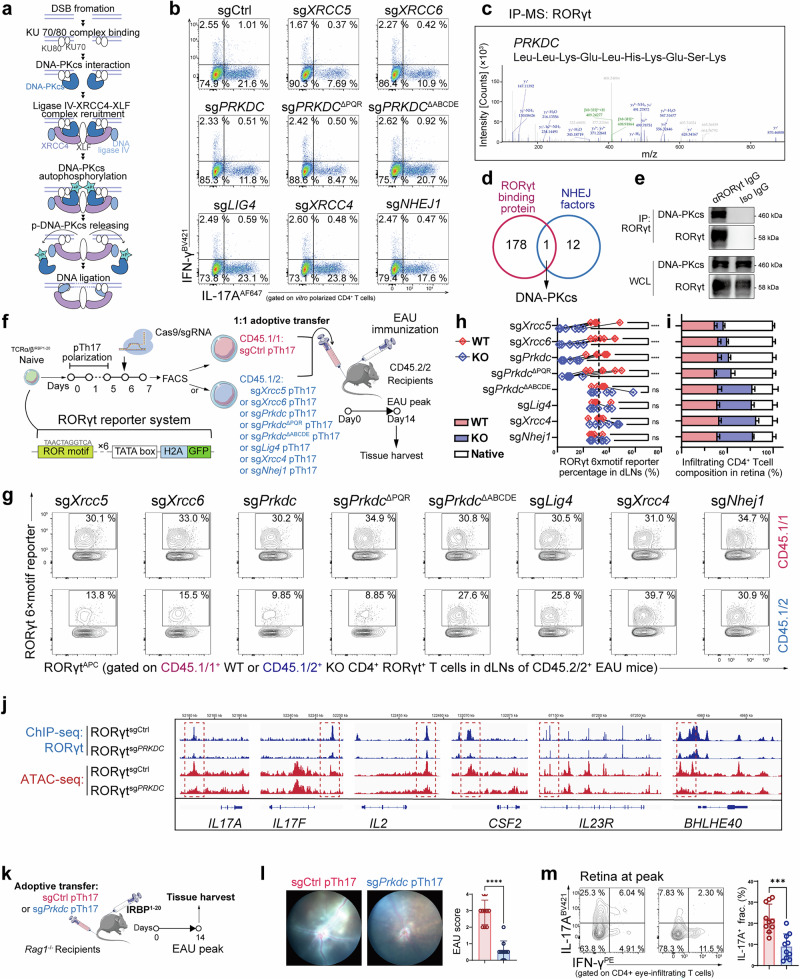
DNA-PKcs stabilizes RORγt transcriptional activity to sustain Th17 pathogenicity. **a** Diagram showing each step of the NHEJ process. **b** FC analysis showing the effect of knockout of each NHEJ factor on human pTh17 differentiation (*n* = 3). **c** IP-MS analysis of the potential protein interacting with RORγt in polarized human pTh17 cells. **d** Venn diagram for IP-MS showing the NHEJ factors that RORγt interacted with in polarized human pTh17 cells. **e** Immunoblot for co-IP assay showing the interaction between DNA-PKcs and RORγt in polarized human pTh17 cells (*n* = 3). **f** Experimental scheme showing how polarized pTh17 cells carrying the RORγt 6× motif reporter system with sgCtrl (CD45.1/1^+^) or with ablation of each NHEJ factor (CD45.1/2^+^) were adoptively transferred to CD45.2/2^+^ EAU recipients. At day 14 (EAU peak), retinas, dLNs, and SPs were harvested for FC assays. Data were combined from two independent experiments with *n* = 6. **g** FC analysis showing the effect of ablation of each NHEJ factor on the expression of the RORγt 6× motif reporter in transferred cells (*n* = 6). **h** Statistical graph for **g**. **i** Composition of CD45.1/1^+^ sgCtrl pTh17 and CD45.1/2^+^ pTh17 with knockout of each NHEJ factor in retinas of CD45.2/2^+^ EAU mice (*n* = 6). **j** ChIP-seq tracks of RORγt within effector genes of human polarized pTh17 cells integrated with ATAC-seq. **k** Experimental scheme to examine the role of DNA-PKcs in Th17 effector function by adoptively transferring sgCtrl or sg*Prkdc* pTh17 to EAU *Rag1*^−/−^ mice. Data were combined from three independent experiments with *n* = 10. **l** Fundoscopic images showing the ocular fundus of EAU *Rag1*^−/−^ mice transferred with sgCtrl or sg*Prkdc* pTh17, with the related statistical graph (*n* = 10). **m** FC analysis of the retina-infiltrating Th17 fraction of EAU *Rag1*^−/−^ mice that received sgCtrl with sg*Prkdc* pTh17 cells (*n* = 10). Significance was assessed by unpaired Student’s *t*-test or one-way analysis of variance followed by Tukey’s test or two-way analysis of variance, followed by Bonferroni’s test. Error bars represent mean ± SD. ^*^P < 0.05, ^**^P < 0.01, ^***^P < 0.001, ^****^P < 0.0001. See also Supplementary information, Fig. S4.

We next wondered which factor in the NHEJ pathway directly affected Th17 pathogenicity. In brief, after DNA damage, the KU70/80 complex binds to DSB ends and recruits DNA-PKcs to activate its kinase activity. Then, the complex recruits DNA ligase IV, XRCC4, and XLF. At some point, DNA-PKcs is auto-phosphorylated to leave the KU70/80 complex and provide access for end ligation. Finally, DNA ligase IV ligates the two ends of the DSB sites to complete the DNA repair^23^ (Fig. 2a). We therefore knocked out each NHEJ factor and found that DNA-PKcs was the last step in the NHEJ pathway that affected pTh17 differentiation (Fig. 2b).

In another assay, we used a 6× motif reporter system to measure the activity of Th17-associated TFs. We found that ablation of NHEJ reduced the ability of RORγt to drive expression of the motif reporter, but it did not affect any other TFs (Supplementary information, Fig. S4a). A screening strategy also revealed that DNA-PKcs was the last step in NHEJ that affected RORγt reporter expression (Supplementary information, Fig. S4b). Furthermore, both an immunoprecipitation-mass spectrometry (IP-MS) assay and co-immunoprecipitation (co-IP) showed that RORγt bound to DNA-PKcs in pTh17 cells (Fig. 2c–e). These findings suggested that DNA-PKcs might control the pTh17 response by affecting RORγt. In vivo, we adoptively transferred pTh17 cells carrying the RORγt 6× motif reporter system into EAU mice (Fig. 2f). RORγt in *Prkdc*-deficient pTh17 cells drove only weak expression of the motif reporter, and these cells showed limited retinal infiltration (Fig. 2g–i). Chromatin immunoprecipitation sequencing (ChIP-seq) and assay for transposase-accessible chromatin using sequencing (ATAC-seq) revealed that knockout of DNA-PKcs impaired RORγt binding to the promoters of inflammatory genes, including *IL17A*, *IL2*, *CSF2*, *BHLHE40*, and *IL23R*, and reduced chromatin accessibility at these regions (Fig. 2j). Together, these results showed that DNA-PKcs was required for binding of RORγt to effector-gene loci and sustained chromatin accessibility at these genes to support the Th17 transcriptional program.

As expected, *Rag1*^−/−^ mice that received *Prkdc*^KO^ pTh17 were not susceptible to EAU induction, and secretion of IL-17A, IL-2, and GM-CSF was also restricted, with reduced retinal infiltration (Fig. 2k–m; Supplementary information, Fig. S4c, d). Pharmacological inhibition of DNA-PKcs kinase activity (NU7441) in vivo also attenuated EAU inflammation (Supplementary information, Fig. S4e–g). In vitro, knockout of *PRKDC* also repressed the differentiation of pTh17 and inhibited the production of IL-2 and GM-CSF (Supplementary information, Fig. S4h). In addition, knockout of *PRKDC* reduced pTh17 cell proliferation and increased sensitivity to apoptosis (Supplementary information, Fig. S4i). Thus, these data reveal that DNA-PKcs in the NHEJ pathway receives a KU-derived DSB signal to support the transcriptional activity of RORγt and maintain the pathogenicity of Th17 in autoimmunity.

### Auto-phosphorylation at the PQR cluster is required for the interaction of DNA-PKcs with RORγt

**Fig. 3:**
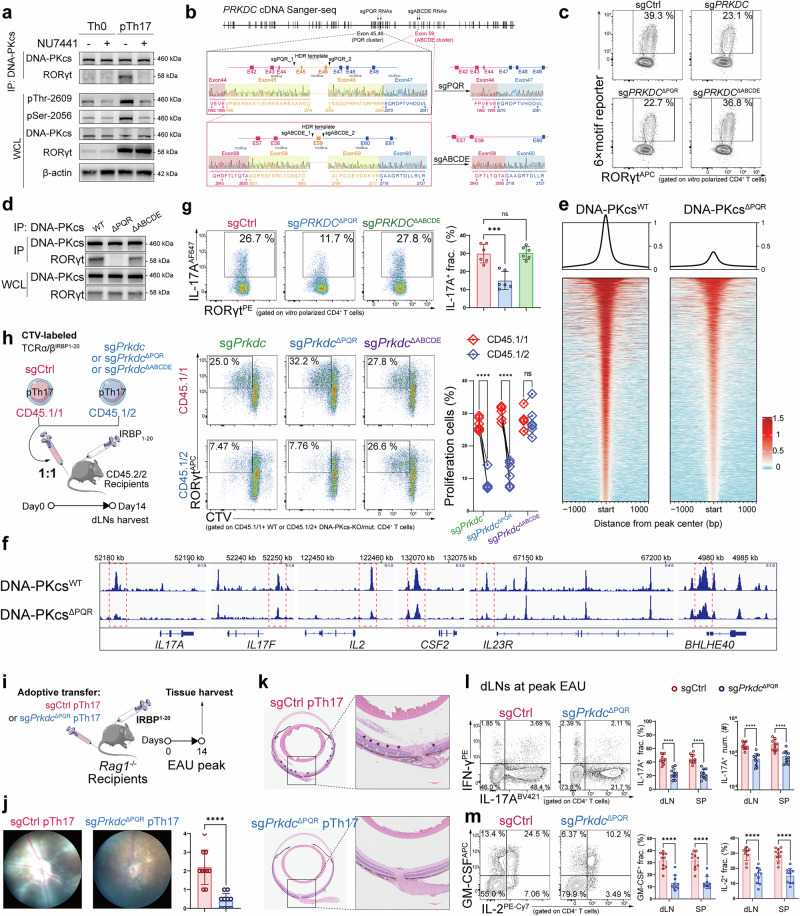
Auto-phosphorylation at the PQR cluster maintains the binding of RORγt to effector-gene loci. **a** Immunoblot for co-IP assay showing the interaction between DNA-PKcs and RORγt in polarized human Th0 or pTh17 cells treated with or without 100 nM NU7441 (*n* = 3). **b** Sanger sequencing of *PRKDC* mRNA, showing mutations that deleted the PQR cluster or the ABCDE cluster in polarized human Th17 cells. The yellow region of the sequence (PQR/E45-46 or ABCDE/E59) was deleted, and the adjacent exons (left-red, right-blue) were connected directly in the presence of HDR donors, enabling us to obtain DNA-PKcs that lacked the PQR cluster or the ABCDE cluster. **c** FC analysis showing the expression of the RORγt 6× motif reporter in pTh17 induced from sgCtrl, sg*PRKDC*, sg*PRKDC*^ΔPQR^, and *sgPRKDC*^ΔABCDE^ human naïve T cells (*n* = 4). **d** Immunoblot for co-IP assay showing the interaction between DNA-PKcs and RORγt in human polarized pTh17 cells induced from sgCtrl, sg*PRKDC*^ΔPQR^, and *sgPRKDC*^ΔABCDE^ human naïve T cells (*n* = 4). **e** Genome-wide ChIP-seq analysis of RORγt in sgCtrl or sg*PRKDC*^ΔPQR^ human polarized pTh17 cells. **f** ChIP-seq tracks of RORγt within pTh17 effector-program genes. **g** FC analysis showing the effects of deleting the PQR cluster or the ABCDE cluster on human pTh17 differentiation (*n* = 6). **h** Experimental scheme and FC analysis showing in vivo murine Th17 proliferation after 1:1 adoptive transfer of CD45.1/1^+^ sgCtrl pTh17 cells along with CD45.1/2^+^ sg*Prkdc*, sg*Prkdc*^ΔPQR^, or sg*Prkdc*^ΔABCDE^ pTh17 into CD45.2/2^+^ EAU recipients. Data were combined from two independent experiments with *n* = 6. **i** Experimental scheme to examine the role of auto-phosphorylation at the PQR cluster in Th17 effector function by adoptive transfer of sgCtrl or sg*Prkdc*^ΔPQR^ pTh17 to EAU *Rag1*^−/−^ mice. **j** Fundoscopic images showing retinal pathological alterations of EAU *Rag1*^−/−^ mice transferred with sgCtrl or sg*Prkdc*^ΔPQR^ pTh17 (*n* = 10). **k** Representative H&E images of the retinal layers of eyeballs from EAU *Rag1*^−/−^ mice transferred with sgCtrl or sg*Prkdc*^ΔPQR^ pTh17 (*n* = 10). **l** FC analysis gated on CD4^+^ T cells showing the fraction and number of IL-17A^+^ cells in lymph organs of EAU *Rag1*^−/−^ mice transferred with sgCtrl or sg*Prkdc*^ΔPQR^ pTh17 (*n* = 10). **m** FC analysis gated on CD4^+^ T cells showing the fraction of GM-CSF^+^ and IL-2^+^ cells in lymph organs of EAU *Rag1*^−/−^ mice transferred with sgCtrl or sg*Prkdc*^ΔPQR^ pTh17 (*n* = 10). Significance was assessed by unpaired Student’s *t*-test or one-way analysis of variance, followed by Tukey’s test or two-way analysis of variance, followed by Bonferroni’s test. Error bars represent mean ± SD. ^*^P < 0.05, ^**^P < 0.01, ^***^P < 0.001, ^****^P < 0.0001. See also Supplementary information, Fig. S5.

Next, we asked whether only the activated form of DNA-PKcs could drive the Th17 effector program, as its inactivated form was abundantly expressed in various cell types. Two major post-translational modification sites of DNA-PKcs have previously been identified: autophosphorylation at the ABCDE cluster and the PQR cluster, represented by Thr2609 and Ser2056, respectively. Upon activation, DNA-PKcs is auto-phosphorylated at both clusters, which are required for further conformational change within NHEJ complexes.^24,25^ We found that in vitro polarized pTh17 cells displayed increased auto-phosphorylation levels at both sites. However, application of a DNA-PKcs kinase blocker inhibited auto-phosphorylation and reversed the interaction with RORγt (Fig. 3a). To confirm that auto-phosphorylation was required for the binding of DNA-PKcs to RORγt, we constructed DNA-PKcs mutants that lacked the PQR or ABCDE cluster in pTh17 cells by introducing dual sgRNAs flanking the encoding exons (Supplementary information, Fig. S5a), an approach based on a previous strategy.^26^ Sanger sequencing confirmed the expected junction of distal exonic segments in the amplified *PRKDC* cDNA (Fig. 3b). Both *PRKDC*^ΔPQR^ and *PRKDC*^ΔABCDE^ mutants had missing sequences at the corresponding residues, but total expression of DNA-PKcs was unchanged (Supplementary information, Fig. S5b).

We next illustrated that phosphorylation at the PQR site, the modification notably elevated in pTh17 cells (Supplementary information, Fig. S5c), is critically required for the functional interaction between DNA-PKcs and RORγt. Deletion of the PQR cluster, rather than the ABCDE cluster, inhibited the interaction of DNA-PKcs with RORγt, as well as the expression of the RORγt 6× motif reporter (Fig. 3c, d). Furthermore, ChIP-seq revealed that binding of RORγt to effector-gene loci was reduced in pTh17 with the PQR deletion (Fig. 3e, f), suggesting that the PQR cluster is required for the conformational structure that allows DNA-PKcs to interact with RORγt.

PQR deletion also reduced pTh17 differentiation, cytokine release, and proliferation (Fig. 3g, h; Supplementary information, Fig. S5d, e). We adoptively transferred PQR-deleted (*Prkdc*^ΔPQR^) pTh17 cells to *Rag1*^−/−^ mice to observe their pathogenicity (Fig. 3i) and found that *Prkdc*^ΔPQR^ pTh17 cells triggered less retinal inflammation (Fig. 3j, k). Secretion of IL-17A, IL-2, and GM-CSF was also reduced at the peak of EAU (Fig. 3l, m; Supplementary information, Fig. S5f). These data reveal that auto-phosphorylation of the PQR cluster is required for the interaction of DNA-PKcs with RORγt, thereby sustaining the effector function of Th17 cells.

### IL-23 increases the kinase activity of DNA-PKcs to stabilize RORγt activity and promote DSB ligation

**Fig. 4:**
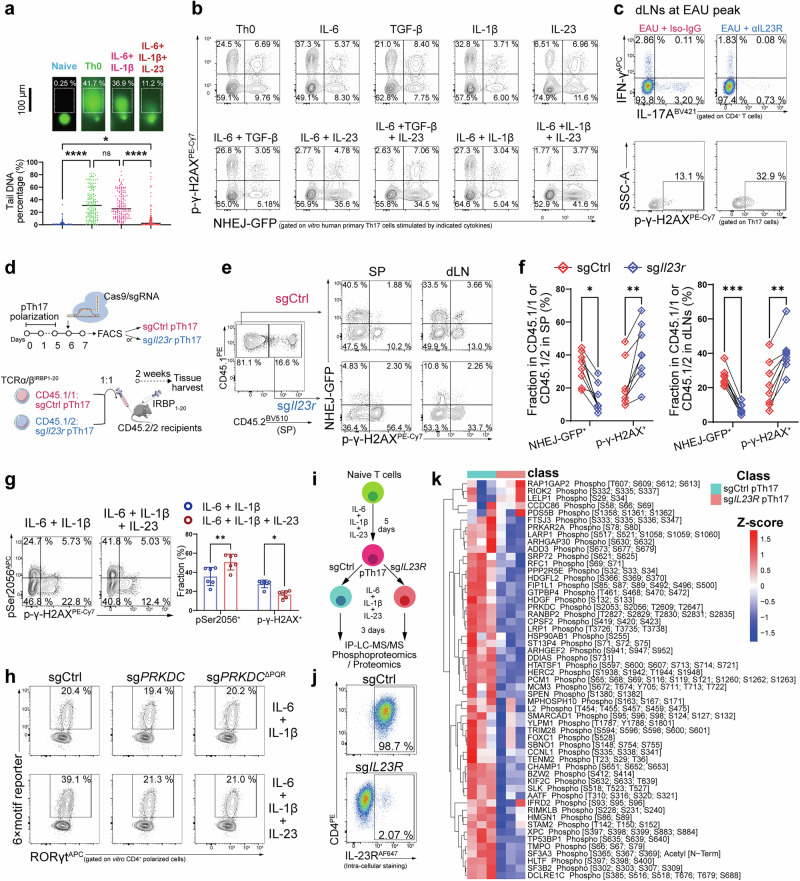
IL-23R signaling increases DNA-PKcs kinase activity. **a** Representative images and statistical graphs of a comet assay showing the extent of DSBs in CD4^+^ T cells after 5-day polarization from human naïve T cells with the indicated cytokine treatments (scale bar: 100 μm). The experiment was repeated three times, and 50 individual cells were analyzed each time (*n* = 150). **b** NHEJ assay showing the NHEJ activity and p-γ-H2AX^+^ fraction in CD4^+^ T cells after 5 days of polarization from human naïve T cells with the indicated treatments (*n* = 3). **c** FC analysis gated on CD4^+^ T cells showing the fraction of Th17 and p-γ-H2AX^+^ cells in lymph organs of EAU mice treated with 1 mg/kg/day anti-IL23R neutralizing antibody and the equivalent iso-IgG (*n* = 6). **d** Experimental scheme showing the adoptive transfer of CD45.1/1^+^ sgCtrl pTh17s and CD45.1/2^+^ sg*Il23r* pTh17 into CD45.2/2^+^ EAU recipients. Data were combined from 2 independent experiments with *n* = 8. **e** NHEJ assay showing the NHEJ activity and p-γ-H2AX^+^ fraction of transferred pTh17s in dLNs and SPs from the EAU recipients in **d** (*n* = 8). **f** Statistical graphs for **e**. **g** FC analysis to examine the effect of IL-23 on the expression of pSer2056-DNA-PKcs and p-γ-H2AX in human primary (HP) Th17 cells (*n* = 6). **h** FC analysis to examine the effect of IL-23 on the expression of the RORγt 6× motif reporter in human CD4^+^ T cells polarized from naïve T cells with the indicated cytokine treatments (*n* = 4). **i** Experimental scheme to examine the phosphorylation level of DNA-PKcs-binding proteins in sgCtrl and sg*IL23R* pTh17 after 5-day polarization from human naïve T cells. **j** FC analysis verifying the expression of IL-23R on sgCtrl and sg*IL23R* pTh17 (*n* = 3). **k** Heatmap showing the level of phospho-enriched peptides detected by LC-MS in sgCtrl and sg*IL23R* pTh17 cells (*n* = 3). Significance was assessed by paired or unpaired Student’s *t*-test or one-way analysis of variance, followed by Tukey’s test or two-way analysis of variance, followed by Bonferroni’s test. Error bars represent mean ± SD. ^*^P < 0.05, ^**^P < 0.01, ^***^P < 0.001, ^****^P < 0.0001. See also Supplementary information, Fig. S6.

We next explored why NHEJ was highly activated in pTh17 and how this activation affected pTh17. In general, Th17 differentiation begins when naïve precursors receive T cell receptor (TCR)/CD28 signals in the presence of a cytokine environment dominated by IL-6, TGF-β, IL-1β, and IL-23, among which IL-23 largely determines the pathogenicity of Th17 cells.^27^ Indeed, we found that application of IL-23 reduced the intensity of DSBs (Fig. 4a). An NHEJ assay also revealed that IL-23 treatment increased the percentage of GFP^+^ cells and reduced the fraction of p-γ-H2AX^+^ cells (Fig. 4b), indicating enhanced repair efficiency and attenuated DSB formation. Treating EAU mice with anti-IL-23R antibody also reduced the Th17 fraction and induced greater DSB accumulation in the remaining Th17 cells (Fig. 4c). After 1:1 adoptively transferring WT and *Il23r*^KO^ pTh17 cells to EAU recipients, we found that *Il23r*-deficient pTh17 were defective in NHEJ activation and showed greater DSB accumulation (Fig. 4d–f). Also, application of recombinant IL-23 alleviated DSB formation in WT and native Th17 cells but did not function normally in *Il23r*^KO^ Th17 cells (Supplementary information, Figs. S6a–c and S11e). These data collectively confirmed that IL-23 increases NHEJ activity to enable resistance to DSB accumulation. In addition, IL-23 promoted auto-phosphorylation of Ser2056 at the PQR cluster (Fig. 4g). Using co-IP and the RORγt 6× motif reporter assay, we found that IL-23 promoted RORγt stabilization, which was dependent on the PQR cluster (Fig. 4h; Supplementary information, Fig. S6d), indicating that IL-23 also supported RORγt activity via the PQR cluster.

As mentioned above (Supplementary information, Fig. S2), the NHEJ ligation process did not sustain the differentiation of pTh17 cells. It was important to clarify why an increase in DNA ligation could be seen when we polarized pTh17 cells using IL-23. A plausible explanation was that IL-23 promoted the activity of the DNA-PKcs kinase domain, which not only enabled auto-phosphorylation of DNA-PKcs for RORγt stabilization, as we demonstrated (Fig. 3), but also promoted phosphorylation of the downstream complex for end processing. We therefore pulled down DNA-PKcs in *IL23R*-KO pTh17 cells and used IP-LC-MS phospho-proteomics to examine changes in phosphorylation levels of DNA-PKcs-binding proteins. We found that ablation of IL-23R inhibited phosphorylation of the majority of pulled-down peptides (Fig. 4i–k). Among them, DCLRE1C (also called Artemis) is the most important NHEJ protein required for resecting the blunt ends of DSBs to enable the ligation process to proceed after phosphorylation by DNA-PKcs.^22^ We therefore concluded that IL-23R signaling enhances DNA-PKcs kinase activity, which stabilizes RORγt transcriptional activity to drive Th17 pathogenicity. Independently, IL-23-induced DNA-PKcs activation also promotes DSB ligation to maintain genomic stability. Although both pathways originate from IL-23-mediated activation of DNA-PKcs, stabilization of RORγt specifically determines the pathogenic function of Th17, whereas DSB ligation is a separate functional outcome.

### IL-23R signaling induces IER2 to maintain the kinase activity of DNA-PKcs

**Fig. 5:**
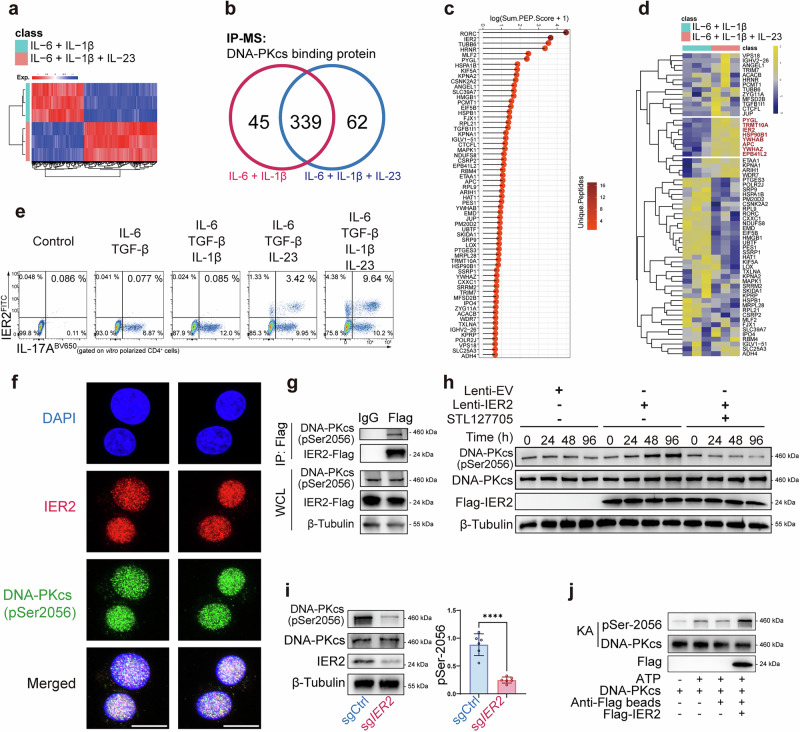
IL-23R-induced IER2 is required for maintenance of DNA-PKcs auto-phosphorylation. **a** Heatmap showing the variation in gene expression in response to IL-23 stimulation (*n* = 3). **b** Venn diagram for IP-MS showing the IL-23R-induced proteins with which DNA-PKcs interacted. **c** Lollipop chart for IP-MS listing the IL-23-induced proteins with which DNA-PKcs interacted. **d** Heatmap showing the expression variation of genes encoding the proteins in (**a**). Genes that were significantly upregulated are labeled in red. **e** FC analysis showing the fraction of human IER2^high^ Th17 cells under the indicated polarizing conditions (*n* = 3). **f** Immunofluorescence assay to detect the co-localization of IER2 (red) and pS2056-DNA-PKcs (green) in polarized human pTh17 cells (scale bar: 10 μm). **g** Immunoblot for co-IP assay showing that FLAG-IER2 interacted with p-DNA-PKcs in HP Th17 overexpressing ectopic IER2 (*n* = 3). **h** Immunoblot for pSer2056-DNA-PKcs expression in HP Th17 transfected with FLAG-IER2, with or without STL127705 (*n* = 3). **i** Immunoblot for pSer2056-DNA-PKcs expression in polarized human pTh17 cells transduced with a CRISPR system targeting *IER2* (*n* = 6). **j** Immunoblot for an in vitro kinase assay showing auto-phosphorylation of DNA-PKcs in the presence of IER2 and ATP (*n* = 3). Significance was assessed by unpaired Student’s *t*-test or one-way analysis of variance, followed by Tukey’s test or two-way analysis of variance, followed by Bonferroni’s test. Error bars represent mean ± SD. ^*^P < 0.05, ^**^P < 0.01, ^***^P < 0.001, ^****^P < 0.0001. See also Supplementary information, Fig. S7.

To determine how IL-23R signaling activates DNA-PKcs, we first identified genes and proteins that were responsive to IL-23. RNA sequencing (RNA-seq) revealed transcriptome-wide upregulation of genes following IL-23 stimulation (Fig. 5a). In parallel, mass spectrometry (MS) screening identified 62 proteins that specifically bound DNA-PKcs in an IL-23-dependent manner (Fig. 5b, c). Integrating these datasets, we found that 8 of the 62 corresponding genes were also transcriptionally upregulated by IL-23 (Fig. 5d), making them prime candidates for mediating DNA-PKcs activation. To identify which factor interacted with DNA-PKcs functionally, we knocked out each factor and observed that ablation of IER2 reduced the auto-phosphorylation of Ser2056 in the PQR cluster (Supplementary information, Fig. S7a). Consistent with our RNA-seq data, an in vitro pTh17 differentiation assay suggested that IL-23 was required for the induction of IER2^high^ Th17, whereas IL-1β facilitated this process but was dispensable (Fig. 5e; Supplementary information, Fig. S7b).

The molecular interaction between IER2 and DNA-PKcs was further confirmed through immune-fluorescence staining, which showed that IER2 co-localized with auto-phosphorylated DNA-PKcs in human polarized pTh17 cells (Fig. 5f). Results from a co-IP assay also supported this finding (Fig. 5g). In addition, ectopic expression of IER2 promoted the auto-phosphorylation of DNA-PKcs in a time-dependent manner, and IER2 knockout failed to maintain this effect (Fig. 5h, i). We confirmed that IER2 enhanced the auto-phosphorylation of DNA-PKcs using an in vitro kinase assay (Fig. 5j). We also observed that ectopic expression of IER2 protected T cells from DSBs, and this effect could be reversed by DNA-PKcs inhibition (Supplementary information, Fig. S7c). Overall, these data demonstrated that IER2 is a novel NHEJ factor that transduces IL-23R signaling to increase the kinase activity of DNA-PKcs.

### IER2 promotes Th17 effector function

**Fig. 6:**
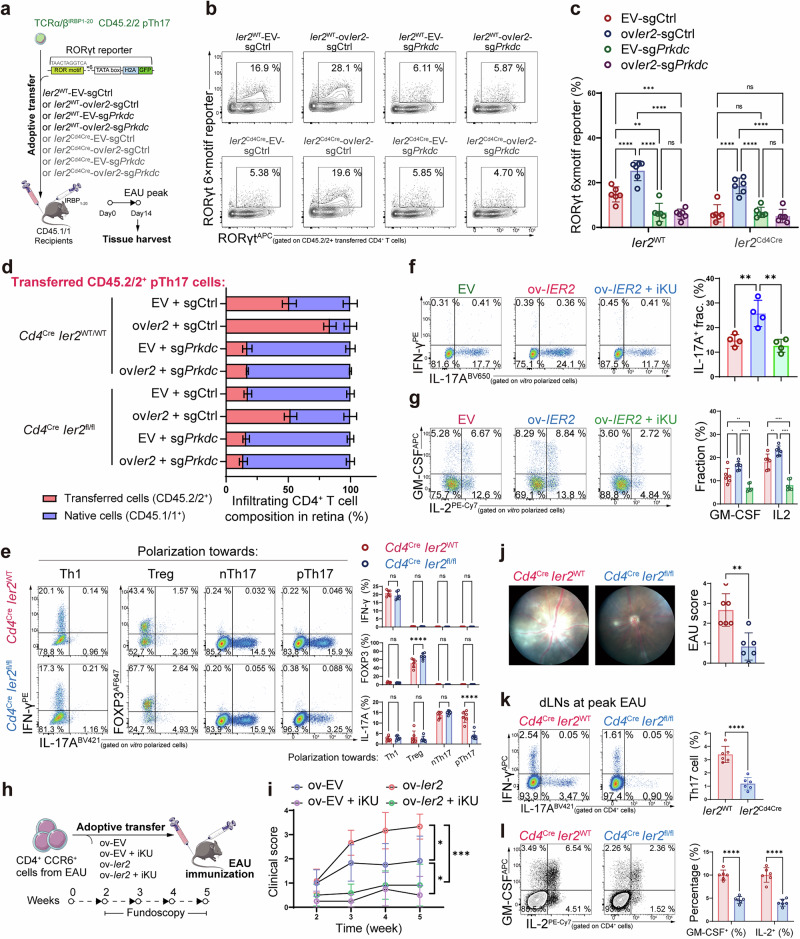
IER2 sustains the pathogenicity of Th17 cells in EAU. **a** Experimental scheme to examine the role of IER2 in the maintenance of RORγt transcriptional activity during EAU. WT pTh17 (*Cd4*^Cre^ *Ier2*^WT^/CD45.2/2) or *Ier2*^CKO^ pTh17 (*Cd4*^Cre^ *Ier2*^fl/fl^/CD45.2/2) were transduced with *Ier2* or the empty vector (EV) or a CRISPR system targeting *Prkdc* or its sgRNA control (sgCtrl). The cells were transferred into EAU recipients (CD45.1/1). Data were combined from two independent experiments with *n* = 6. **b** FC analysis gated on transferred CD45.2/2^+^ cells showing the expression of the RORγt 6× motif reporter in the EAU recipients from **a** (*n* = 6). **c** Statistical graph for **b**. **d** Statistical chart showing the ratio of the transferred CD45.2/2^+^ pTh17 fraction to native CD45.2/2^+^ CD4^+^ T cells in the retina (*n* = 6) from the experiment in (**a**). **e** FC analysis showing the differentiation of Th1, Treg, nTh17, and pTh17 after 5 days of polarization from *Cd4*^Cre^ *Ier2*^WT^ or *Cd4*^Cre^ *Ier2*^fl/fl^ murine naïve CD4^+^ T cells (*n* = 6). Data were combined from two independent experiments with *n* = 6. **f** FC analysis showing the fraction of IL-17A^+^ in human naïve CD4^+^ T cells after a 5-day induction of pTh17 cells (*n* = 4). **g** FC analysis showing the secretion of GM-CSF and IL-2 after 5 days of induction of human pTh17 cells (*n* = 6). **h** Experimental scheme to examine the role of IER2 in Th17 effector function by adoptive transfer of pTh17 transduced with an ectopic *Ier2*-overexpression vector to EAU *Rag1*^−/−^ mice, followed by fundoscopic examination for 5 weeks. Data were combined from two independent experiments with *n* = 6. **i** Statistical graph for the EAU fundoscopic scores in **h** (*n* = 6). **j** Fundoscopic images showing the ocular fundus of *Cd4*^Cre^ *Ier2*^WT^ and *Cd4*^Cre^ *Ier2*^fl/fl^ mice established with EAU, with the related statistical graph (*n* = 6). Data were combined from 2 independent experiments with *n* = 6. **k** FC analysis gated on CD4^+^ T cells showing the fraction of Th17 in dLNs of *Cd4*^Cre^ *Ier2*^WT^ and *Cd4*^Cre^ *Ier2*^fl/fl^ mice established with EAU (*n* = 6). **l** FC analysis gated on CD4^+^ T cells showing the fraction of IL-2^+^ and GM-CSF^+^ cells in dLNs of *Cd4*^Cre^ *Ier2*^WT^ and *Cd4*^Cre^ *Ier2*^fl/fl^ mice established with EAU (*n* = 6). Significance was assessed by unpaired Student’s *t-*test or one-way analysis of variance, followed by Tukey’s test or two-way analysis of variance, followed by Bonferroni’s test. Error bars represent mean ± SD. Correlations between two variables were analyzed using Pearson’s test. ^*^P < 0.05, ^**^P < 0.01, ^***^P < 0.001, ^****^P < 0.0001. See also Supplementary information, Fig. S8.

We next examined whether IER2 sustained the pathogenicity of pTh17 cells via the DNA-PKcs–RORγt interaction. IER2 deficiency reduced the interaction between DNA-PKcs and RORγt, as well as the expression of the RORγt motif reporter (Supplementary information, Fig. S8a, b). To examine the mechanism in vivo, we generated mice with TCRα/β T cell-specific ablation of IER2 by crossing *Ier2*^fl/fl^ mice with *Cd4*^Cre^ mice. *Cd4*^Cre^ *Ier2*^fl/fl^ mice were indistinguishable from their littermate controls and displayed normal T-cell development and unbiased Th subsets at steady state (Supplementary information, Fig. S8c–f). We then adoptively transferred CD45.2/2^+^ *Cd4*^Cre^ *Ier2*^fl/fl^ pTh17 cells carrying the RORγt 6× motif reporter into CD45.1/1^+^ EAU mice (Fig. 6a). As expected, IER2 ablation markedly reduced reporter expression and alleviated retinal infiltration, whereas re-expressing IER2 in *Cd4*^Cre^ *Ier2*^fl/fl^ cells rescued the loss of RORγt transcriptional activity and retinal infiltration, both of which were abrogated by DNA-PKcs knockout (Fig. 6b–d). These in vivo and in vitro data demonstrate that IER2 sustains RORγt transcriptional activity in a DNA-PKcs-dependent manner.

We then asked whether IER2 was involved in the pathogenic program of Th17 cells. In vitro data showed that IER2 ablation impaired pTh17 differentiation (Fig. 6e; Supplementary information, Fig. S8i). In addition, overexpression of IER2 promoted pTh17 differentiation, as well as secretion of IL-2 and GM-CSF (Fig. 6f, g; Supplementary information, Fig. S8g, h). Both KU inhibition and *PRKDC* knockout reversed this process, suggesting that NHEJ-dependent DSB sensing was required. In vivo, transfer of IER2-overexpressing primary Th17 cells into *Rag1*^−/−^ EAU mice exacerbated EAU inflammation at both peak intensity and duration, which could be reversed by KU blockade (Fig. 6h, i). In addition, *Rag1*^−/−^ mice reconstituted with IER2-deficient CD4^+^ T cells were nearly resistant to EAU pathology (Supplementary information, Fig. S8j–l). We next induced EAU in *Ier2*-CKO mice and littermate controls to observe their clinical scores, and we examined recently defined in vivo pTh17 markers in each group (CXCR6^+^Ly108^−^).^3,28^ As expected, *Cd4*^Cre^*Ier2*^fl/fl^ mice exhibited lower EAU clinical scores, with a lower CXCR6^+^Ly108^−^ Th17 fraction and reduced secretion of IL-17A, IL-2, and GM-CSF (Fig. 6j–l; Supplementary information, Fig. S8m, n). These findings confirmed that IER2 sustains the pathogenicity of Th17 cells and that this process is mediated by DNA-PKcs.

### IER2^high^ Th17 orchestrates a pathogenic subset in autoimmune uveitis patients

**Fig. 7:**
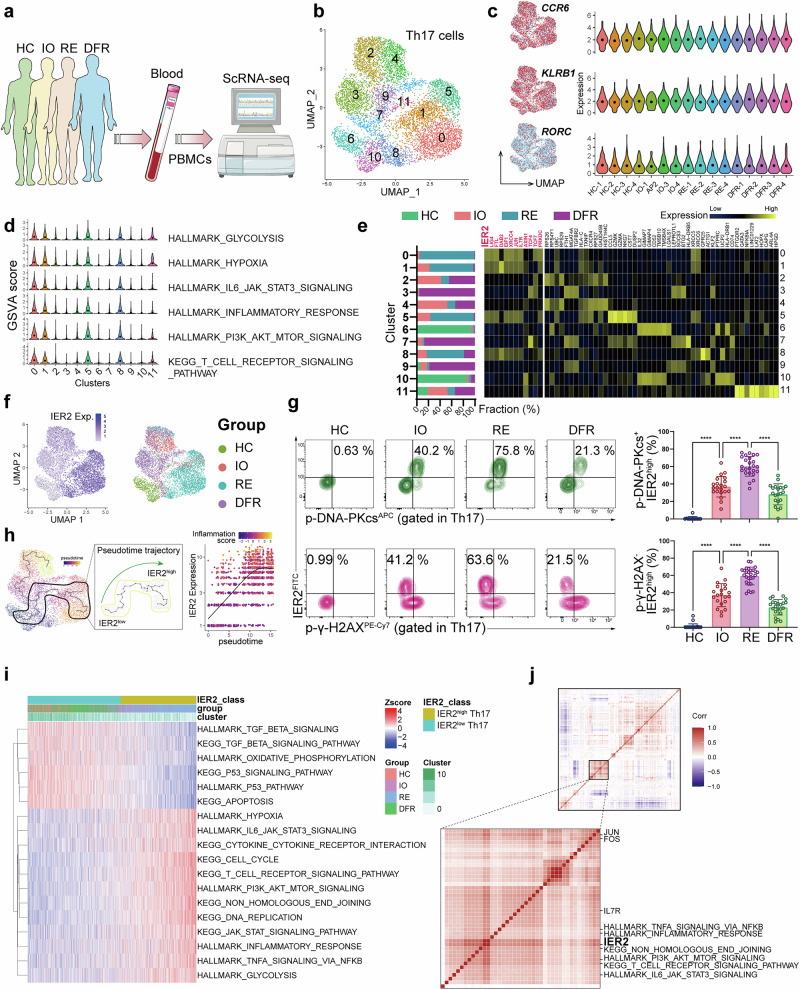
Single-cell transcriptomics reveals the heterogeneity of Th17 cells in PBMC samples from AU patients. **a** Schematic diagram demonstrating the overall research design. PBMCs collected from 12 VKH patients and 4 healthy donors were used for scRNA-seq. **b** UMAP plot demonstrating subclusters of primary Th17 cells from healthy donors and the 3 AU groups in the scRNA-seq data (*n* = 4). **c** UMAP plots and violin plots showing the expression of Th17 hallmark genes (*CCR6*, *KLRB1*, and *RORC*) in primary Th17 cells from the human scRNA-seq data. **d** Violin plot for GSVA analysis showing the activity of inflammatory pathways in each Th17 cluster. **e** Bar plot and heatmap showing the composition and DEGs in each cluster. **f** UMAP plots showing the cell distribution of each group and the expression of *IER2*. **g** FC analysis showing the proportion of IER2^high^ pSer2056-DNA-PKcs^+^ cells (HC *n* = 12; IO *n* = 21; RE *n* = 24; DFR *n* = 21) and IER2^high^ p-γ-H2AX^−^ cells in PBMC samples from 4 groups (HC *n* = 26; IO *n* = 21; RE *n* = 27; DFR *n* = 22). **h** Pseudotime trajectory analysis showing the correlation of *IER2* expression with the GSVA score for the inflammatory response along the trajectory from the IER2^low^ to the IER2^high^ subset in the scRNA-seq data. **i** Heatmap showing the functional differences in IER2^high^ Th17 and IER2^low^ Th17 (cut-off by the median of *IER2* expression) analyzed by GSVA. **j** Correlation heatmap showing the close relationship between IER2 gene expression and GSVA scores for inflammatory pathways in the scRNA-seq data. Significance was assessed by one-way analysis of variance followed by Tukey’s test. Error bars represent mean ± SD. ^****^P < 0.0001. See also Supplementary information, Fig. S9.

To characterize the heterogeneity of the Th17 subset from patients with autoimmunity, we performed scRNA-seq of peripheral blood mononuclear cells (PBMCs) from healthy control (HC) and autoimmune uveitis (AU) patients at different stages, including initial onset (IO), refractory relapse (RE), and drug-free remission (DFR) (Fig. 7a; Supplementary information, Table S1). Because *IL17A* and *IL17F* transcript levels were lower in unstimulated T cells, we used general Th17 markers, including *CCR6*, *KLRB1*, and *RORC*, to distinguish the Th17 cells. A total of 11 Th17 clusters were identified using dimensionality reduction by uniform manifold approximation and projection (UMAP), and expression of *CCR6*, *KLRB1*, and *RORC* did not differ significantly among cells from different samples (Fig. 7b, c). To identify the pathogenic subsets, we used gene set variation analysis (GSVA) to quantify the pathway activity in each individual cell across the 11 clusters. We found that clusters of 0, 1, 5 and 8 displayed higher activation of glycolysis, IL-6-STAT3 signaling, PI3K-AKT signaling, and TCR signaling, suggesting that these clusters might have a stronger Th17-related effector function (Fig. 7d). The cells in these clusters were mainly derived from RE patients, consistent with the most severe clinical outcomes (Fig. 7e). Cells from these clusters also showed higher expression of *IER2* and two NHEJ factors, *XRCC4* and *PRKDC* (Fig. 7e). By contrast, clusters 3, 7 and 9 were identified as “non-pathogenic” because of their higher expression of *TGFBR2*; these were mainly derived from DFR patients with the best clinical outcome (Fig. 7e).

The UMAP plot suggested that the IER2^high^ Th17 subset was found mainly in the RE group (Fig. 7f). We verified *IER2* expression in Th17 cells from PBMC samples of the HC donors and AU patients at each stage. The RE group had the highest Th17 proportion with the most *IER2* expression, and expression of *IER2* was associated with pSer2056-DNA-PKcs and lower p-γ-H2AX formation (Fig. 7g; Supplementary information, Fig. S9a). In addition, analysis of the pseudotime trajectory also supported the finding that IER2^high^ Th17 was more pathogenic, because the inflammation score increased along the trajectory from “IER2^low^” to “IER2^high^” (Fig. 7h). Compared with its IER2^low^ counterpart, IER2^high^ Th17 was more active in the inflammatory response (Fig. 7i). A receptor–ligand-based cellular communication network also supported the finding that IER2^high^ Th17 was more active in chemokine signaling (Supplementary information, Fig. S9b). Next, we used Spearman correlations of cellular transcriptomes based on “ward.D” clustering to identify the functional module of Th17 cells. This analysis showed that IER2 was located in the core module that had the closest correlation with the NHEJ and T cell activation pathways (Fig. 7j; Supplementary information, Fig. S9c).

Collectively, these data reveal that IER2^high^ Th17 cells are present in autoimmune patients and are related to unfavorable clinical outcomes, suggesting that they could serve as a clinical biomarker for disease progression.

## Discussion

Here, we report a novel function of the NHEJ process in the adaptive immune system. Although DSB-end ligation is dispensable for the activation, differentiation, and effector function of Th17 cells, KU-dependent DSB sensing plays a critical role in maintaining the pathogenicity of Th17 cells in vitro and in animal models of uveitis and colitis. At the molecular level, we found that the NHEJ factor DNA-PKcs receives KU-transduced DSB signals and stabilizes the binding of RORγt to effector-gene loci to induce autoimmunity, a process that is dependent on auto-phosphorylation of DNA-PKcs at the PQR cluster. We also identified a novel NHEJ factor, IER2, that is induced by IL-23R signaling and strengthens the kinase activity of DNA-PKcs, not only initiating the ligation process but also sustaining DNA-PKcs auto-phosphorylation to stabilize RORγt and thus trigger autoimmunity. Our study highlights a mechanism by which perception of DSBs by NHEJ directly controls Th17 pathogenicity in autoimmunity. Our findings also reveal a complex interaction between the DNA repair system and T cell response in the mature adaptive immune system.

We initially noticed that T cells display heterogeneity in the intensity of DSBs after polarization with different induction cytokines. We found that pTh17 cells showed more pronounced NHEJ activity and greater resistance to DSB accumulation than their non-pathogenic counterparts. Because only pTh17 possessed the ability to trigger autoimmunity, it is tempting to speculate that NHEJ could serve as another checkpoint for Th17 pathogenicity in autoimmune disease, apart from aspects of energy metabolism, such as glycolysis.

Previous research on cancer immunology indicates that a persistent CD8^+^ T cell response requires an efficient ability to repair DSBs because it eliminates the DSB-induced cell death signals and retains the potential for further clonal expansion.^20,29–32^ However, this is quite different from the mechanism in Th17 cells, whose pathogenicity is strongly reliant on KU-dependent DSB perception rather than DSB repair. A recent study demonstrated that the KU complex, rather than the ligation complex, is required for the function of total CD4^+^ T cells in autoimmunity.^17^ In the present study, we found that ablation of the DSB sensor KU80 eliminated the effector function of pTh17 cells, rather than other Th subsets. Moreover, we found that ablation of the final step of NHEJ, DSB ligation, did not affect the pathogenicity, differentiation, or even cell activation of pTh17 cells. DSBs have been reported to form mainly at telomere ends in auto-reactive CD4^+^ T cells, whereas the coding sequences of effector gene loci on the chromosomes are less affected, thus maintaining the cells’ expansion potency and effector functions even when carrying DSB burdens in autoimmunity.^33^ Therefore, we conclude that DSB sensing, rather than DSB ligation, is required for the effector function of Th17 cells to trigger autoimmunity.

RORγt is the master TF that controls Th17 cell differentiation and pathogenicity, but it requires cooperative factors such as STAT3, Blimp-1, and HIF-1α to fully orchestrate its transcriptional program.^34–36^ Our findings demonstrate that auto-phosphorylation of DNA-PKcs at the PQR cluster, in addition to acting as a prerequisite for its disengagement from DSB sites, enables its direct molecular interaction with RORγt. This interaction, together with its intrinsic DNA affinity, significantly stabilizes the binding of RORγt to key effector genes while facilitating chromatin accessibility at these loci. In addition, this interaction does not alter RORγt expression but enhances its transcriptional efficacy, thereby increasing the pathogenicity of pTh17 cells. Consequently, our study reveals a model in which auto-phosphorylated DNA-PKcs serves as a direct molecular intermediary, translating KU complex-derived signals into potentiation of the RORγt-driven transcriptional program that defines Th17 pathogenicity.

Th17 polarization is initiated by IL-6, TGF-β, IL-23, and other inflammatory cytokines. Among them, IL-23 is required for the stabilization and expansion of pathogenic Th17 cells.^37^ IL-23R-deficient Th17 cells show impaired survival and fail to induce autoimmune inflammation in vivo, as demonstrated in EAU and EAE models.^38,39^ Elevated IL-23 levels can be seen in the blood samples from active VKH patients.^40^ Targeting the p19 subunit of IL-23 using guselkumab showed favorable therapeutic efficacy in the treatment of psoriasis.^41^ In addition to its immune functions, IL-23 also enhances DNA repair in non-immune tissues.^42,43^ Our study demonstrates that IL-23 significantly promotes NHEJ activity in T cells. This effect depends on the presence of IL-6, as IL-6 upregulates *IL-23R* expression, which is relatively low in steady-state T cells. This explains why IL-23 stimulation alone fails to fully recapitulate the robust NHEJ phenotype observed under pTh17-polarizing conditions containing both cytokines. We also found that IL-23 enhances the transcriptional function of RORγt in pTh17 cells by increasing DNA-PKcs auto-phosphorylation. In other words, IL-23R acts as an “accelerator” for this process, thus promoting pTh17 differentiation. However, given that Ligase IV is not involved in pTh17 differentiation, it is difficult to understand why application of IL-23 would improve ligation efficiency. In fact, these two findings are not contradictory. Our data show that IL-23R signaling increases the kinase activity of DNA-PKcs. This not only increases PQR autophosphorylation to support the RORγt transcriptional program but also increases the phosphorylation of DSB-processing proteins to enable ligation, which are two totally independent processes separately mediated by DNA-PKcs.

Our study also indicates that IL-23 induces IER2 expression to increase the kinase activity of DNA-PKcs. IER2 is a nuclear protein that belongs to the immediate-early-response family and is named for its rapid expression in response to various extracellular stimuli.^44^ In cancer cells, IER2 drives tumor progression and metastasis.^45^ IER2 contains nuclear localization signals and may possess a transcriptional regulation function.^45,46^ In our study, IER2 increased DNA-PKcs kinase activity; this process not only enhances auto-phosphorylation of the PQR cluster, thus maintaining the interaction of DNA-PKcs with RORγt and stabilizing the transcriptional program, but also enables DSB ligation and protects Th17 cells from DSB accumulation. Moreover, observations from clinical samples also confirmed the existence of IER2^high^ Th17 cells, which exhibited pronounced inflammatory-response signatures. The proportion of IER2^high^ cells was significantly higher in AU patients during the relapse phase.

Whereas most studies have focused on the role of glycolysis in regulating Th17 effector function, we performed a comprehensive study of the NHEJ system, which may serve as a checkpoint for maintaining Th17 pathogenesis in autoimmunity. Our study also provides significant insights into the mechanism by which RORγt maintains stable affinity for effector-gene loci, thus enabling the differentiation of pathogenic Th17 cells. Mechanistically, KU-dependent DSB perception activates DNA-PKcs, which stabilizes the binding of RORγt to pro-inflammatory gene loci, thereby exacerbating autoimmune inflammation. This process is intensified by IL-23R signaling, which induces the expression of *IER2* to increase DNA-PKcs kinase activity. IER2 not only stabilizes the RORγt transcriptional program for pathogenicity but also alleviates DSB accumulation to promote cell survival. Therefore, selective interference with the NHEJ-dependent process of DSB perception might provide therapeutic strategies for patients with autoimmune diseases.

## Materials and methods

### Mouse strains

C57BL/6J (Strain No. N000013), H11-Cd4-iCre (C57BL/6JGpt-H11^em1Cin(*Cd4*-iCre)^/Gpt, Strain No. T004818), Ier2-flox (C57BL/6JGpt-*Ier2*^em1Cflox^/Gpt, Strain No. T020886), and B6-Rag1-KO mice (C57BL/6JGpt-*Rag1*^em1Cd3259^/Gpt, Strain No. T004753) were purchased from GemPharmatech (Nanjing, China). Homozygous CD45.1/1^+^ mice were purchased from Zhiyuan Biopharmaceutical Technology Co., Ltd (Guangzhou, China). CD45.1/2^+^ heterozygous mice were generated by mating CD45.1/1^+^ mice with C57BL/6J mice (CD45.2/2^+^). All transgenic mice were in the C57BL/6J background (female, 6–8 weeks old, 20 ± 1.5 g). All mice were housed in a specific pathogen-free environment at the animal facility of the Animal Laboratories of Zhongshan Ophthalmic Center with a 12-h light-dark cycle, a controlled temperature of 23 ± 2°C, and a constant humidity of 55% ± 10%. All animal procedures were approved by the Institutional Animal Care and Use Committee of Zhongshan Ophthalmic Center, Sun Yat-sen University (ID: O2023009). All animal care was performed in strict accordance with the guidelines of the Association for Research in Vision and Ophthalmology (ARVO) Statement for the Use of Animals in Ophthalmic and Vision Research.

### Human donors

Patients who were diagnosed with Vogt–Koyanagi–Harada syndrome (VKH), a subtype of AU, were enrolled in this study. VKH was diagnosed on the basis of the revised diagnostic criteria.^47^ Patients were classified into three sub-groups according to disease phase. Patients in the IO group were all initially diagnosed and had not received any therapy. Patients in the RE group were under a continuous immunosuppressive course but had newly occurring ocular inflammatory symptoms. Those in the DFR group had stable visual function and had not been under immunosuppressive treatment for more than 1 year, based on knowledge of immune tolerance in renal transplantation.^48^ Donors with no ocular or other systemic illnesses were enrolled in the HC group. Detailed clinical information was collected after informed consent and is presented in Supplementary information, Tables S1 and S2. All information collection and operation procedures were performed in accordance with the Declaration of Helsinki and were approved by the Medical Ethics Committee of the Guangzhou Zhongshan Ophthalmic Center (ID: 2020KYPJ104).

### In vitro T cell culture

To obtain human T cells for in vitro assays, fresh heparinized venous blood samples were collected from AU patients or healthy donors after they had signed an informed consent form. PBMCs were isolated from the blood samples using Ficoll-Hypaque density gradient centrifugation. To isolate and culture human primary (HP) Th17 cells, the PBMCs were washed with PBS and stained with PE-anti-human CD4 (BioLegend) and PerCP/Cyanine5.5-anti-human CD161 (BioLegend) antibodies for 20 min. HP Th17 cells were sorted using a high-speed sorter (FACSAria, BD Biosciences) with the CD4^+^ CD161^+^ phenotype^49^ and were defined by IL-17A expression with high purity (> 80.0%, see Supplementary information, Fig. S10d). HP Th17 cells were cultured in RPMI 1640 medium (Gibco) containing 10% fetal bovine serum (FBS, Gibco), 1 mM sodium pyruvate, 50 nM β-mercaptoethanol, and MEM non-essential amino acids in humidified air with 5% CO_2_ at 37 °C. The cells were cultured at a density of 2 × 10^5^/well on a 96-well plate, and 0.25 × 10^5^ anti-CD3/CD28 beads/well were added (Invitrogen). In some experiments, HP Th17 cells were pre-transfected with a corresponding lentivirus and were further cultured for 3 days for functional assays.

For polarization of human T helper (Th) cells, naïve CD4^+^ T cells were negatively sorted from human PBMCs using the EasySep Human Naïve CD4^+^ T Cell Isolation Kit II (STEMCELL). They were cultured in complete RPMI medium at a density of 2 ×  10^5^/well on a 96-well plate and activated by the addition of 0.25  × 10^5^ anti-CD3/CD28 beads/well. Naïve CD4^+^ T cells were polarized in the presence of the following cytokines and neutralizing antibodies for 5 days. For Th1 cells: 5 ng/mL rhIL-12 (p70) (BioLegend) and 2 μg/mL anti-hIL-4 (R&D Systems). For Treg cells: 10 ng/mL rhTGF-β1 (PeproTech). For “non-pathogenic” Th17 cells (nTh17): 80 ng/mL rhIL-6 (PeproTech), 10 ng/mL rhTGF-β1 (PeproTech), 2 μg/mL anti-hIFN-γ (R&D Systems), and 2 μg/mL anti-hIL-4. For “pathogenic” Th17 cells (pTh17): 80 ng/mL rhIL-6, 20 ng/mL rhIL-1β (PeproTech), 20 ng/mL rhIL-23 (PeproTech), 2 μg/mL anti-hIFN-γ, and 2 μg/mL anti-hIL-4. On day 3, the medium was refreshed and supplemented with the corresponding cocktails. In some experiments involving the CRISPR/Cas9 strategy, human naïve CD4^+^ T cells were isolated from PBMCs and seeded into RetroNectin-pre-coated 96-well plates at a density of 2 × 10^5^/well with anti-CD3/28 beads overnight. Then, lentivirus-packaged sgRNA/Cas9 was introduced into the medium and cultured for 1 day. The plates were then centrifuged, and the supernatant was discarded, followed by replacement with 1640 complete medium containing 80 ng/mL rhIL-6, 20 ng/mL rhIL-1β, 20 ng/mL rhIL-23, 2 μg/mL anti-hIFN-γ, and 2 μg/mL anti-hIL-4. The medium was refreshed after 3 days. Two days later, the differentiation level was tested by FC assay.

For culture and polarization of murine T cells, mouse T cells were first isolated from draining lymph nodes (dLNs) and spleens (SPs) and were purified by sorting live CD4^+^ CD44^−^ CD62L^+^ cells using the FACSAria Fusion sorter (BD Biosciences). The cells were cultured in complete RPMI medium in the presence of 5 μg/mL anti-mCD28 (BioLegend) at a density of 2 × 10^5^/well on a 96-well plate pre-coated with 5 μg/mL anti-mCD3ε antibody (BioLegend). The cells were polarized under the following conditions for 5 days. For Th1 cells: 5 ng/mL rmIL-12 (p70) (BioLegend) and 2 μg/mL anti-mIL-4 (BioLegend). For Treg cells: 10 ng/mL rhTGF-β1 (BioLegend). For nTh17 cells: 50 ng/mL rmIL-6 (BioLegend), 10 ng/mL rhTGF-β1, 2 μg/mL anti-mIFN-γ (BioLegend), and 2 μg/mL anti-mIL-4. For pTh17 cells: 50 ng/mL rmIL-6, 50 ng/mL IL-1β (BioLegend), 40 ng/mL rmIL-23 (BioLegend), 2 μg/mL anti-mIFN-γ, and 2 μg/mL anti-mIL-4. The medium was refreshed and supplemented with the corresponding cocktails on day 3.

### EAU establishment

IRBP_1–20_ peptide (2 mg/mL; GL Biochem) was emulsified with Freund’s adjuvant (Sigma-Aldrich) containing *Mycobacterium tuberculosis* strain H37Ra extract (5 mg/mL; Difco) in a 1:1 volume ratio. Each mouse was subcutaneously injected with 200 μL emulsion at two different sites of the lower flanks and on the back for immunization. Pertussis toxin (200 ng/mouse; List Biological Laboratories) was administered intraperitoneally on day 0 and day 2 post immunization. Mice were monitored at day 14 for clinical signs of EAU using a Micron IV Retinal Imaging Microscope (PHOENIX). The scores were graded as described previously.^50^ In brief, score 0: no lesion; score 0.5: few lesions or vasculitis; score 1: < 5 lesions or mild vasculitis; score 2: > 5 lesions or substantial vasculitis; score 3: large confluent lesions; score 4: retinal detachment. At the end of the experiment, the mice were sacrificed on the morning of day 15, and the eyes were isolated and fixed with 4% paraformaldehyde overnight. The samples were then dehydrated, embedded, and sectioned (4 μm) for hematoxylin and eosin (H&E) staining. The degree of retinal inflammation was measured under the microscope and quantitatively analyzed as described previously.^50^ In brief, score 0: no lesion; score 0.5: few inflammatory infiltrations; score 1: mild retinal folds; score 2: moderate retinal folds or retinal detachment; score 3: extensive retinal folds or large retinal detachment; score 4: diffuse retinal detachment.

To observe the effect of NHEJ factors on Th17 pathogenesis, TCRβ^+^ naïve CD4^+^ T cells were isolated from blank WT mice and polarized under pTh17 conditions for 5 days. The cells were further transduced with a pCDH vector encoding IRBP_1–20_-specific TCRα/β chain and a LentiCRISPR v2-mCherry vector that carried sgRNAs targeting *Xrcc5*, *Lig4*, *Prkdc*, and *Prkdc*^PQR^ through lentivirus. The sequences of the sgRNAs, shRNAs, and corresponding HDR donor templates are provided in Supplementary information, Tables S3–S5. Tet^+^ mCherry^+^ cells were collected using fluorescence-activated cell sorting (FACS) and adoptively transferred into *Rag1*^−/−^ mice (1 × 10^6^ cells/mouse) by intraperitoneal injection. After 7 days of Th17 reconstitution, the EAU model was established, and a fundoscopic examination was performed on day 14. On day 15, the mice were sacrificed, and the eye-infiltrating cells and lymphocytes were isolated for cytokine analysis using FC assay as described previously.^51^

### Autoimmune colitis

To induce autoimmune colitis, naïve CD4^+^ CD25^−^ CD45RB^high^ T cells were isolated from lymph nodes and SPs and transduced with sgCtrl and sg*Xrcc5* lentivirus; 5 × 10^6^ cells were then intraperitoneally injected into *Rag1*^−/−^ recipient mice. Autoimmune colitis was monitored each week by recording body weight and observing stool samples and rectal prolapses. After 8 weeks, mice were sacrificed, and the colon, mesenteric LNs (mLNs), dLNs, and SPs were extracted. Cell suspensions from mLNs, dLNs, and SPs were isolated for cytokine analysis through FC assay. The histological score for colitis was the sum of the epithelium score and the infiltration score and was based on a previously described scoring system.^52^ A higher score indicated more severe damage.

### Mixed bone marrow chimeras

BM mononuclear cells were isolated from the CD45.1/1^+^ or CD45.2/2^+^ donor mice by flushing the long bones. CD45.2/2^+^ BM cells were transduced with the sg*Xrcc5* Cas9^*Sell*.Promoter^ vector with Cas9 worked in mature T-lineage, and CD45.1/1^+^ BM cells were transduced with the sgCtrl Cas9^*Sell*.Promoter^ vector. To generate T^WT^/T^*Xrcc5* KO^ chimeric reconstituted mice, a total of 1 × 10^7^ transduced CD45.2/2^+^ and CD45.1/1^+^ BM cells were mixed 1:1 and adoptively transferred into 6-week-old *Rag1*^−/−^ mice that received 6 Gy radiation. Eight weeks after reconstitution, lymphocytes from the thymus, SPs, and lymph nodes were subjected to FC assay. The gating strategy can be seen in Supplementary information, Fig. S11d, e.

### Adoptive transfer of genetically modified CD45.1^+^ T cells

Naive CD4^+^ T cells were isolated from the SPs and lymph nodes of either CD45.1/1 or CD45.2/2 congenic mice using FACS. The cells were activated with plate-bound anti-CD3 (2 μg/mL) and anti-CD28 (2 μg/mL) antibodies for 12 h. Subsequently, they were transduced with a lentiviral vector expressing an IRBP_1–20_-specific TCR. TCR-transduced cells were enriched by staining with an MHC II (I-A^b^) IRBP_4–15_ tetramers and isolated using magnetic beads.

The sorted T cells were then differentiated into pTh17 cells under polarizing conditions (50 ng/mL rmIL-6, 50 ng/mL IL-1β, 40 ng/mL rmIL-23, 2 μg/mL anti-mIFN-γ, and 2 μg/mL anti-mIL-4) for 3–4 days. After polarization, genetic modifications were introduced using lentiviral CRISPR/Cas9 or shRNA systems. Successfully transfected cells were selected either by FACS or with puromycin.

On day 0 of the experiment, 1 × 10^6^ genetically modified pTh17 cells were adoptively transferred intravenously into each recipient CD45.2/2 mouse. Immediately after cell transfer, EAU was induced in the recipients by active immunization with IRBP_1–20_ peptide emulsified in complete Freund’s adjuvant, supplemented with pertussis toxin.

On day 14 post immunization, fundus photography was performed to assess retinal inflammation in vivo. Mice were then euthanized, and the dLNs, SPs, and retinas were collected for FC analysis to track the transferred cells and evaluate their phenotypes and inflammatory functions.

### Generation of MHC II (I-A^b^) IRBP_4–15_ tetramers (Tet)

MHC II (I-A^b^) IRBP_4–15_ tetramers were used to recognize T cells carrying TCRα/β specific for the IRBP_1–20_ peptide/MHC II complex. The extracellular regions of the I-A^b^ α- and β-chains were separately cloned into the pCDH-MSCV-MCS-EF1a-GFP-PURO backbone. IRBP_4–15_ (HLFQPSLVLDMA) and a flexible linker of 13 amino acids were added to the N terminus of the β-chain.^53^ To improve heterodimerization, the C terminus of the β-chain was attached to a basic leucine zipper and a 6× His-tag, and the C terminus of the α-chain was attached to an acidic leucine zipper and an Avi-tag.^54^ The two I-A^b^ chains were co-overexpressed in the murine dendritic cell line JAWSII (ATCC) using a lentiviral transduction system. Then, 5 × 10^7^ cells were lysed after protein-free culture, and the protein extracts were coated with anti-His magnetic beads (Beyotime) at 4 °C overnight. The extracts were biotinylated using BirA enzyme (Beyotime) and were released from the magnetic beads using 6× His peptide (Beyotime), followed by tetramerization with PE-conjugated streptavidin (eBioscience). The I-A^b^ tetramers were mixed with lymphocytes and related antibodies to recognize Tet^+^ TCRβ^+^ naïve CD4^+^ T cells.

### FC

Cells were washed with ice-cold PBS, stained with Zombie NIR Fixable Viability Kit (BioLegend) at 1:2000 for 15 min at 4 °C, washed with ice-cold PBS again, and incubated with fluorophore-conjugated antibodies at the recommended dilution at 4 °C for 20 min. The cells were washed with PBS again, then immediately used for FC analysis or fixed for intracellular staining. For intracellular staining, cells were fixed using Fixation/Permeabilization Buffer (Invitrogen) or BD Phosflow Fix Buffer I (BD) and washed with Permeabilization buffer (Invitrogen). Then, the cells were washed with PBS and stained with the corresponding fluorophore-conjugated antibodies at 4  °C overnight. Before FC analysis, the cells were washed and resuspended in PBS. For staining of intracellular cytokines, cells were stimulated with 1 μg/mL BFA (brefeldin A, Sigma-Aldrich), 50 ng/mL PMA (phorbol 12-myristate 13-acetate, Sigma-Aldrich), and 500 ng/mL ionomycin (Sigma-Aldrich) for 5 h at 37 °C. Then, the cells were stained with surface-marker antibodies and intracellular antibodies as described above. The antibodies are listed in Supplementary information, Table S8. For the proliferation assay, cells were labeled with Tag-It Violet Proliferation Cell Tracking Dye (1 μmol/L, BioLegend) in a water bath at 37 °C for 10 min. The labeled cells were cultured in 96-well plates at a density of 2 × 10^5^ cells/well, and 0.5 × 10^5^ /well anti-CD3/CD28 beads were added. Cells were cultured for 72 h and subjected to FC analysis.

### Lentivirus transduction

HEK293T cells were cultured in DMEM containing 10% FBS to 70%–95% density. The medium was replaced with fresh DMEM one night before lentivirus packaging. HEK293T cells were co-transfected with 10 μg expression vectors, 7.5 μg psPAX2 plasmids, and 2.5 μg pMD2.G plasmids using Trans-EXP Liposomal Transfection Reagent (TranSheep Bio Co., Ltd), and the medium was replaced with fresh DMEM after 8 h. The medium containing the lentivirus was collected at 24 h and 48 h post transfection, concentrated using Lenti-Pac Lentivirus Concentration solution (GeneCopoeia), and resuspended in RPMI 1640 medium containing 1% FBS, 1% sodium pyruvate, and 50% Opti-MEM. The suspension containing the lentivirus was stored at 4 °C for no more than 1 week. To transduce human or murine T cells with the corresponding lentivirus, cells (1 × 10^6^/well) were resuspended in 200 μL medium containing 50% Opti-MEM and seeded into 96-well plates pre-coated with RetroNectin (1 mg/mL, Takara) and 5 μg/mL anti-mCD3ε antibody overnight for pre-activation. The next morning, the medium was replaced by the lentivirus suspension at an MOI of 200 and supplemented with 8 μg/mL polybrene (MedChemExpress). The cells were cultured for 12 h. After transduction, the cells were washed three times and cultured in fresh RPMI 1640 medium for further functional assays. The gating strategies for analyzing the transduction efficiency are displayed in Supplementary information, Fig. S11a, b. In some experiments, 6× motif reporters were transduced into the T cells to reflect the transcriptional activity of particular TFs. The motif sequences were acquired from JASPAR (https://jaspar.elixir.no/) and were listed in Supplementary information, Table S6.

### NHEJ assay

Cells (1 × 10^6^) were mixed with 2 μg of corresponding pimEJ5-GFP plasmids (Addgene) and 1 unit of I-Scel restriction enzyme (Thermo Scientific) in a nucleofection system (Lonza). The cells were transfected using the Amaxa Nucleofector Ⅱ device (Lonza). The transfected cells were immediately resuspended in complete RPMI 1640 medium, and then stimulated with 50 ng/mL PMA for 5 h. NHEJ efficiency was estimated as the fraction of GFP^+^ cells using FC.

### Comet assay

Human T cells from the indicated treatments were cultured for 5 days in 96-well plates at a density of 2 × 10^5^ cells/well and 0.25  × 10^5^ anti-CD3/CD28 beads/well. In some experiments, the DSB inducer bleomycin (10 nmol/L, MedChemExpress) and the selective KU inhibitor STL127705 (1 μmol/L, MedChemExpress) were administered. After 72 h, the cells were collected and centrifuged at 1200 rpm. The supernatant was discarded, and the cell density was adjusted to 1 × 10^5^ cells/mL with PBS. The comet assay was performed using the comet assay reagent kit (Trevigen). In brief, cells were mixed with molten LMAgarose (1:10 volume ratio) at 37 °C. Then, 50 μL of the mixture was immediately pipetted onto the comet slides before solidification. The slides were incubated at 4 °C in the dark for 30 min to improve attachment. They were then placed in the comet lysis solution (Trevigen), immersed in a washing solution containing 0.445 M Tris-HCl and 0.01 M EDTA, and subjected to neutral electrophoresis at 21 V for 30 min. Finally, the slides were immersed in 0.1% SYBR solution for visualization. Photographs were captured under a fluorescence microscope (Nikon). The experiment was repeated three times independently, and 50 cells per replicate were analyzed. The photographs were analyzed with CASP software to quantify DSBs.^55^

### Immunoblot and immunoprecipitation

Total protein was isolated from cells using RIPA lysis buffer containing PMSF and protease/phosphatase inhibitors. Protein samples of whole-cell lysates were separated by sodium dodecyl sulfate-polyacrylamide gel electrophoresis (SDS-PAGE) and were transferred to polyvinylidene difluoride (PVDF) membranes (Millipore). The membranes were blocked with 2.5% nonfat dry milk at 25 °C for 2.5 h, and then incubated with primary antibodies at the indicated dilutions overnight. The antibodies are listed in Supplementary information, Table S8. The blots were visualized using a chemiluminescence imaging workstation (Tanon) and analyzed with ImageJ software.

For endogenous immunoprecipitation (IP), extracts of Th17 cells were prepared using cell lysis buffer (Cell Signaling Technology), which contained PMSF and protease/phosphatase inhibitors. Total protein was immunoprecipitated using an anti-DNA-PKcs antibody and incubated overnight with protein A+G magnetic beads (Beyotime). For tagged proteins, 293T or Th17 cells were transfected with the corresponding vectors. Flag-tagged proteins were pulled down using a mouse anti-DYKDDDDK tag monoclonal antibody (Proteintech Group), and immunoblotting was performed using a rabbit anti-DYKDDDDK tag monoclonal antibody (Proteintech Group) to avoid interference from the murine IgG heavy chain. The full gel images for the co-IP experiments are displayed in Supplementary information, Fig. S13.

### In vitro kinase assay

The Flag-tagged IER2 and DNA-PK proteins were extracted from transfected 293T cells by immunoprecipitation. The Flag-IER2 and DNA-PK solutions were mixed in the presence of kinase reaction buffer (Cell Signaling Technology) and ATP (Cell Signaling Technology). The mixture was incubated at 30 °C for 30 min, and the reaction was terminated by boiling in SDS loading buffer for 10 min. Samples were subsequently subjected to SDS-PAGE analysis.

### Immunofluorescence

pTh17 cells induced by IL-6 + IL-1β + IL-23 were suspended in PBS, fixed in 4% paraformaldehyde for 20 min, permeabilized with 0.2% Triton X-100, and blocked with 3% bovine serum albumin. Cells were stained with primary antibodies against phospho-DNA-PKcs (Ser2056) (1:40, Cell Signaling Technology) and IER2 (1:40, Proteintech), and then incubated with secondary antibodies conjugated with Alexa Fluor 488 (1:500, Cell Signaling Technology) and Alexa Fluor 594 (1:500, Cell Signaling Technology), respectively. Nuclei were counterstained with 4′,6-diamidino-2-phenylindole (DAPI). After quenching, the cells were placed on slides. Images were obtained by confocal microscopy (Zeiss) and analyzed using ZEN 2.3 software (blue edition, ZEN).

### ChIP-seq

For ChIP-seq, human pTh17 cells were polarized, and RORγt-bound DNA was pulled down and sequenced. ChIP was performed using the ChIP Assay Kit (Beyotime Biotechnology, P2078). In brief, 1 × 10^6^ cells were fixed in 1% formaldehyde at 37 °C for 10 min to crosslink the target protein with the DNA. The reaction was stopped by the addition of 125 mM glycine solution at room temperature for 5 min. The cells were washed twice with ice-cold PBS containing 1 mM PMSF, and then lysed in 250 μL SDS lysis buffer on ice for 10 min. The cell lysates were sonicated for 4 cycles at 50 W with 30% output power. After centrifugation at 12,000 rpm, 20 μL of the supernatant was treated with 1 μL 5 M NaCl and heated at 65 °C for 4 h to de-crosslink DNA and generate all-input samples. The remaining 200 μL of supernatant was incubated with anti-RORγt mouse antibody (Santa Cruz Biotechnology) or control IgG overnight at 4 °C and pulled down using protein A+G agarose beads. The agarose beads were isolated by centrifugation at 1000× *g*, and the supernatants were discarded. The agarose beads were washed with Low Salt Immune Complex Wash Buffer, High Salt Immune Complex Wash Buffer, LiCl Immune Complex Wash Buffer, and TE Buffer. The DNA was eluted with 500 μL of elution buffer, and the supernatants were collected by centrifugation at 1000× *g*. Then, 20 μL of 5 M NaCl was added, and the samples were heated at 65 °C for 4 h for de-crosslinking. The ChIP DNA extracts were prepared using a DNA Purification Kit (Beyotime). Library products of 200–500 bp were enriched, quantified, and sequenced on a NovaSeq 6000 sequencer (Illumina) to obtain 150-bp paired-end reads. The fastq files were processed with fastp to filter the raw data and then mapped to the human GRCh38 genome assembly using Bowtie2 (v0.20.0). After the generation of bam and bigwig files, the R package rtracklayer (v1.48.0) was used to analyze the distribution of reads. The peak tracks were displayed using IGV (v2.8.10) software.

### RNA-seq

RNA-seq was performed as described previously.^56^ In brief, naïve CD4^+^ T cells were polarized with IL-6 + IL-1β or IL-6 + IL-1β + IL-23. After 5 days of polarization, the cells were lysed with TRIzol Reagent (Invitrogen) to extract total RNA. Then 1 μg RNA /sample was prepared for mRNA library construction using the NEBNext Poly(A) mRNA Magnetic Isolation Module and the NEBNext Ultra II RNA Library Prep Kit for Illumina (New England Biolabs). The Qubit dsDNA Quantification Assay Kit (Thermo Scientific) was used for quality control of the libraries. Transcriptome sequencing was performed on the NovaSeq 6000 sequencer in accordance with the manufacturer’s instructions. The transcriptomic analysis was performed using R (4.0.3). The fastq files were mapped to the human GRCh38 genome assembly and transformed into an expression matrix. The limma R package was used to identify differentially expressed genes (DEGs) with *P* value < 0.05 and fold change > 2 according to the Benjamini–Hochberg method.

### ATAC-seq

ATAC-seq was performed according to the manufacturer’s instructions. In brief, nuclei were isolated from 50,000 viable cells by centrifugation at 500× *g* for 5 min. The supernatant was discarded, and the pellet was resuspended in 50 µL of lysis buffer and incubated on ice for 5 min. Subsequently, 1 mL of wash buffer was added, the mixture was homogenized, and nuclei were recovered by centrifugation at 500× *g* for 5 min. The tagmentation reaction was performed using the TruePrep DNA Library Prep Kit V2 for Illumina (Vazyme, Cat# TD501). The resulting DNA fragments were purified, and sequencing adapters were added to construct the final library. Library quality was assessed, and paired-end sequencing was performed on the Illumina platform.

### IP-MS

IP-MS was performed to identify proteins that interacted with RORγt and DNA-PKcs in Th17 cells. Total protein was extracted from polarized Th17 cells and incubated with anti-DNA-PKcs or anti-RORγt antibody overnight at 4 °C, and then incubated with protein A+G magnetic beads for 1 h the next morning. The beads were then washed twice with PBS containing 1 mM PMSF, and the samples were eluted. After dithiothreitol reduction and iodoacetamide alkylation, the samples were passed through 10-kDa ultrafiltration membranes and centrifuged at 12,000 rpm for 20 min, and 200 μL 50 mM ammonium bicarbonate solution was added. After enzymatic hydrolysis with 1 μg/μL trypsin at 37 °C for 12–18 h, the reaction was terminated by the addition of 10% trifluoroacetic acid. A desalting column was activated by mobile phase A (98% H_2_O + 2% ACN + 0.1% FA) and balanced by mobile phase B (98% ACN + 2% H_2_O + 0.1% FA). The samples were filtered through the desalting column and eluted with mobile phase B, and then dried with a vacuum centrifuge concentrator and dissolved in 15 μL mobile phase A. Then, 10 μL of each sample was subjected to MS. The spectra were collected from 350 to 1800 *m*/*z*, and the raw file was processed against the UniProtKB human database containing 20,404 proteins.

### IP-LC-MS/MS phospho-proteomics/proteomics

Peptides pulled down with DNA-PKcs were reduced with 5 mM dithiothreitol at 56 °C for 30 min and alkylated with 11 mM iodoacetamide at room temperature for 15 min. The sample was diluted with 100 mM TEAB to a urea concentration of less than 2 M. Finally, trypsin was supplemented with a 1:50 trypsin-to-protein mass ratio for the first digestion overnight and a 1:100 trypsin-to-protein mass ratio for a second 4 h-digestion, followed by desalting using a C18 SPE column. Phosphopeptides were enriched using a High-Select Fe-NTA Phosphopeptide Enrichment Kit and labeled with tandem mass tag reagents. The sample was first dissolved in solvent A (0.1% formic acid, 2% acetonitrile/in water) and then separated by a gradient of solvent B (6% to 24%) and analyzed by Orbitrap Fusion Lumos mass spectrometry (Thermo Fisher Scientific).

### scRNA-seq

PBMCs were washed three times with PBS and passed through a 40-μm cell strainer (Falcon) to obtain single-cell suspensions. The single PBMCs were counted and assessed for cell viability by trypan blue staining. Cell viability in these PBMC samples exceeded 95%. To construct barcoded scRNA-seq libraries, a Chromium single-cell controller (10× Genomics) was used to generate single-cell gel beads in emulsions (GEMs) from single-cell PBMC suspensions using a single-cell 5′ library and gel bead kit, a multiplex kit, and a chip kit (10× Genomics). Approximately 1000 cells/μL of single-cell PBMCs were loaded in each channel for capture and lysed to release the barcoded RNA in individual GEMs. Complementary DNA (cDNA) was synthesized by reverse transcription and amplified for 12 cycles. The cDNA was converted to an Illumina sequencing library and sequenced on the NovaSeq 6000 platform according to the manufacturer’s instructions. FastQC software was used to check the quality of each library. Generated baseline files (.bcl) were converted to fastq files using CellRanger (v5.1.0; 10× Genomics).

To generate scRNA-seq libraries for murine lymphocytes, dLNs were harvested from the necks of blank and EAU mice. The cells were ground in RPMI medium and passed through a 40-μm cell strainer to obtain single-cell suspensions. Barcoded scRNA-seq libraries were constructed as described above.

### scRNA-seq raw data processing

Filtered-output gene expression matrices were generated from each sample and merged using the count and aggregate commands, respectively, in Cell Ranger, according to the developers‘ instructions in the tutorial (https://support.10xgenomics.com/single-cell-gene-expression/software/pipelines/latest/using/count for count and https://support.10xgenomics.com/single-cell-gene-expression/software/pipelines/latest/using/aggregate for aggregate).^57^ R software (v4.0.3) was used to analyze the data with the R package Seurat (v4.0.1).^58^ Cells were excluded as low quality if they met any of the following criteria: (1) < 200 genes; (2) mitochondrial genome > 5%; (3) expression of RBC gene (*HBB*) > 5.00; and (4) expression of platelet gene (*PPBP*) > 5.00. The Seurat object was normalized using the NormalizeData function, and 1500 variable features were calculated using the FindVariableFeature function. Data were subjected to linear transformation using the ScaleData function and then to linear dimensional reduction using the RunPCA function with a dimensionality of 12. Finally, cells were clustered using the FindNeighbors and FindClusters functions and were subjected to nonlinear dimensional reduction using the RunTSNE function. The tutorial (https://satijalab.org/seurat/v3.0/pbmc3k_tutorial.html) for all Seurat analyzes performed in this study is available online.

### Identification of IRBP_1–20_-specific TCR sequence

Bone marrow-derived dendritic cells were stimulated with 20 μg/mL IRBP_1–20_ for 3 days. Then, the EAU-derived CTV-labeled CD44^+^ CD4^+^ T cells were co-cultured with the bone marrow-derived dendritic cells for another 3 days. The proliferating T cells were sorted from the co-culture system and were subjected to single-cell TCR sequencing (scTCR-seq). Enriched full-length TCRs were derived from amplified cDNA generated in 5′ libraries using a Chromium Single-Cell V(D)J Enrichment Kit (10x Genomics). TCR/BCR clonotype assignment was performed using Cell Ranger (v5.1.0, 10x Genomics) according to the developers’ instructions (https://support.10xgenomics.com/single-cell-vdj/software/pipelines/latest/using/vdj), after which TCR sequence information, clonotype frequency, and corresponding barcode information were generated. The most enriched TCR clonotype was listed in Supplementary information, Table S7 and was applied for the construction of IRBP1-20-specific TCR.

### Cell-type annotation

Cell clusters generated from the t-SNE dimensionality reduction were obtained using the Seurat FindAllMarker function to identify genes that could distinguish each cluster. Clusters were then classified and annotated according to specific gene markers for all major cell types: T cells (*CD3D*, *CD3E*, and *CD3G*), myeloid cells (*LYZ*, *S100A9*, and *CST3*), NK cells (*KLRF1*, *GZMB*, and *NKG7*), and B cells (*CD79A*, *MS4A1*, *CD79B*, and *BANK1*) (see Supplementary information, Fig. S10a, b). Clusters that expressed two or more gene markers were considered multiplets and were excluded from the data. Cells that expressed *RORC* (encoding RORγt) were considered primary Th17 cells. These cells also expressed Th17 hallmark genes, including *CCR6* and *KLRB1* (see Supplementary information, Fig. S10). To identify the cell type of each Th cell in the murine scRNA-seq data, memory CD4^+^ T cells were identified as *Cd4*^+^*Cd44*^−^*Ccr7*^−^, then sub-classified as Th1 (*Ifng*^+^), Th2 (*Il4*^+^), Treg (*Foxp3*^+^), Tfh (*Tox2*^+^), Th17 (*Rorc*^+^), pTh17 (*Rorc*^+^, *Cxcr6*^+^) and nTh17 (*Rorc*^+^, *Cxcr6*^−^, *Tcf7*^med^).

### Pseudotime trajectory construction

To analyze the transcriptional dynamics of Th17 cells, the Monocle 3 (v1.0.0) R package was used to identify cell differentiation trajectories, and the beginning of the trajectory was defined as cells in the HC group.

### DEG identification

The R package limma (v3.44.3) was used to identify DEGs between two groups. *P* values were adjusted using the Benjamini–Hochberg method. Transcripts with a logarithmic fold change greater than 0.5 between two groups and a *P* value < 0.05 were considered significant. DEGs were displayed in the form of a heatmap, volcano plot, or lollipop chart. For multigroup comparison, the R package Monocle 3 (v1.0.0) was used to identify significantly overexpressed genes in each group using the “top_markers” function, and transcripts with *P* values < 0.05 were considered significant.

### GSVA

GSVA was used to estimate the variation in pathway activity of individual cells from each group of patients with AU on the basis of a particular molecular signature database.^59^ Cellular functions were analyzed using the R package GSVA (v1.36.3) in terms of hallmarks, KEGG pathways, and GO annotations. Thresholds were set at an enrichment score change > 0.5 and a *P* value < 0.05.^60^

### Cell–cell interaction analysis

To explore potential interactions among major cell types in the PBMCs from each group, cell-cell interaction analysis was performed using the Python package CellPhoneDB (v1.1.0). The single-cell expression matrices in each group were prepared for the analysis based on the receptors and ligands between the two cell types.^61^ The information on the analyzed cells was listed as barcodes and annotated cell types. Then, the matrix and annotated cell type information were used as input for the analysis, which calculated the theoretical receptor-ligand interaction score and *P* value between two cell types.

### Statistics

All data were collected and analyzed using the devices and software listed in Supplementary information, Tables S9 and S10. Statistical analyses were performed with Prism 9 software (GraphPad). Normally distributed data are displayed as mean ± standard deviation (SD) with individual values. The statistical significance between two groups was determined by unpaired or paired Student’s *t*-tests. Multiple groups were compared by one-way analysis of variance followed by Tukey’s test. For multiple comparisons for a mixed model with more than two factors, two-way analysis of variance followed by Bonferroni’s test was used. Non-parametric data are shown as individual values with the median. Multiple comparisons of non-parametric data were performed by one-way analysis of variance followed by Dunn’s test. Pearson’s correlation was used to analyze the correlation between two variables. A *P* value < 0.05 was considered to be statistically significant. Numbers of replicates are indicated in each figure legend. For in vitro studies, each symbol represents one individual sample from a human donor, and each individual sample was not used again within the group. Each individual experiment was performed at a different time. For in vivo studies, each symbol indicates one experimental animal. As noted in the figure legends, most of the in vivo data were combined from at least two rounds of independent experiments with at least three biological replicates per round.

## Supplementary information

Supplementary information, Fig. S1

Supplementary information, Fig. S2

Supplementary information, Fig. S3

Supplementary information, Fig. S4

Supplementary information, Fig. S5

Supplementary information, Fig. S6

Supplementary information, Fig. S7

Supplementary information, Fig. S8

Supplementary information, Fig. S9

Supplementary information, Fig. S10

Supplementary information, Fig. S11

Supplementary information, Fig. S12

Supplementary information, Fig. S13

Supplementary information, Table S1

Supplementary information, Table S2

Supplementary information, Table S3

Supplementary information, Table S4

Supplementary information, Table S5

Supplementary information, Table S6

Supplementary information, Table S7

Supplementary information, Table S8

Supplementary information, Table S9

Supplementary information, Table S10

Supplementary information, Table S11

## Data Availability

scRNA-seq, RNA-seq, and ChIP-seq data generated in this study have been deposited at the Genome Sequence Archive with the following accession numbers: CRA008977, HRA006260, HRA012642, HRA006243, HRA001580, CRA016165, and HRA013725 (Supplementary information, Table S11).

## Code availability

No custom-made code was used in this study. The pipelines for analysis can be obtained by e-mailing the lead contact.
