# Supplementary material for: Sensing of DNA double-strand breaks by the NHEJ system stabilizes RORγt transcriptional activity and shapes Th17 pathogenicity in autoimmunity
Source: Cell Res. 2026 Jan 7;36(5):340–58. doi: 10.1038/s41422-025-01204-6 (PMC13092643; doi:10.1038/s41422-025-01204-6)
Supplement: Supplementary file 1 — Supplementary information, Fig. S1 [file 41422_2025_1204_MOESM1_ESM.pdf]

Figure S1 (Related to Figure 1)

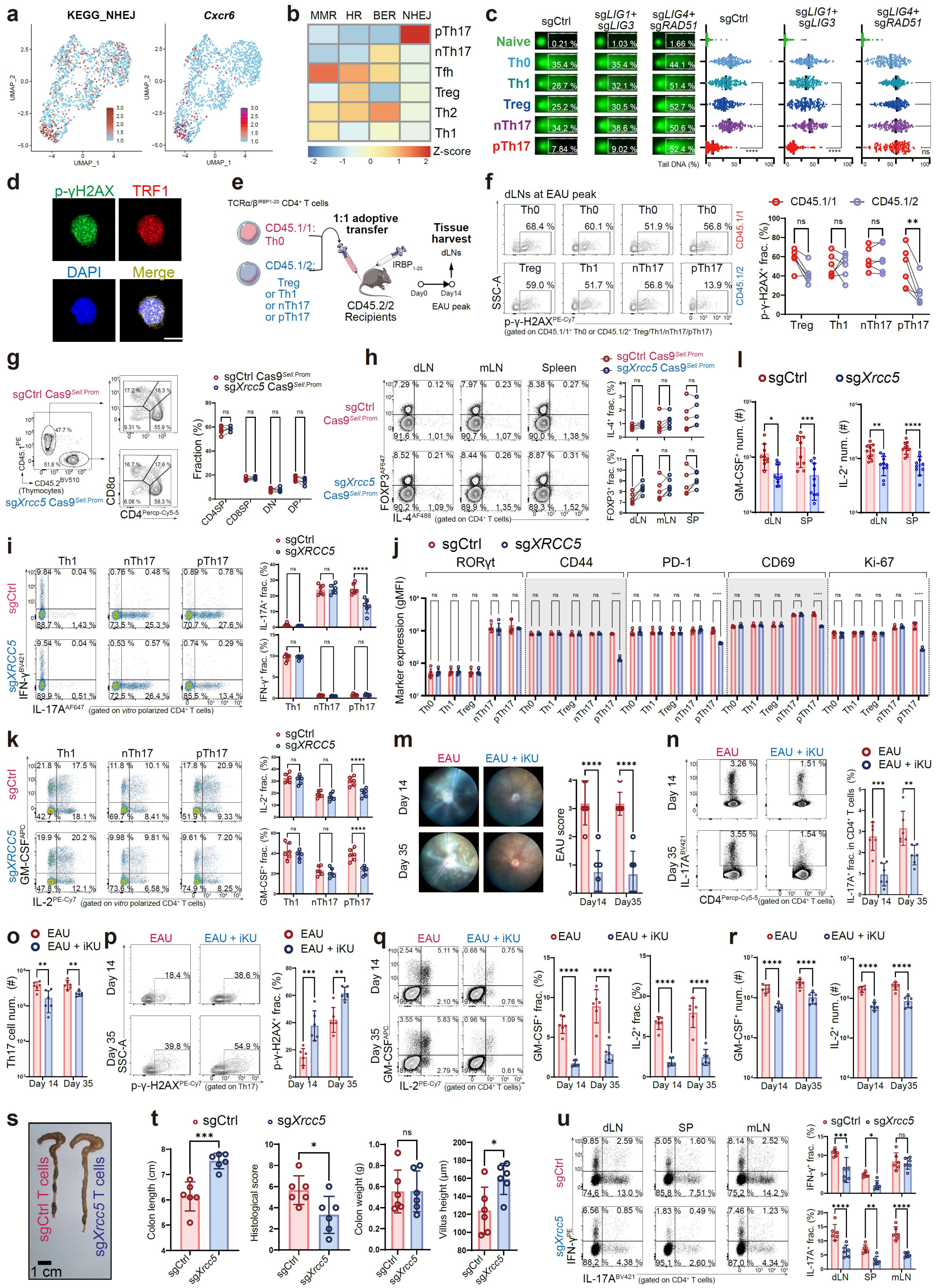

Fig. S1. Ablation of DSB-sensing by NHEJ system inhibits Th17 response in autoimmunity. Related to Figure 1.

- **a.** UMAP plots showing the correlation in cell-distribution between NHEJ score calculated by GSVA analysis and *Cxcr6* gene expression (pTh17 hallmark) in murine scRNA-seq data of EAU mice.
- **b.** Heatmap showing the functional activity of each DNA repair pathway scored by GSVA analysis of murine scRNA-seq data of EAU mice.
- **c.** Neutral comet assay for measuring DSB-level among each T effector subset with knock-out of *LIG1* + *LIG3*, or *LIG4* + *RAD51*. The experiment was repeated 3 times and 50 single-cells were analyzed within each time (n = 150).
- **d.** Immunofluorescence assay to detect the co-localization of TRF1 (red) and p-γ-H2AX (green) in human *LIG4*^{KO} pTh17 cells (scale bar = 10 μm).
- **e.** Experimental scheme showing adoptive transfer of each subset of T cells with CD45.1/1^{+} Th0 control cells and CD45.1/2^{+} polarized Treg, Th1, nTh17 and pTh17 cells to CD45.2/2^{+} EAU recipients. Data was combined from 2 independent experiments with n = 6.
- **f.** Flow cytometric (FC) analysis gated on CD45.1/1^{+} or CD45.1/2^{+} cells examining DSB formation across each Th subset of transferred cells marked by p-γ-H2AX^{+} (n = 6).
- **g.** FC analysis gated on chimeric thymocytes showing the thymic development of T cells in BM-chimeric mice (n = 6).
- **h.** FC analysis gated on CD4^{+} T cells showing the fraction of Th2 and Treg in chimera mice reconstituted with mixed BM (n = 6).
- **i.** FC analysis showing the secretion of IFN-γ and IL-17A in sg*XRCC5* human T cells after 5-day-induction towards Th1, nTh17 or pTh17 (n = 6).
- **j.** FC analysis of activation marker (RORγt, CD44, PD-1, CD69 and Ki-67) expression on human *XRCC5*-deficient T cells which were cultured under Th0, Th1, Treg, nTh17 or pTh17 differentiation status for 5 days (n = 6).
- **k.** FC analysis showing the secretion of IL-2 and GM-CSF in sg*XRCC5* human T cells after 5-day-induction towards Th1, nTh17 or pTh17 (n = 6).
- **l.** Statistical graphs for the cell number of the CD4^{+} T cells producing GM-CSF and IL-2 in lymph organ of EAU *Rag1*$^{-/-}$ mice transferred with sgCtrl or sg*Xrcc5* pTh17 (n = 10).
- **m.** Fundoscopic graphs showing the ocular fundus of EAU mice treated with 25 mg/kg/day STL127705 (iKU) from day 7 to day 14 or day 35 and the related statistical graph. Data was combined from 2 independent experiments with n = 6.
- **n.** FC analysis gated on CD4^{+} T cells showing Th17 fraction in dLNs of EAU mice treated with STL127705 (n = 6).
- **o.** Statistical chart showing the number of Th17 cells of (n).
- **p.** FC analysis showing levels of DSB-accumulation gated on Th17 cells in (n) (n = 6).
- **q.** FC analysis gated on CD4^{+} T cells showing the fraction of cells secreting IL-2 and GM-CSF in dLNs of EAU mice treated with STL127705 (n = 6).
- **r.** Statistical charts showing the number of CD4^{+} T cells producing IL-2 and

GM-CSF cells of (q).

s. Representative macroscopic pictures of colon, collected at 8 weeks after transfer of $CD25^-$ $CD45RB^{hi}$ naïve T cells (n = 6).

t. Statistical graphs examining the effect of adoptively transferring sgCtrl or sg*Xrcc5* $CD25^-$ $CD45RB^{hi}$ naïve T cells on the colon length, histological pathology, colon weight and villus height of *Rag1*$^{-/-}$ recipients, the data of which were collected at 8 weeks after transfer of $CD25^-$ $CD45RB^{hi}$ naïve T cells (n = 6).

u. FC analysis displaying the fraction of Th1 and Th17 in lymph organ of *Rag1*$^{-/-}$ recipients transferred with sgCtrl or sg*Xrcc5* $CD25^-$ $CD45RB^{hi}$ naïve T cells after 8 weeks (n = 6).

Statistics were calculated by paired or unpaired Student's t test or two-way analysis of variance followed by Bonferroni's test. Error bars represent mean ± SD. *$P < 0.05$; **$P < 0.01$, ***$P < 0.001$, ****$P < 0.0001$.
