# Supplementary material for: Sensing of DNA double-strand breaks by the NHEJ system stabilizes RORγt transcriptional activity and shapes Th17 pathogenicity in autoimmunity
Source: Cell Res. 2026 Jan 7;36(5):340–58. doi: 10.1038/s41422-025-01204-6 (PMC13092643; doi:10.1038/s41422-025-01204-6)
Supplement: Supplementary file 2 — Supplementary information, Fig. S2 [file 41422_2025_1204_MOESM2_ESM.pdf]

Figure S2 (Related to Figure 1)

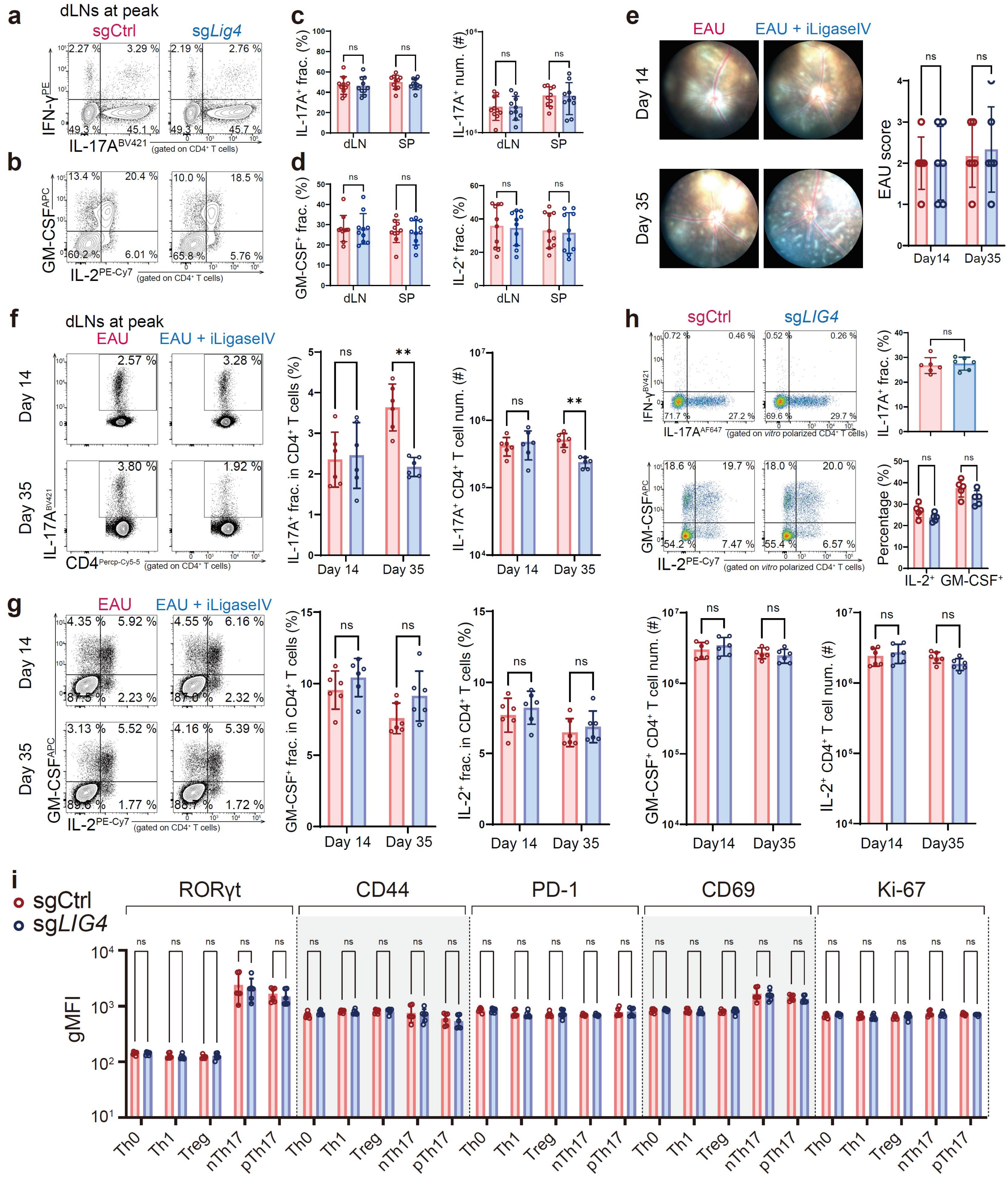

Fig. S2. Effector function of Th17 cells is not required with DSB-ligation. Related to Figure 1.

a. FC analysis gated on $CD4^+$ T cells showing the fraction and number of IL-$17A^+$ cells in lymph organ of EAU *Rag1*$^{-/-}$ mice transferred with sgCtrl or sg*Lig4* pTh17 (n = 10).

b. FC analysis gated on $CD4^+$ T cells showing the fraction of GM-CSF^+ and IL-2^+ cells in lymph organ of EAU *Rag1*$^{-/-}$ mice transferred with sgCtrl or sg *Lig4* pTh17 (n = 10).

c. Statistical graphs for the fraction and number of the $CD4^+$ T cells producing IL-17A in lymph organ of EAU *Rag1*$^{-/-}$ mice transferred with sgCtrl or sg*Lig4* pTh17 (n = 10).

d. Statistical graphs for the fraction and number of the $CD4^+$ T cells producing GM-CSF and IL-2 in lymph organ of EAU *Rag1*$^{-/-}$ mice transferred with sgCtrl or sg*Lig4* pTh17 (n = 10).

e. Fundoscopic graphs showing the ocular fundus of EAU mice treated with 25 mg/kg/day SCR130 (iLigaseIV) from day 7 to day 14 or day 35 and the related statistical graph. Data was combined from 2 independent experiments with n = 6.

f. FC analysis gated on $CD4^+$ T cells showing the fraction and number of Th17 in dLNs of EAU mice treated with SCR130 (n = 6).

g. FC analysis gated on $CD4^+$ T cells showing the fraction of cells secreting IL-2 and GM-CSF in dLNs of EAU mice treated with SCR130 (n = 6).

h. FC analysis showing the secretion of IL-17A, IL-2 and GM-CSF in sg*LIG4* human T cells after 5-day-induction towards pTh17 (n = 6).

i. FC analysis of activation marker (RORγt, CD44, PD-1, CD69 and Ki-67) expression on human *LIG4*-deficient T cells which were cultured under Th0, Th1, Treg, nTh17 or pTh17 differentiation status for 5 days (n = 6).

Statistics were calculated by unpaired Student’s t test or two-way analysis of variance followed by Bonferroni’s test. Error bars represent mean ± SD. $^{**}P < 0.01$.
