# Supplementary material for: Sensing of DNA double-strand breaks by the NHEJ system stabilizes RORγt transcriptional activity and shapes Th17 pathogenicity in autoimmunity
Source: Cell Res. 2026 Jan 7;36(5):340–58. doi: 10.1038/s41422-025-01204-6 (PMC13092643; doi:10.1038/s41422-025-01204-6)
Supplement: Supplementary file 3 — Supplementary information, Fig. S3 [file 41422_2025_1204_MOESM3_ESM.pdf]

Figure S3 (Related to Figure 1)

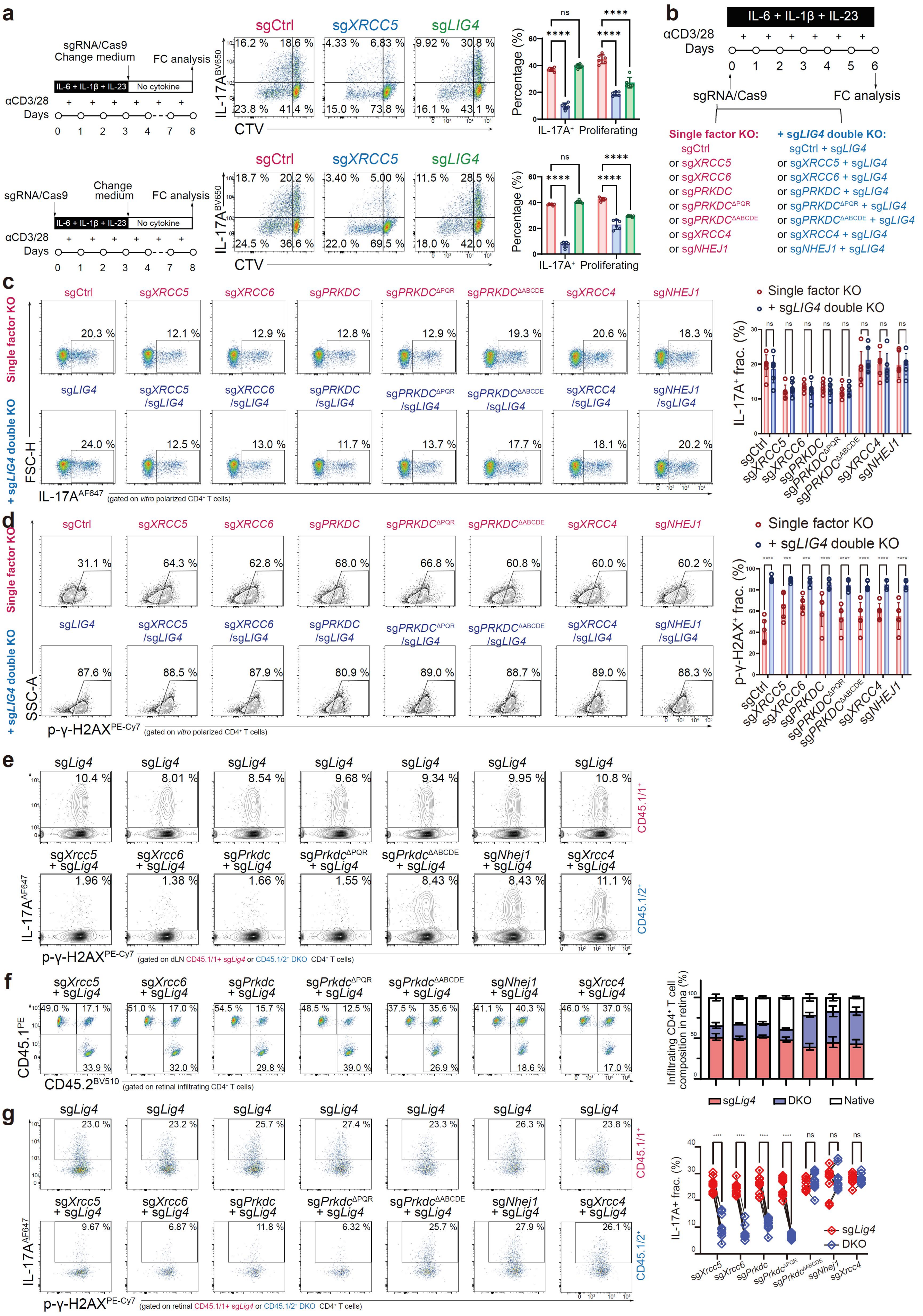

Fig. S3. Effector function of Th17 cells is relied on KU-dependent DSB-sensing. Related to Figure 1.

a. FC analysis showing the proliferation index and IL-17A secretion of sg*XRCC5* or sg*LIG4* T cells labelled by CellTrace Violet (CTV) after 5-day-indction towards pTh17 (n = 6).

b. Experimental scheme to examine the effect of double knock-out of both *LIG4* and each NHEJ factor on the differentiation of *in vitro* polarized human pTh17 cells (n = 6).

c. FC analysis showing the secretion of IL-17A in cells of (b) (n = 6).

d. FC analysis to examine the effect of double knock-out of both *LIG4* and each NHEJ factor on the levels of DSB-accumulation following the differentiation towards pTh17 cells (n = 6).

e. Representative graphs for FC analysis showing the secretion of IL-17A in the transferred CD45.1/1$^+$ sg*Lig4* cells and CD45.1/2$^+$ cells with double knock-out of both *Lig4* and each NHEJ factor in Fig. 1q (n = 10).

f. Representative and statistical graphs for FC analysis showing the composition of CD45.2/2$^+$ WT native CD4$^+$ T cells, CD45.1/1$^+$ sgCtrl pTh17 and CD45.1/2$^+$ pTh17 with knock-out of each NHEJ factor in retina of CD45.2/2$^+$ EAU mice (n = 10).

g. Representative and statistical graphs for FC analysis showing the secretion of IL-17A in CD45.1/1$^+$ sgCtrl pTh17 and CD45.1/2$^+$ pTh17 with knock-out of each NHEJ factor in retina of CD45.2/2$^+$ EAU mice (n = 10).

Statistics were calculated by paired or unpaired Student's t test or one-way analysis of variance followed by Turkey test or two-way analysis of variance followed by Bonferroni's test. Error bars represent mean ± SD. **P* < 0.05; ***P* < 0.01, ****P* < 0.001, *****P* < 0.0001.
