# Supplementary material for: Sensing of DNA double-strand breaks by the NHEJ system stabilizes RORγt transcriptional activity and shapes Th17 pathogenicity in autoimmunity
Source: Cell Res. 2026 Jan 7;36(5):340–58. doi: 10.1038/s41422-025-01204-6 (PMC13092643; doi:10.1038/s41422-025-01204-6)
Supplement: Supplementary file 4 — Supplementary information, Fig. S4 [file 41422_2025_1204_MOESM4_ESM.pdf]

Figure S4 (Related to Figure 2)

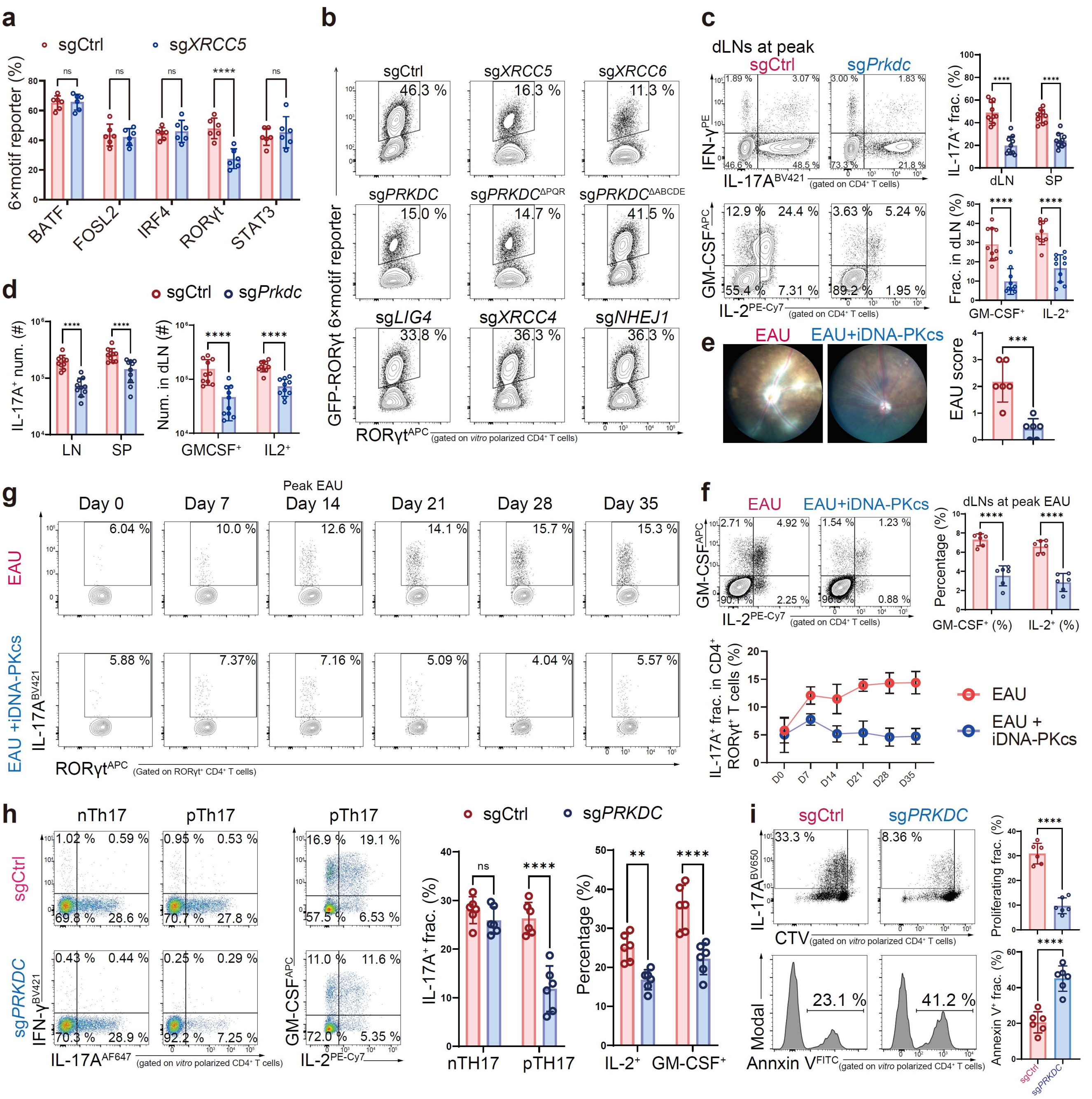

Fig. S4. DNA-PKcs supports Th17 pathogenicity by sustaining RORγt transcriptional activity. Related to Figure 2.

a. Statistical graph for analyzing the transcriptional activity of Th17-related TF in sgCtrl or sg*XRCC5* pTh17 using 6×motif reporter system. Human naïve $CD4^+$ T cells were co-transduced with 6×motif reporter system and sgRNA/Cas9 system, followed by polarization at pTh17 condition for 5 days. The expression of reporter was measured by flow-cytometry.

b. FC analysis showing the effect of knock-out of each NHEJ factor on the expression of RORγt reporter following pTh17 differentiation (n = 3).

c. FC analysis gated on $CD4^+$ T cells showing the fraction of IL-$17A^+$, $IL2^+$ and GM-CSF^+ cells in lymph organ of EAU *Rag1*$^{-/-}$ mice transferred with sgCtrl or sg*Prkdc* pTh17 (n = 10).

d. Statistical graphs for the cell number of the $CD4^+$ T cells producing IL-17A, GM-CSF and IL-2 in lymph organ of the EAU *Rag1*$^{-/-}$ mice transferred with sgCtrl or sg*Prkdc* pTh17 (n = 10).

e. Fundoscopic graphs showing the ocular fundus of EAU mice treated with 10 mg/kg/day NU7441 (iDNA-PKcs) from day 7 to day 14 and the related statistical graph. Data was combined from 2 independent experiments with n = 6.

f. FC analysis gated on $CD4^+$ T cells showing the fraction of cells secreting IL-2 and GM-CSF in dLNs of EAU mice treated with iDNA-PKcs from day 7 to day 14 (n = 6).

g. FC analysis gated on $RORγt^+$ $CD4^+$ T cells showing the fraction of IL-$17A^+$ cells in dLNs of the EAU mice consecutively treated with 10 mg/kg/day NU7441 for 0, 7, 14, 21, 28 or 35 days (n = 4 of each time point of the treating course).

h. FC analysis showing the secretion of IL-17A, IL-2 and GM-CSF in human sg*PRKDC* T cells after 5-day-induction towards nTh17or pTh17 (n = 6).

i. FC analysis to examine the role of DNA-PKcs in maintaining Th17 expansion using T cell proliferation and apoptosis assay. Briefly, polarized pTh17 cells labeled with CTV were transduced with sg*PRKDC*/Cas9 system and were kept culture under pTh17 condition for 3 days (n = 6). The proliferating Th17 were measured by the proliferating waves and apoptotic Th17 were shown as Annexin V^+.

Statistics were calculated by unpaired Student's t test or one-way analysis of variance followed by Turkey test or two-way analysis of variance followed by Bonferroni's test. Error bars represent mean ± SD. **P* < 0.05; ***P* < 0.01, ****P* < 0.001, *****P* < 0.0001.
