# Supplementary material for: Sensing of DNA double-strand breaks by the NHEJ system stabilizes RORγt transcriptional activity and shapes Th17 pathogenicity in autoimmunity
Source: Cell Res. 2026 Jan 7;36(5):340–58. doi: 10.1038/s41422-025-01204-6 (PMC13092643; doi:10.1038/s41422-025-01204-6)
Supplement: Supplementary file 5 — Supplementary information, Fig. S5 [file 41422_2025_1204_MOESM5_ESM.pdf]

Figure S5 (Related to Figure 3)

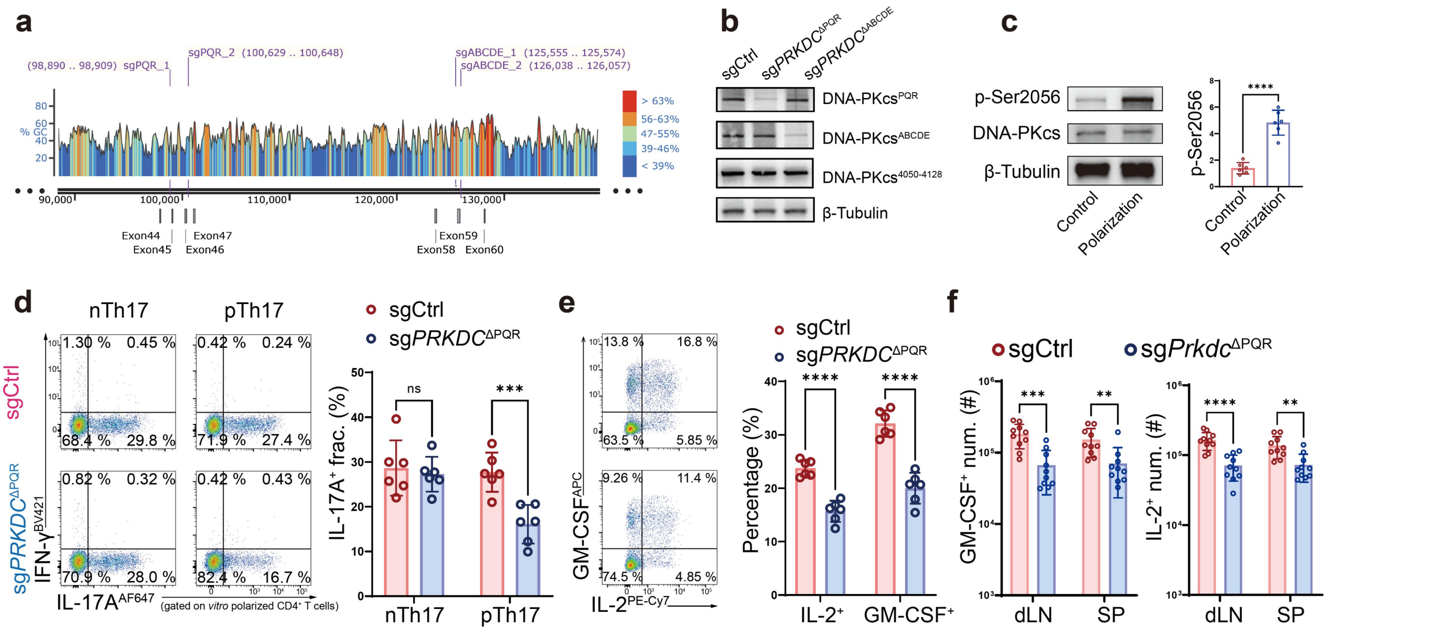

Fig. S5. Depleting PQR cluster in DNA-PKcs weakens Th17 pathogenicity. Related to Figure 3.

a. Scheme showing the design of sgRNA targeting the flanking introns of the exons encoding PQR or ABCDE cluster within the ORF of *PRKDC* gene loci.

b. Western blot analysis of whole-cell lysates using the antibodies targeting PQR cluster or ABCDE cluster from the sgCtrl pTh17 cells or the cells carrying sg*PRKDC*$^{\Delta PQR}$ or *sgPRKDC*$^{\Delta ABCDE}$ CRISPR system in (**a**) with the application of HDR donors (n = 4).

c. Immunoblot for analyzing the auto-phosphorylation at PQR cluster of DNA-PKCs in human naïve CD4$^+$ T cells or polarized pTh17 cells (n = 6).

d. FC analysis showing the secretion of IL-17A in sg*PRKDC*$^{\Delta PQR}$ T cells after 5-day-induction towards nTh17 or pTh17 (n = 6).

e. FC analysis showing the secretion of IL-2 and GM-CSF in sg*PRKDC*$^{\Delta PQR}$ T cells after 5-day-induction towards nTh17 or pTh17 (n = 6).

f. Statistical graphs for the cell number of the CD4$^+$ T cells producing GM-CSF and IL-2 in lymph organ of EAU *Rag1*$^{-/-}$ mice transferred with sgCtrl or sg*Prkdc*$^{\Delta PQR}$ pTh17 (n = 10).

Statistics were calculated by unpaired Student's t test or one-way analysis of variance followed by Turkey test. Error bars represent mean ± SD. *$P < 0.05$; **$P < 0.01$, ***$P < 0.001$, ****$P < 0.0001$.
