# Supplementary material for: Sensing of DNA double-strand breaks by the NHEJ system stabilizes RORγt transcriptional activity and shapes Th17 pathogenicity in autoimmunity
Source: Cell Res. 2026 Jan 7;36(5):340–58. doi: 10.1038/s41422-025-01204-6 (PMC13092643; doi:10.1038/s41422-025-01204-6)
Supplement: Supplementary file 6 — Supplementary information, Fig. S6 [file 41422_2025_1204_MOESM6_ESM.pdf]

# Figure S6 (Related to Figure 4)

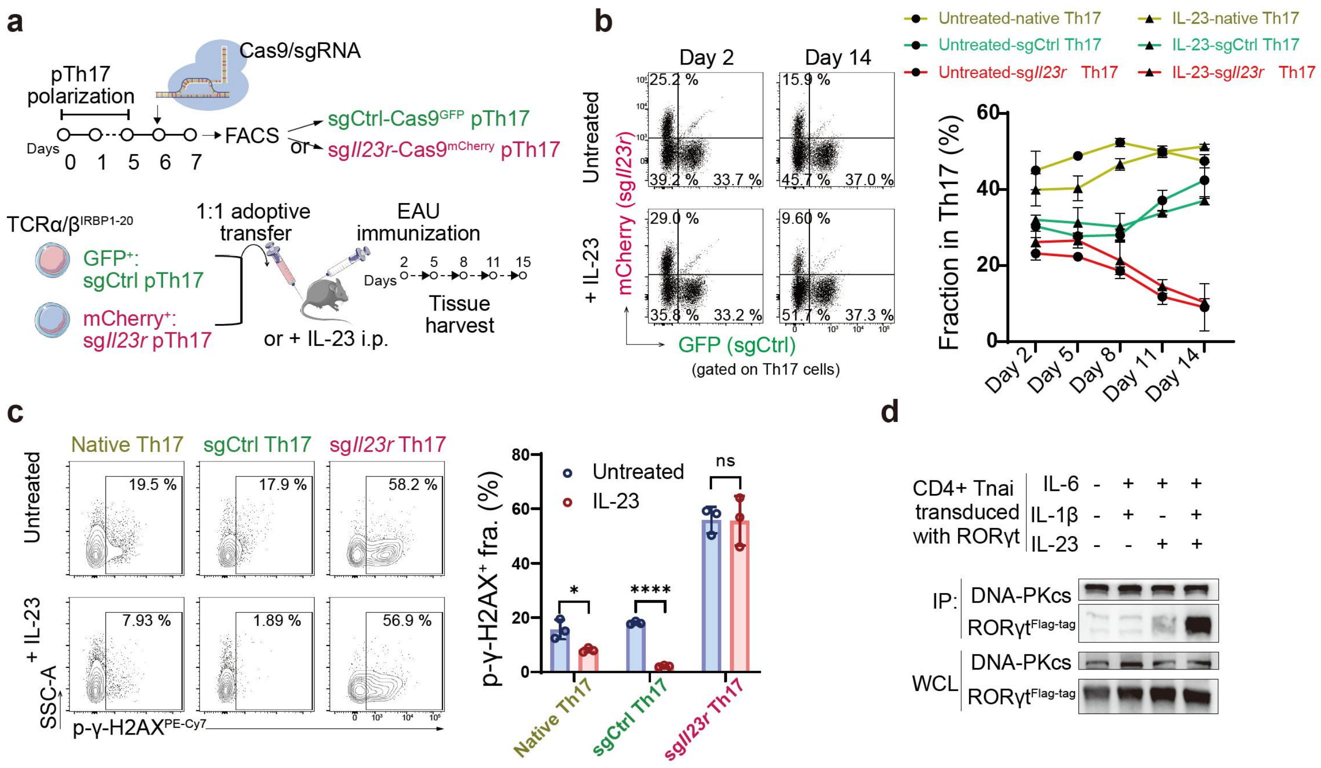

**Fig. S6. IL-23R signaling sustains NHEJ process in Th17 cells. Related to Figure 4.**

**a.** Experimental scheme showing the adoptive transfer of sgCtrl-Cas9$^{GFP}$ pTh17s or sg*Il23r*- Cas9$^{mCherry}$ pTh17 into EAU recipients with or without recombinant 10 μg/kg/day IL-23 treatment.

**b.** FC analysis showing the composition of Th17 in dLNs from the EAU recipients that received sgCtrl-Cas9$^{GFP}$ pTh17s and sg*Il23r*- Cas9$^{mCherry}$ pTh17 cells at day 2, 5, 8, 11 and 14 (n = 3 each time point).

**c.** FC analysis showing the level of DSB-accumulation in native and transferred pTh17 cells (n = 3 of each time point).

**d.** Co-IP assay showing the interaction of DNA-PKcs and RORγt in human T cells over-expressed with RORγt at indicated cytokine stimulation (n = 3)

Statistics were calculated by one-way analysis of variance followed by Turkey test or two-way analysis of variance followed by Bonferroni's test. Error bars represent mean ± SD. **P* < 0.05; ***P* < 0.01, ****P* < 0.001, *****P* < 0.0001.
