# Supplementary material for: Sensing of DNA double-strand breaks by the NHEJ system stabilizes RORγt transcriptional activity and shapes Th17 pathogenicity in autoimmunity
Source: Cell Res. 2026 Jan 7;36(5):340–58. doi: 10.1038/s41422-025-01204-6 (PMC13092643; doi:10.1038/s41422-025-01204-6)
Supplement: Supplementary file 7 — Supplementary information, Fig. S7 [file 41422_2025_1204_MOESM7_ESM.pdf]

Figure S7 (Related to Figure 5)

a

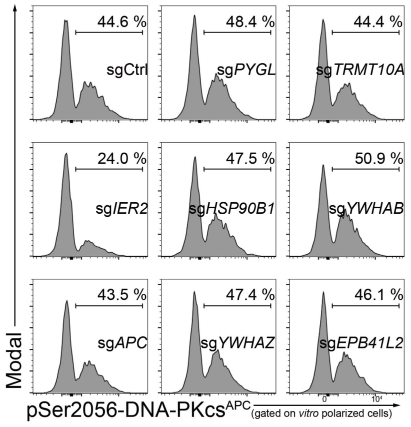

b

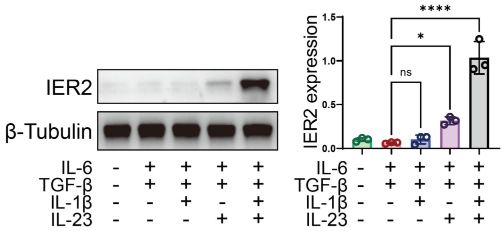

c

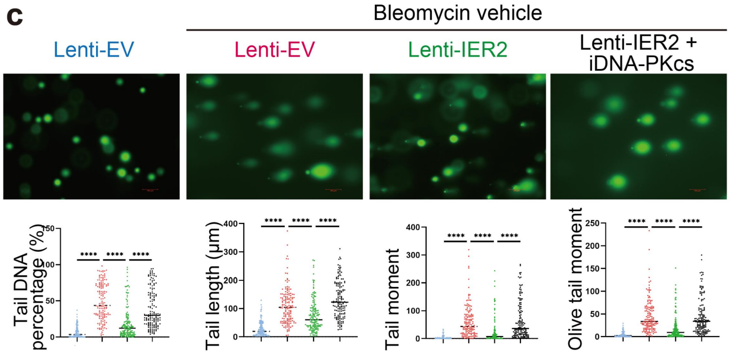

Fig. S7. IL-23 stimulates the expression of IER2 to sustain NHEJ activity. Related to Figure 5.

a. FC analysis showing the auto-phosphorylation at S2056 in human polarized pTh17 cells introduced with sgRNA/Cas9 system targeting the red-labelled genes in Fig. 5d (n = 3).

b. Immunoblot for analyzing the expression of IER2 in $CD4^+$ T cells polarized with indicated cytokines (n = 3).

c. Representative images and statistical graphs of comet assay showing the extent of DNA damage in human polarized pTh17 cells that were transduced with IER2 over-expression vector or 1 μM NU7441 (iDNA-PKcs) treatment (scale bar: 100 μm). The experiment was repeated 3 times and 50 single-cells were analyzed within each time (n = 150).

Statistics were calculated by unpaired Student's t test or one-way analysis of variance followed by Turkey test. Error bars represent mean ± SD. *$P < 0.05$; **$P < 0.01$, ***$P < 0.001$, ****$P < 0.0001$.
