# Supplementary material for: Sensing of DNA double-strand breaks by the NHEJ system stabilizes RORγt transcriptional activity and shapes Th17 pathogenicity in autoimmunity
Source: Cell Res. 2026 Jan 7;36(5):340–58. doi: 10.1038/s41422-025-01204-6 (PMC13092643; doi:10.1038/s41422-025-01204-6)
Supplement: Supplementary file 8 — Supplementary information, Fig. S8 [file 41422_2025_1204_MOESM8_ESM.pdf]

# Figure S8 (Related to Figure 6)

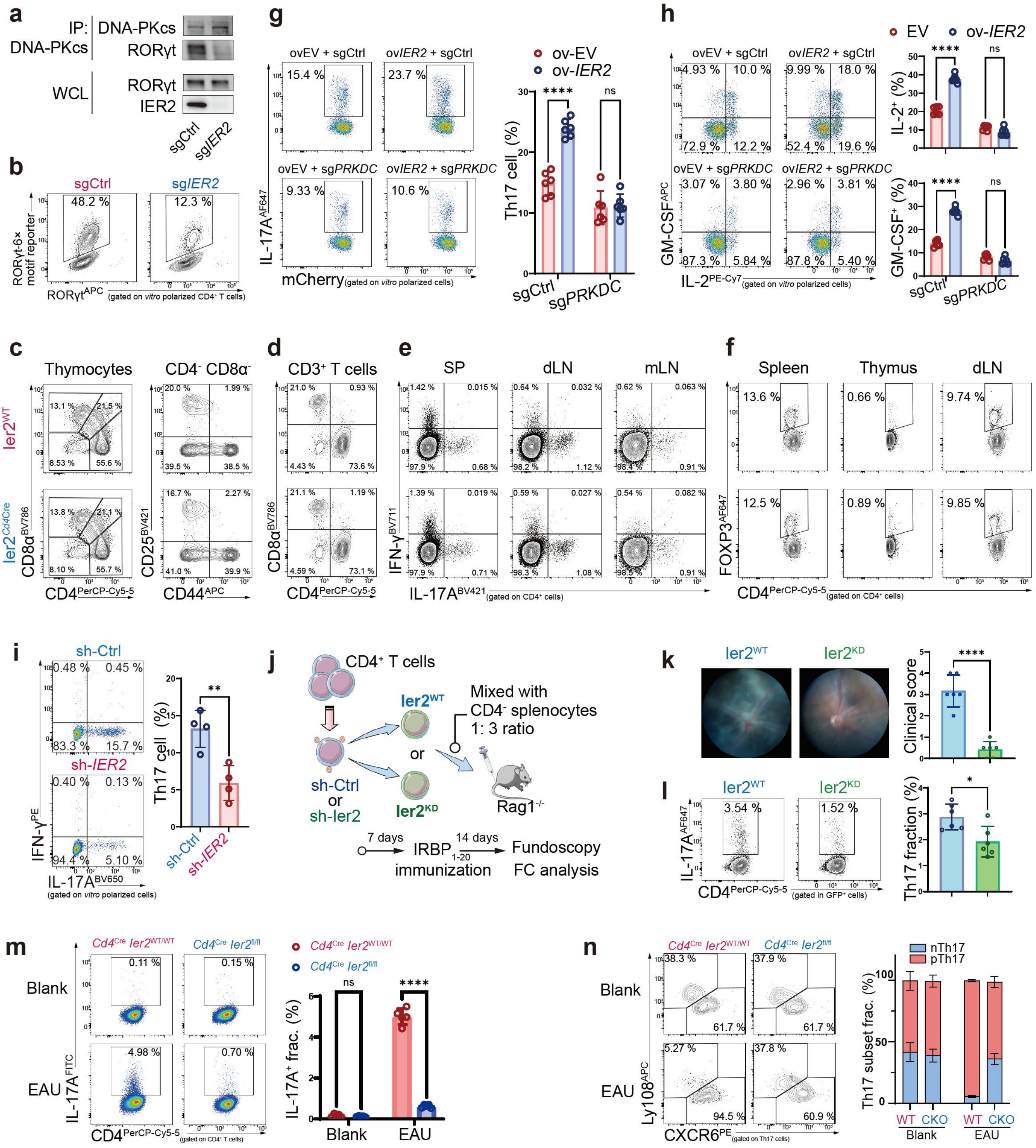

**Fig. S8. IER2 is required for Th17 effector function. Related to Figure 6.**

**a.** Immunoblot for Co-IP assay showing the interaction of DNA-PKcs and RORγt in polarized human pTh17 transduced with CRISPR system targeting on *IER2* (n = 3).

**b.** FC analysis showing the expression RORγt reporter in pTh17 polarized from sgCtrl or sg*IER2* human naïve T cells (n = 6).

**c.** FC analysis gated on $CD3^+$ T cells showing the thymic development of T cells in $Cd4^{Cre}$ $Ier2^{WT}$ ($Ier2^{WT}$) or $Cd4^{Cre}$ $Ier2^{fl/fl}$ ($Ier2^{Cd4Cre}$) mice. Data was combined from 2 independent experiments with n = 6.

**d.** FC analysis gated on $CD3^+$ T cells showing the fraction of $CD4^+$ and $CD8^+$ T cells in dLNs of untreated $Cd4^{Cre}$ $Ier2^{WT}$ or $Cd4^{Cre}$ $Ier2^{fl/fl}$ mice. Data was combined from 2 independent experiments with n = 7.

**e.** FC analysis gated on $CD4^+$ T cells showing the fraction of Th1 and Th17 cells in lymph organs of untreated $Cd4^{Cre}$ $Ier2^{WT}$ or $Cd4^{Cre}$ $Ier2^{fl/fl}$ mice (n = 7).

**f.** FC analysis gated on $CD4^+$ T cells showing the fraction of Treg cells in lymph organs of untreated $Cd4^{Cre}$ $Ier2^{WT}$ or $Cd4^{Cre}$ $Ier2^{fl/fl}$ mice (n = 7).

**g.** FC analysis showing the differentiation of pTh17 after 5-day-induction from murine naïve $CD4^+$ T cells transduced with indicated vectors (n = 6).

**h.** FC analysis showing the secretion of GM-CSF and IL-2 in polarized pTh17 cells after 5-day-induction from naïve $CD4^+$ T cells transduced with indicated vectors (n = 6).

**i.** FC analysis showing the differentiation of pTh17 after 5-day-induction from naïve $CD4^+$ T cells transduced with shRNA-*IER2* (n = 4).

**j.** Experimental scheme showing the transfer of $1 \times 10^6$/mouse $CD4^+$ T cells with or without *Ier2* knocking down into $Rag1^{-/-}$ recipient mice. The mice were then immunized with $IRBP_{1-20}$ to induce EAU model (*n* = 6).

**k.** Representative photographs and statistical graph of fundoscopic examination in $Rag1^{-/-}$ recipient mice at day 21 after EAU establishment (*n* = 6).

**l.** FC analysis gated on $CD4^+$ T cells showing the fraction of Th17 in dLNs from the $Rag1^{-/-}$ recipients (*n* = 6).

**m.** FC analysis showing the fraction of Th17 in dLNs in $Cd4^{Cre}$ $Ier2^{WT}$ and $Cd4^{Cre}$ $Ier2^{fl/fl}$ mice with or without EAU model establishment (n = 5).

**n.** FC analysis showing the fraction of pTh17 ($CXCR6^+$ $Ly108^-$) and nTh17 ($CXCR6^-$ $Ly108^+$) in $Cd4^{Cre}$ $Ier2^{WT}$ and $Cd4^{Cre}$ $Ier2^{fl/fl}$ mice with or without EAU model establishment (n = 5).

Statistics were calculated by unpaired Student's t test or unpaired two-way analysis of variance followed by Bonferroni's test. Error bars represent mean ± SD. *$P < 0.05$; **$P < 0.01$, ***$P < 0.001$, ****$P < 0.0001$.
