# Supplementary material for: Sensing of DNA double-strand breaks by the NHEJ system stabilizes RORγt transcriptional activity and shapes Th17 pathogenicity in autoimmunity
Source: Cell Res. 2026 Jan 7;36(5):340–58. doi: 10.1038/s41422-025-01204-6 (PMC13092643; doi:10.1038/s41422-025-01204-6)
Supplement: Supplementary file 9 — Supplementary information, Fig. S9 [file 41422_2025_1204_MOESM9_ESM.pdf]

Figure S9 (Related to Figure 7)

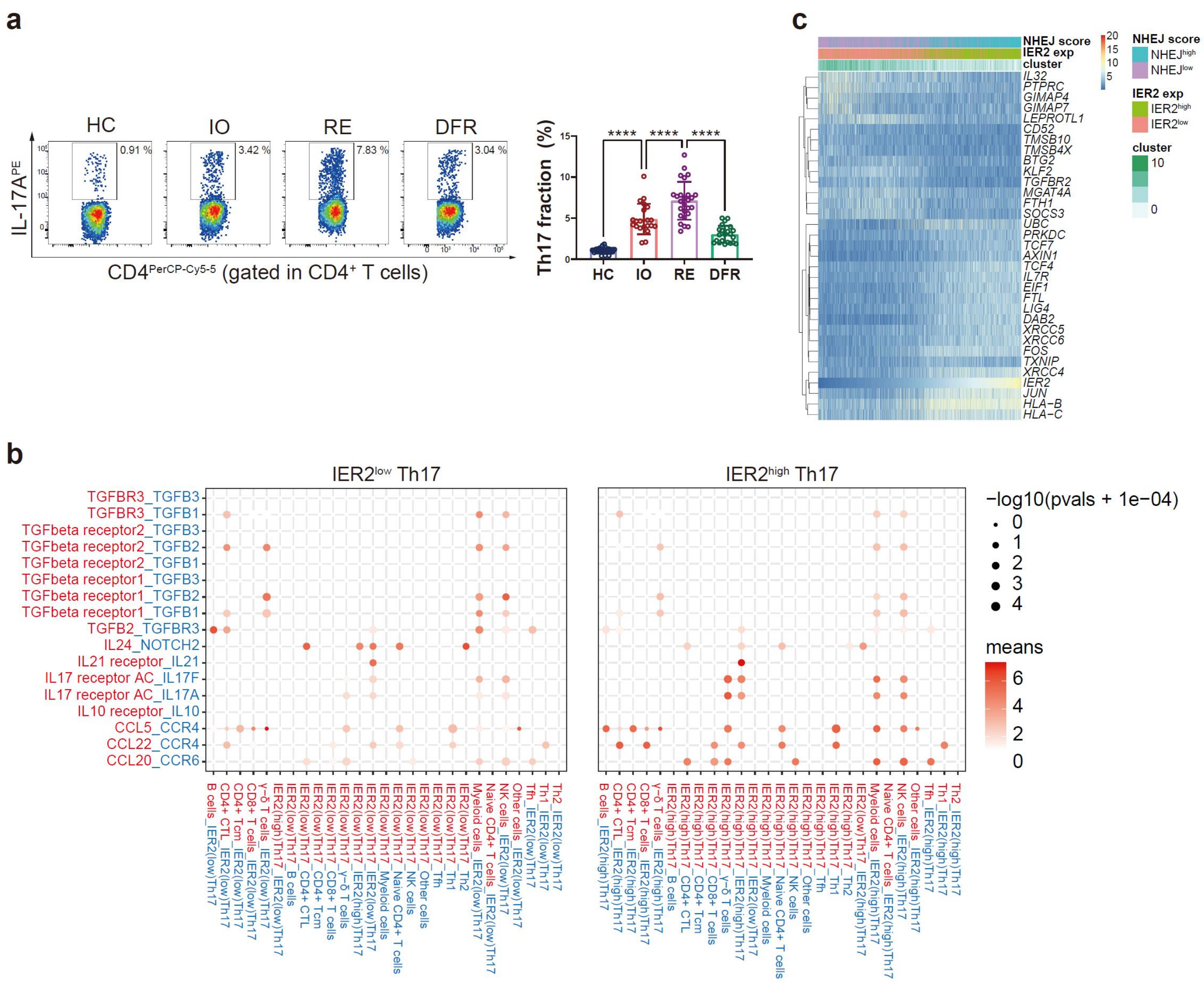

Fig. S9. IER2^{high} Th17 displayed a pro-inflammatory phenotype. Related to Figure 7.

a. Representative FC plots and statistical graph showing the frequencies of Th17 cells in PBMC-sample from 4 groups (HC n = 26; IO n = 21; RE n = 26; DFR n = 22).

b. Dot plots showing the ligand-receptor pair of different cell-type with IER2^{high} Th17 or IER2^{low} Th17 cells respectively.

c. Heatmap showing the DEGs between IER2^{high} Th17 with IER2^{low} Th17 cells from human scRNA-seq data.

Statistics were calculated by one-way analysis of variance followed by Turkey test. Error bars represent mean ± SD. $*P < 0.05$; $**P < 0.01$, $***P < 0.001$, $****P < 0.0001$.
