# Supplementary material for: Sensing of DNA double-strand breaks by the NHEJ system stabilizes RORγt transcriptional activity and shapes Th17 pathogenicity in autoimmunity
Source: Cell Res. 2026 Jan 7;36(5):340–58. doi: 10.1038/s41422-025-01204-6 (PMC13092643; doi:10.1038/s41422-025-01204-6)
Supplement: Supplementary file 10 — Supplementary information, Fig. S10 [file 41422_2025_1204_MOESM10_ESM.pdf]

Figure S10

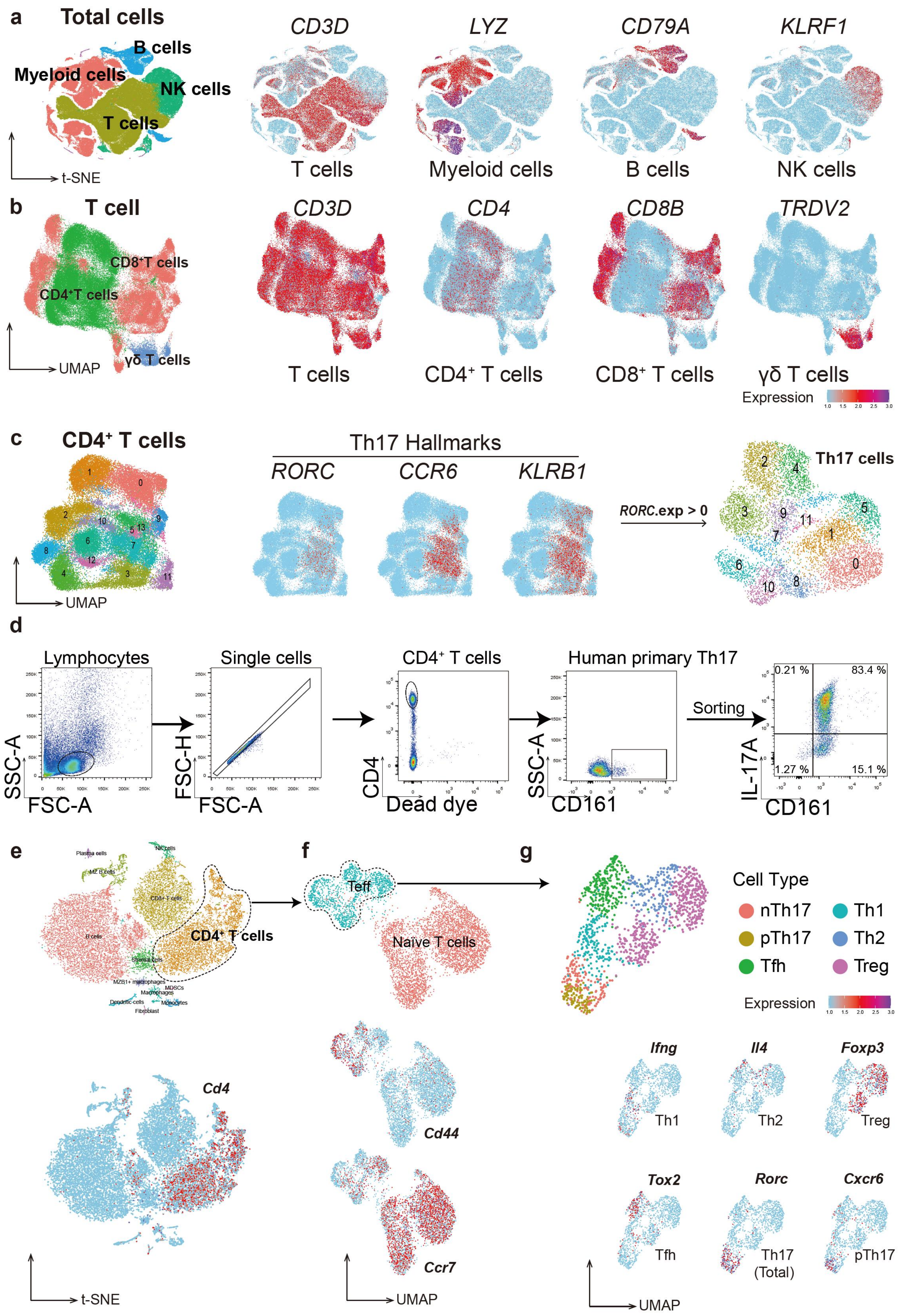

a
Total cells
B cells
Myeloid cells
NK cells
T cells
t-SNE
CD3D
LYZ
CD79A
KLRF1
T cells
Myeloid cells
B cells
NK cells
b
T cell
CD8⁺T cells
CD4⁺T cells
γδ T cells
UMAP
CD3D
CD4
CD8B
TRDV2
T cells
CD4⁺ T cells
CD8⁺ T cells
γδ T cells
Expression
c
CD4⁺ T cells
UMAP
Th17 Hallmarks
RORC
CCR6
KLRB1
RORC.exp > 0
Th17 cells
d
Lymphocytes
Single cells
CD4⁺ T cells
Human primary Th17
Sorting
SSC-A
FSC-A
FSC-H
FSC-A
CD4
Dead dye
SSC-A
CD161
IL-17A
CD161
0.21 %
83.4 %
1.27 %
15.1 %
e
Plasma cells
NK cells
MZ B cells
CD8+ T cells
B cells
CD4⁺ T cells
Stromal cells
MZB1+ macrophages
MDSCs
Macrophages
Dendritic cells
Monocytes
Fibroblast
Cd4
t-SNE
f
Teff
Naïve T cells
Cd44
Ccr7
UMAP
g
Cell Type
nTh17
Th1
pTh17
Th2
Tfh
Treg
Expression
Ifng
Il4
Foxp3
Th1
Th2
Treg
Tox2
Rorc
Cxcr6
Tfh
Th17 (Total)
pTh17
UMAP

Fig. S10. Cell clustering and annotation in scRNA-seq and sorting strategy of human primary Th17. Related to MATERIALS AND METHODS.

a. t-SNE projection plots showing all cells from human scRNA-seq data.

b. UMAP projection plots showing the sub-clusters of T cells from human scRNA-seq data.

c. UMAP projection plots showing the sub-clusters of $CD4^+$ T cells from human scRNA-seq data. The identification of primary Th17 was based on the specific transcriptional factor (RORγt, encoded by *RORC* gene) expressed in Th17. The cells with *RORC* gene expression > 0 was defined as Th17 cells.

d. IL-17A expression in human primary Th17 cells. We sorted $CD4^+$ $CD161^+$ cells from PBMCs and stained with anti-IL-17A and anti-CD161 antibodies for FC analysis.

e. t-SNE projection plots showing all cells from murine scRNA-seq data of EAU mice.

f. UMAP projection plots showing the sub-clusters of $CD4^+$ T cells from murine scRNA-seq data of EAU mice.

g. UMAP projection plots showing the sub-clusters of $CD4^+$ T effector cells from murine scRNA-seq data of EAU mice.
