# Supplementary material for: Sensing of DNA double-strand breaks by the NHEJ system stabilizes RORγt transcriptional activity and shapes Th17 pathogenicity in autoimmunity
Source: Cell Res. 2026 Jan 7;36(5):340–58. doi: 10.1038/s41422-025-01204-6 (PMC13092643; doi:10.1038/s41422-025-01204-6)
Supplement: Supplementary file 11 — Supplementary information, Fig. S11 [file 41422_2025_1204_MOESM11_ESM.pdf]

Figure S11

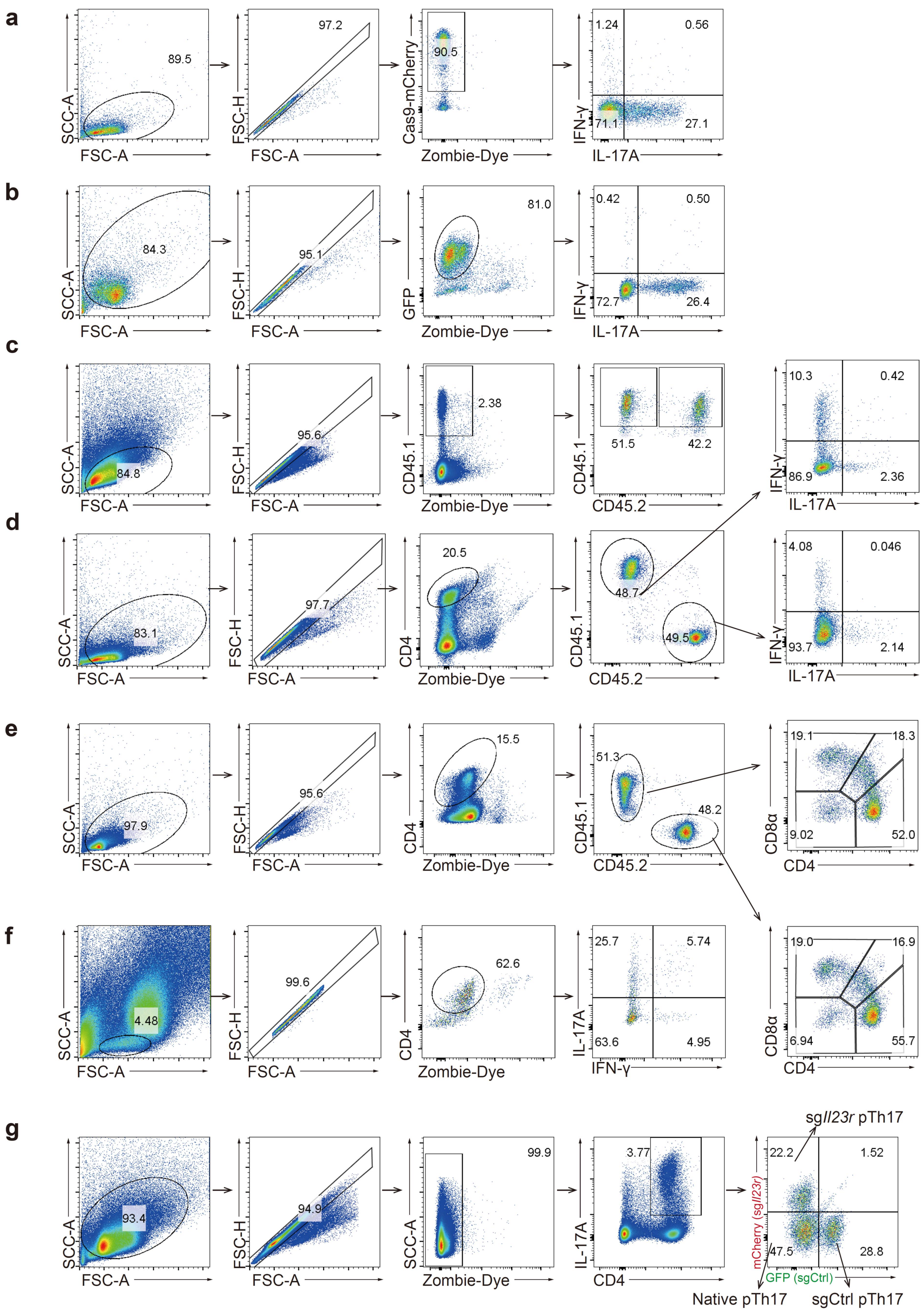
a
b
c
d
e
f
g
SCC-A
FSC-A
FSC-H
Cas9-mCherry
Zombie-Dye
IFN-γ
IL-17A
GFP
CD45.1
CD45.2
CD4
CD8α
mCherry (sgIl23r)
GFP (sgCtrl)
sgIl23r pTh17
Native pTh17
sgCtrl pTh17
89.5
97.2
90.5
1.24
0.56
71.1
27.1
84.3
95.1
81.0
0.42
0.50
72.7
26.4
84.8
95.6
2.38
51.5
42.2
10.3
0.42
86.9
2.36
83.1
97.7
20.5
48.7
49.5
4.08
0.046
93.7
2.14
97.9
95.6
15.5
51.3
48.2
19.1
18.3
9.02
52.0
4.48
99.6
62.6
25.7
5.74
63.6
4.95
19.0
16.9
6.94
55.7
93.4
94.9
99.9
3.77
22.2
1.52
47.5
28.8

Fig. S11. The gating strategies of FC analysis in this study. Related to MATERIALS AND METHODS.

a. Gating strategy for analyzing the transduction efficiency of sgRNA/CRISPR knock-out in human primary naïve $CD4^+$ T cells.

b. Gating strategy for analyzing the transduction efficiency of gene over-expression in human primary naïve $CD4^+$ T cells.

c. General gating strategy for analyzing the 1:1 adoptive transfer of $CD45.1/1^+$ control $CD4^+$ T cells and $CD45.1/2^+$ $CD4^+$ T cells with indicated treatment to EAU mice.

d. Gating strategy for analyzing $IL\text{-}17A^+$ and $IFN\text{-}\gamma^+$ $CD4^+$ T cells in dLN of BM-chimeric mice.

e. Gating strategy for analyzing the single-positive (SP), double-positive (DP) and double-negative (DN) thymocytes in thymus of BM-chimeric mice.

f. Gating strategy for analyzing $IL\text{-}17A^+$ and $IFN\text{-}\gamma^+$ $CD4^+$ T cells in retina of EAU *Rag1*$^{-/-}$ mice transferred with *in vitro* polarized murine pTh17 cells.

g. Gating strategy for analyzing the transferred Th17 cells in dLNs from the EAU recipients that received sgCtrl-$Cas9^{GFP}$ pTh17s and sg*Il23r*- $Cas9^{mCherry}$ pTh17 cells at day 2, 5, 8, 11 and 14 (n = 3 each time point).
