# Supplementary material for: Sensing of DNA double-strand breaks by the NHEJ system stabilizes RORγt transcriptional activity and shapes Th17 pathogenicity in autoimmunity
Source: Cell Res. 2026 Jan 7;36(5):340–58. doi: 10.1038/s41422-025-01204-6 (PMC13092643; doi:10.1038/s41422-025-01204-6)
Supplement: Supplementary file 12 — Supplementary information, Fig. S12 [file 41422_2025_1204_MOESM12_ESM.pdf]

Figure. S12

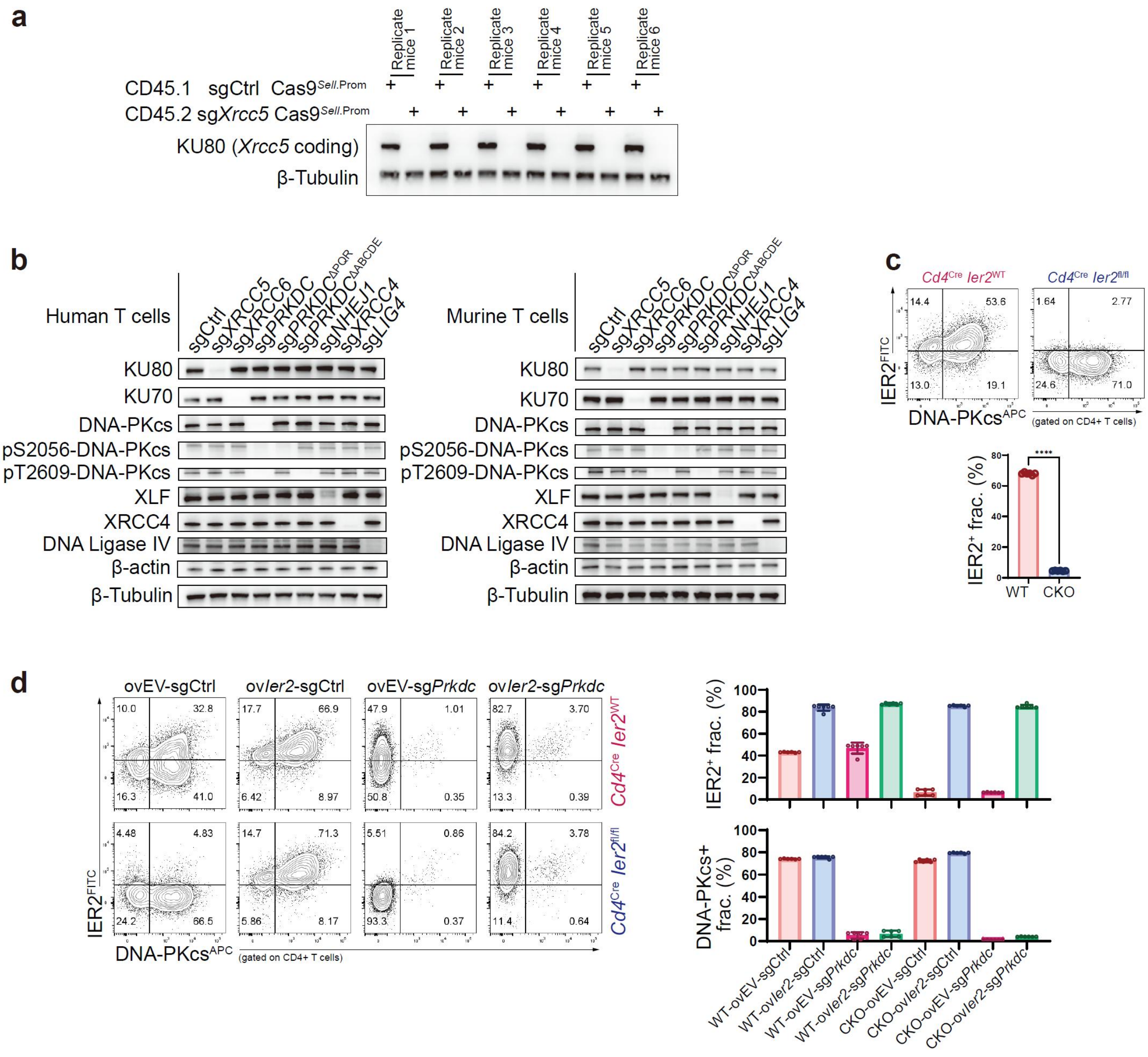

a
Replicate mice 1
Replicate mice 2
Replicate mice 3
Replicate mice 4
Replicate mice 5
Replicate mice 6
CD45.1 sgCtrl Cas9Sell.Prom
CD45.2 sgXrcc5 Cas9Sell.Prom
KU80 (Xrcc5 coding)
β-Tubulin
b
Human T cells
Murine T cells
sgCtrl
sgXRCC5
sgXRCC6
sgPRKDC
sgPRKDCΔPQR
sgPRKDCΔABCDE
sgNHEJ1
sgXRCC4
sgLIG4
KU80
KU70
DNA-PKcs
pS2056-DNA-PKcs
pT2609-DNA-PKcs
XLF
XRCC4
DNA Ligase IV
β-actin
β-Tubulin
c
Cd4Cre Ier2WT
Cd4Cre Ier2fl/fl
IER2FITC
DNA-PKcsAPC
(gated on CD4+ T cells)
14.4
53.6
13.0
19.1
1.64
2.77
24.6
71.0
IER2+ frac. (%)
WT
CKO
****
d
ovEV-sgCtrl
ovIer2-sgCtrl
ovEV-sgPrkdc
ovIer2-sgPrkdc
Cd4Cre Ier2WT
Cd4Cre Ier2fl/fl
10.0
32.8
16.3
41.0
17.7
66.9
6.42
8.97
47.9
1.01
50.8
0.35
82.7
3.70
13.3
0.39
4.48
4.83
24.2
66.5
14.7
71.3
5.86
8.17
5.51
0.86
93.3
0.37
84.2
3.78
11.4
0.64
IER2FITC
DNA-PKcsAPC
(gated on CD4+ T cells)
IER2+ frac. (%)
DNA-PKcs+ frac. (%)
WT-ovEV-sgCtrl
WT-ovIer2-sgCtrl
WT-ovEV-sgPrkdc
WT-ovIer2-sgPrkdc
CKO-ovEV-sgCtrl
CKO-ovIer2-sgCtrl
CKO-ovEV-sgPrkdc
CKO-ovIer2-sgPrkdc

Fig. S12. The efficiency of gene knock-out using CRISPR strategy. Related to MATERIALS AND METHODS.

a. Western-blot for verifying the knock-out efficiency of *Xrcc5* in sorted CD45.2/2^{+} peripheral T cells from the bone-marrow chimeric mice reconstituted by CD45.1/1^{+} sgCtrl-Cas9$^{\mathit{Sell}.\mathrm{Promoter}}$ and CD45.2/2^{+} sg*Xrcc5*-Cas9$^{\mathit{Sell}.\mathrm{Promoter}}$ BM cells (n = 6). Related to Fig. 1d.

b. Western-blot for verifying the knock-out efficiency of each NHEJ factor in human and murine polarized pTh17 cells (n = 3). Related to MATERIAL AND METHODS.

c. FC analysis for verifying IER2 expression in WT (*Cd4*^{Cre} *Ier2*^{WT}) and IER2-CKO (*Cd4*^{Cre} *Ier2*$^{\mathrm{fl/fl}}$) strain (n = 6). Related to MATERIAL AND METHODS.

d. FC analysis for verifying the expression of IER2 and DNA-PKcs in WT (*Cd4*^{Cre} *Ier2*^{WT}) and IER2-CKO (*Cd4*^{Cre} *Ier2*$^{\mathrm{fl/fl}}$) strain with over-expression of *Ier2* or knock-out of *Prkdc* (n = 6). Related to Fig. 6a.
