# Supplementary material for: Sensing of DNA double-strand breaks by the NHEJ system stabilizes RORγt transcriptional activity and shapes Th17 pathogenicity in autoimmunity
Source: Cell Res. 2026 Jan 7;36(5):340–58. doi: 10.1038/s41422-025-01204-6 (PMC13092643; doi:10.1038/s41422-025-01204-6)
Supplement: Supplementary file 13 — Supplementary information, Fig. S13 [file 41422_2025_1204_MOESM13_ESM.pdf]

Figure. S13

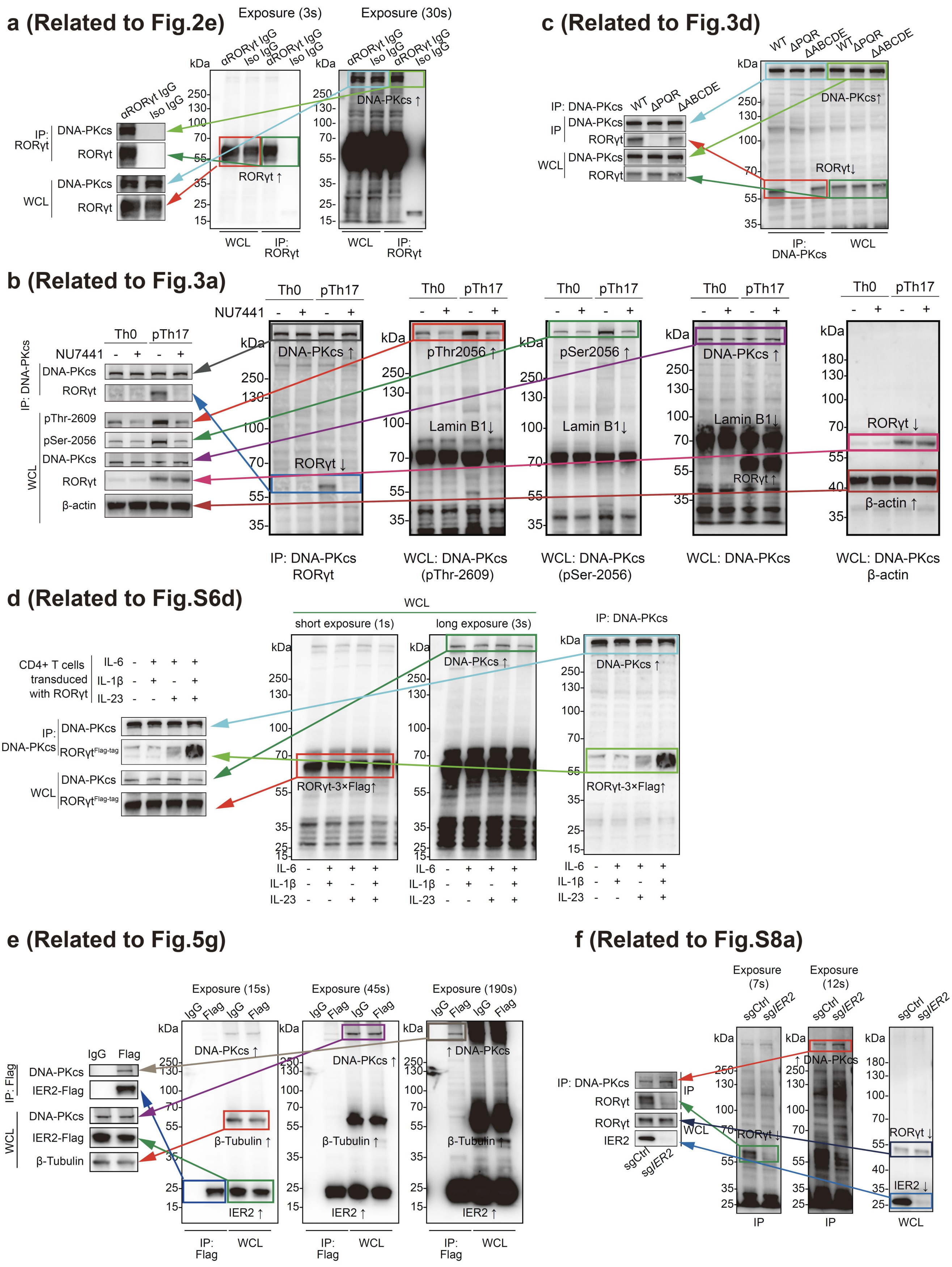

a (Related to Fig.2e)
Exposure (3s)
Exposure (30s)
αRORγt IgG
Iso IgG
IP: RORγt
DNA-PKcs
RORγt
WCL
DNA-PKcs ↑
RORγt ↑
IP: RORγt
c (Related to Fig.3d)
WT
ΔPQR
ΔABCDE
IP: DNA-PKcs
IP
WCL
DNA-PKcs↑
RORγt↓
IP: DNA-PKcs
b (Related to Fig.3a)
Th0
pTh17
NU7441
IP: DNA-PKcs
pThr-2609
pSer-2056
β-actin
pThr2056 ↑
pSer2056 ↑
Lamin B1↓
RORγt ↓
β-actin ↑
IP: DNA-PKcs RORγt
WCL: DNA-PKcs (pThr-2609)
WCL: DNA-PKcs (pSer-2056)
WCL: DNA-PKcs
WCL: DNA-PKcs β-actin
d (Related to Fig.S6d)
short exposure (1s)
long exposure (3s)
CD4+ T cells transduced with RORγt
IL-6
IL-1β
IL-23
IP: DNA-PKcs
RORγtFlag-tag
RORγt-3×Flag↑
e (Related to Fig.5g)
Exposure (15s)
Exposure (45s)
Exposure (190s)
IgG
Flag
IP: Flag
IER2-Flag
β-Tubulin
β-Tubulin ↑
IER2 ↑
f (Related to Fig.S8a)
Exposure (7s)
Exposure (12s)
sgCtrl
sgIER2
IER2
IER2 ↓
kDa

Fig. S13. The full-gel images for co-IP experiments.

- **a.** Full-gel images for Co-IP assay showing the interaction between DNA-PKcs and RORγt in human polarized pTh17 cells (Related to Fig. 2e).
- **b.** Full-gel images for Co-IP assay showing the interaction between DNA-PKcs and RORγt treated with or without 100 nM NU7441 (Related to Fig. 3a).
- **c.** Full-gel images for Co-IP assay showing the interaction between DNA-PKcs and RORγt in sgCtrl, sg*PRKDC*$^{\Delta PQR}$ and *sgPRKDC*$^{\Delta ABCDE}$ human polarized pTh17 cells (Related to Fig. 3d).
- **d.** Full-gel images for Co-IP assay showing the interaction of DNA-PKcs and RORγt in human T cells over-expressed with RORγt at indicated cytokine stimulation (Related to Fig. S6d).
- **e.** Full-gel images for Co-IP assay showing FLAG-IER2 interacted with p-DNA-PKcs in HP Th17 over-expressed with ectopic IER2 (Related to Fig. 5g)
- **f.** Full-gel images for Co-IP assay showing the interaction of DNA-PKcs and RORγt in polarized human pTh17 transduced with CRISPR system targeting on *IER2* (Related to Fig. S8a).
