# Supplementary material for: Sensing of DNA double-strand breaks by the NHEJ system stabilizes RORγt transcriptional activity and shapes Th17 pathogenicity in autoimmunity
Source: Cell Res. 2026 Jan 7;36(5):340–58. doi: 10.1038/s41422-025-01204-6 (PMC13092643; doi:10.1038/s41422-025-01204-6)
Supplement: Supplementary file 14 — Supplementary information, Table S1 [file 41422_2025_1204_MOESM14_ESM.pdf]

Table S1 - Clinical characteristics of 26 healthy donors and 70 AU patients (Related to Figure 7)

Sample ID	Age	Gender	Eye	Disease duration	Inflam-mation*	Other disease	Steroid (mg^{qd})	MTX (mg^{qw})	MMF (g^{Bid})	CSA (mg^{Bid})	ADA (mg^{q2w})	Result
HC_01	49	Male	NA	NA	-	-	0	0	0	0	0	Fig.7A-J, Fig. S9A-C
HC_02	24	Male	NA	NA	-	-	0	0	0	0	0	Fig.7G, Fig. S9A
HC_03	22	Female	NA	NA	-	-	0	0	0	0	0	Fig.7A-J, Fig. S9A-C
HC_04	30	Female	NA	NA	-	-	0	0	0	0	0	Fig.7G, Fig. S9A
HC_05	39	Female	NA	NA	-	-	0	0	0	0	0	Fig.7G, Fig. S9A
HC_06	37	Male	NA	NA	-	-	0	0	0	0	0	Fig.7A-J, Fig. S9A-C
HC_07	23	Female	NA	NA	-	-	0	0	0	0	0	Fig.7G, Fig. S9A
HC_08	50	Female	NA	NA	-	-	0	0	0	0	0	Fig.7G, Fig. S9A
HC_09	57	Female	NA	NA	-	-	0	0	0	0	0	Fig.7G, Fig. S9A
HC_10	48	Female	NA	NA	-	-	0	0	0	0	0	Fig.7G, Fig. S9A
HC_11	56	Female	NA	NA	-	-	0	0	0	0	0	Fig.7G, Fig. S9A
HC_12	40	Female	NA	NA	-	-	0	0	0	0	0	Fig.7G, Fig. S9A
HC_13	56	Female	NA	NA	-	-	0	0	0	0	0	Fig.7G, Fig. S9A
HC_14	48	Female	NA	NA	-	-	0	0	0	0	0	Fig.7G, Fig. S9A
HC_15	36	Female	NA	NA	-	-	0	0	0	0	0	Fig.7G, Fig. S9A
HC_16	25	Female	NA	NA	-	-	0	0	0	0	0	Fig.7G, Fig. S9A
HC_17	56	Female	NA	NA	-	-	0	0	0	0	0	Fig.7G, Fig. S9A
HC_18	29	Female	NA	NA	-	-	0	0	0	0	0	Fig.7G, Fig. S9A
HC_19	35	Female	NA	NA	-	-	0	0	0	0	0	Fig.7G, Fig. S9A
HC_20	40	Female	NA	NA	-	-	0	0	0	0	0	Fig.7A-J, Fig. S9A-C
HC_21	48	Male	NA	NA	-	-	0	0	0	0	0	Fig.7G, Fig. S9A
HC_22	47	Male	NA	NA	-	-	0	0	0	0	0	Fig.7G, Fig. S9A
HC_23	24	Male	NA	NA	-	-	0	0	0	0	0	Fig.7G, Fig. S9A
HC_24	20	Female	NA	NA	-	-	0	0	0	0	0	Fig.7G, Fig. S9A
HC_25	46	Female	NA	NA	-	-	0	0	0	0	0	Fig.7G, Fig. S9A
HC_26	44	Male	NA	NA	-	-	0	0	0	0	0	Fig.7G, Fig. S9A
IO_01	45	Female	OU	0	+	-	0	0	0	0	0	Fig.7G, Fig. S9A
IO_02	26	Female	OU	0	+	-	0	0	0	0	0	Fig.7G, Fig. S9A
IO_03	26	Female	OU	0	+	-	0	0	0	0	0	Fig.7A-J, Fig. S9A-C
IO_04	50	Female	OU	0	+	-	0	0	0	0	0	Fig.7G, Fig. S9A
IO_05	32	Female	OU	0	+	-	0	0	0	0	0	Fig.7A-J, Fig. S9A-C
IO_06	66	Male	OU	0	+	-	0	0	0	0	0	Fig.7G, Fig. S9A
IO_07	49	Male	OU	0	+	-	0	0	0	0	0	Fig.7A-J, Fig. S9A-C
IO_08	29	Female	OU	0	+	-	0	0	0	0	0	Fig.7G, Fig. S9A
IO_09	26	Female	OU	0	+	-	0	0	0	0	0	Fig.7G, Fig. S9A
IO_10	40	Female	OU	0	+	-	0	0	0	0	0	Fig.7G, Fig. S9A
IO_11	38	Female	OU	0	+	-	0	0	0	0	0	Fig.7G, Fig. S9A
IO_12	72	Female	OU	0	+	-	0	0	0	0	0	Fig.7G, Fig. S9A
IO_13	55	Male	OU	0	+	-	0	0	0	0	0	Fig.7A-J, Fig. S9A-C
IO_14	51	Female	OU	0	+	-	0	0	0	0	0	Fig.7G, Fig. S9A

IO_15	50	Female	OU	0	+	-	0	0	0	0	0	Fig.7G, Fig. S9A
IO_16	20	Male	OU	0	+	-	0	0	0	0	0	Fig.7G, Fig. S9A
IO_17	46	Female	OU	0	+	-	0	0	0	0	0	Fig.7G, Fig. S9A
IO_18	44	Male	OU	0	+	-	0	0	0	0	0	Fig.7G, Fig. S9A
IO_19	49	Female	OU	0	+	-	0	0	0	0	0	Fig.7G, Fig. S9A
IO_20	71	Male	OU	0	+	-	0	0	0	0	0	Fig.7G, Fig. S9A
IO_21	31	Female	OU	0	+	-	0	0	0	0	0	Fig.7G, Fig. S9A
RE_01	40	Female	OU	2226	+	-	0	7.5	0	0	0	Fig.7A-J, Fig. S9A-C
RE_02	27	Female	OU	844	+	-	0	0	0.5	0	0	Fig.7A-J, Fig. S9A-C
RE_03	66	Female	OU	856	+	-	5	0	0	0	0	Fig.7G, Fig. S9A
RE_04	53	Female	OU	113	+	-	10	0	0.5	0	40	Fig.7G, Fig. S9A
RE_05	56	Male	OU	3763	+	-	0	0	0.5	50	0	Fig.7A-J, Fig. S9A-C
RE_06	28	Female	OU	122	+	-	0	15	0	0	0	Fig.7G, Fig. S9A
RE_07	44	Female	OU	417	+	-	10	15	0	0	40	Fig.7G, Fig. S9A
RE_08	57	Female	OU	1587	+	-	10	0	0	0	0	Fig.7G, Fig. S9A
RE_09	63	Male	OU	734	+	-	0	15	0	0	0	Fig.7G, Fig. S9A
RE_10	52	Female	OU	269	+	-	7.5	0	0	0	40	Fig.7G, Fig. S9A
RE_11	67	Male	OU	7484	+	-	0	0	0	0	0	Fig.7G, Fig. S9A
RE_12	25	Male	OU	671	+	-	3	0	0.5	0	0	Fig.7G, Fig. S9A
RE_13	18	Female	OU	516	+	-	10	0	0.5	0	40	Fig.7G, Fig. S9A
RE_14	30	Female	OU	795	+	-	0	0	0	0	40	Fig.7G, Fig. S9A
RE_15	41	Male	OU	1510	+	-	10	0	0	0	0	Fig.7G, Fig. S9A
RE_16	22	Female	OU	2250	+	-	10	0	0	0	0	Fig.7G, Fig. S9A
RE_17	37	Female	OU	734	+	-	0	15	0	0	40	Fig.7G, Fig. S9A
RE_18	50	Male	OU	199	+	-	10	15	0	0	0	Fig.7G, Fig. S9A
RE_19	26	Male	OU	634	+	-	20	15	0.5	0	40	Fig.7G, Fig. S9A
RE_20	31	Male	OU	338	+	-	0	15	0	0	40	Fig.7A-J, Fig. S9A-C
RE_21	40	Female	OU	292	+	-	10	15	0	0	0	Fig.7G, Fig. S9A
RE_22	35	Male	OU	2798	+	-	15	0	0.5	0	0	Fig.7G, Fig. S9A
RE_23	56	Female	OU	4793	+	-	0	15	0	0	0	Fig.7G, Fig. S9A
RE_24	33	Female	OU	537	+	-	0	15	0	0	0	Fig.7G, Fig. S9A
RE_25	29	Male	OU	250	+	-	0	15	0	0	0	Fig.7G, Fig. S9A
RE_26	24	Male	OU	1097	+	-	5	0	0	0	0	Fig.7G, Fig. S9A
RE_27	69	Female	OU	366	+	-	0	15	0	0	0	Fig.7G, Fig. S9A
DFR_01	43	Female	OU	1338	-	-	0	0	0	0	0	Fig.7G, Fig. S9A
DFR_02	30	Male	OU	1196	-	-	0	0	0	0	0	Fig.7G, Fig. S9A
DFR_03	32	Male	OU	4456	-	-	0	0	0	0	0	Fig.7A-J, Fig. S9A-C
DFR_04	47	Female	OU	1096	-	-	0	0	0	0	0	Fig.7A-J, Fig. S9A-C
DFR_05	18	Female	OU	1190	-	-	0	0	0	0	0	Fig.7G, Fig. S9A
DFR_06	21	Male	OU	1122	-	-	0	0	0	0	0	Fig.7A-J, Fig. S9A-C
DFR_07	36	Male	OU	1561	-	-	0	0	0	0	0	Fig.7G, Fig. S9A
DFR_08	58	Male	OU	2258	-	-	0	0	0	0	0	Fig.7G, Fig. S9A
DFR_09	38	Female	OU	1356	-	-	0	0	0	0	0	Fig.7G, Fig. S9A
DFR_10	38	Female	OU	1244	-	-	0	0	0	0	0	Fig.7G, Fig. S9A

DFR_11	33	Female	OU	1472	-	-	0	0	0	0	0	Fig.7G, Fig. S9A
DFR_12	63	Male	OU	1402	-	-	0	0	0	0	0	Fig.7G, Fig. S9A
DFR_13	28	Female	OU	4455	-	-	0	0	0	0	0	Fig.7G, Fig. S9A
DFR_14	32	Female	OU	1626	-	-	0	0	0	0	0	Fig.7G, Fig. S9A
DFR_15	46	Female	OU	1812	-	-	0	0	0	0	0	Fig.7G, Fig. S9A
DFR_16	51	Female	OU	1238	-	-	0	0	0	0	0	Fig.7A-J, Fig. S9A-C
DFR_17	43	Female	OU	1039	-	-	0	0	0	0	0	Fig.7G, Fig. S9A
DFR_18	45	Female	OU	1990	-	-	0	0	0	0	0	Fig.7G, Fig. S9A
DFR_19	47	Male	OU	1298	-	-	0	0	0	0	0	Fig.7G, Fig. S9A
DFR_20	30	Male	OU	1689	-	-	0	0	0	0	0	Fig.7G, Fig. S9A
DFR_21	65	Male	OU	1486	-	-	0	0	0	0	0	Fig.7G, Fig. S9A
DFR_22	35	Female	OU	1425	-	-	0	0	0	0	0	Fig.7G, Fig. S9A

Clinical characteristics of 26 healthy control donors (HC), 21 initial-onset (IO) patients, 27 refractory-relapse (RE) patients and 22 drug-free remission (DFR) patients among each individual. AU, autoimmune uveitis. OU, double-eyes. MTX, methotrexate. MMF, mycophenolate mofetil. CSA, ciclosporin A. ADA, adalimumab.

Inflammation*: Including anterior chamber cells, vitreous cells, exudative retinal detachment (OCT), choroidal thickening (OCT), diffuse choroiditis (ICGA) or vascular leakage (FFA). Any one of them included could be considered as inflammation
