# Supplementary material for: Sensing of DNA double-strand breaks by the NHEJ system stabilizes RORγt transcriptional activity and shapes Th17 pathogenicity in autoimmunity
Source: Cell Res. 2026 Jan 7;36(5):340–58. doi: 10.1038/s41422-025-01204-6 (PMC13092643; doi:10.1038/s41422-025-01204-6)
Supplement: Supplementary file 15 — Supplementary information, Table S2 [file 41422_2025_1204_MOESM15_ESM.pdf]

Table S2 - Summary of the clinical characteristics of 26 healthy donors and 70 AU patients (Related to Figure 7)

	Healthy control (HC)	Initial onset (IO)	Recurrent relapse (RE)	Drug-free remission (DFR)
**Number**	26	21	27	22
**Age (Years; Mean ± SD)**	39.58 ± 9.755	43.62 ± 14.88	41.44 ± 15.36	39.95 ± 12.33
**Sex (Male/Female)**	7/19	6/15	11/16	9/13
**Disease duration (Days; Mean±SD)**	-	0 ± 0	1341 ± 1684	1716 ± 935
**Current treatment**	-	-	Steroid (14/27) Methotrexate (13/27) Mycophenolate Mofetil (7/27) Cyclosporin (1/27) Adalimumab (8/27)	-

The summary of the information of the HC donors and AU patients listed in Table S1.
