# Supplementary material for: Sensing of DNA double-strand breaks by the NHEJ system stabilizes RORγt transcriptional activity and shapes Th17 pathogenicity in autoimmunity
Source: Cell Res. 2026 Jan 7;36(5):340–58. doi: 10.1038/s41422-025-01204-6 (PMC13092643; doi:10.1038/s41422-025-01204-6)
Supplement: Supplementary file 16 — Supplementary information, Table S3 [file 41422_2025_1204_MOESM16_ESM.pdf]

Table S3 – sgRNAs. Related to ONLINE METHODS.

sgRNA target	Species	sgRNA1	sgRNA2
XRCC5	Homo sapiens	AATCCAACCAGGTTCTCAAC	CCGATTCAGCAAAAGTCAGC
XRCC6	Homo sapiens	GTTGCGGAAGGTTCGCGCCA	TTAATACAAGTACAGGCGGT
PRKDC	Homo sapiens	GCCGGTCATCAACTGATCCG	TTGTCCGCTGCGGACCGCTG
PRKDC$^{\Delta PQR}$	Homo sapiens	GTGTACCAAACTCTTATGCC	TATACTCCGAGGGTTGTCAA
PRKDC$^{\Delta ABCDE}$	Homo sapiens	GGCACTGAGGCGCACGTACC	GGGCTTGATTACGAATGAGA
LIG4	Homo sapiens	GCTTATACGGATGATCATAA	TACAGCAAGTAAACGACCTT
XRCC4	Homo sapiens	GCTGATGTATACACGTTTAA	AGCTGAAGTCATTAGAGAAC
NHEJ1	Homo sapiens	GTTGATGCAGCCATGGGCGT	TCGCCCATTGTTGAAGGACG
Xrcc5	Mus musculus	GAGATTGCGTTAGTCCTCTA	ATCGACAAGAATGGCGAGCC
Xrcc6	Mus musculus	AAGCAGCGATCGGGATCTCC	AGACACGGTTGGCCATGGGT
Prkdc	Mus musculus	CAGTAGCCAACACCGTACGC	AGGGAACCGGCGTACGGTGT
Prkdc$^{\Delta PQR}$	Mus musculus	TAGGCGAGACAATAGCTGCT	AGATCTAAGCAGGTCTCTCA
Prkdc$^{\Delta ABCDE}$	Mus musculus	GTACAGGGTCAGCAAGTTTC	CATCAAGCTCAAGTCGTTAG
Lig4	Mus musculus	ACAAAGATGGCGCGCTGTAC	TACAACTATACCGACCAGTT
Xrcc4	Mus musculus	CTTTTCTAAGGAGTCTCGGC	TCAACCTAGACAAAGTTTCA
Nhej1	Mus musculus	GGATGAAGCACTTCGCCCAC	GCTACTTTCTCCTGTGACCG
PYGL	Homo sapiens	ACGGCATTCGGTATGAATAT	ACACCGGGACCAAGTGGATT
TRMT10A	Homo sapiens	AGATTAGGTGAAGGGTGTGA	TCGCCTTATTATTGACTGTA
IER2	Homo sapiens	TATCACTCCCGCATGCAGCG	TCGAGCCCGAGGTGTCGTTG
HSP90B1	Homo sapiens	AATCTGGGACAAGCGAGTTT	CGCCTTCCTTGTAGCAGATA
YWHAB	Homo sapiens	CCGCTCTTCCTGGCGTGTCA	GAAAGCCAAACTCGCTGAGC

APC	Homo sapiens	GCAAGTTGAGGCACTGAAGA	GAAGTACTTAAACAACTACA
YWHAZ	Homo sapiens	AAAATGTTGTAGGAGCCCGT	GGCTGAGCGATATGATGACA
EPB41L2	Homo sapiens	GAATCAGTCTTCCGATCCAG	TTTCTCGGTTCATACCGCCA
IL23R	Homo sapiens	GAATCAGGTCACTATTCAAT	AGCAGCAATTAAGAACTGCC
Il23r	Mus musculus	ATAGAACAACAGCTCGGATT	CCCTTAAGCACTGCCGACCA
