# Supplementary material for: Sensing of DNA double-strand breaks by the NHEJ system stabilizes RORγt transcriptional activity and shapes Th17 pathogenicity in autoimmunity
Source: Cell Res. 2026 Jan 7;36(5):340–58. doi: 10.1038/s41422-025-01204-6 (PMC13092643; doi:10.1038/s41422-025-01204-6)
Supplement: Supplementary file 17 — Supplementary information, Table S4 [file 41422_2025_1204_MOESM17_ESM.pdf]

Table S4 - shRNAs. Related to ONLINE METHODS.

shRNA Target	Species	shRNA sequence
IER2	Homo sapiens	5'CCGGCTGGTCTGAATCACGAGAATGCTCGAGCATTCTCGTGATTCAGACCAGTTTTTT- 3'
Ier2	Mus musculus	5'CCGGCGTGCGTCCATAGGTGCTATTCTCGAGAATAGCACCTATGGACGCACGTTTTTT- 3'
