# Supplementary material for: Sensing of DNA double-strand breaks by the NHEJ system stabilizes RORγt transcriptional activity and shapes Th17 pathogenicity in autoimmunity
Source: Cell Res. 2026 Jan 7;36(5):340–58. doi: 10.1038/s41422-025-01204-6 (PMC13092643; doi:10.1038/s41422-025-01204-6)
Supplement: Supplementary file 18 — Supplementary information, Table S5 [file 41422_2025_1204_MOESM18_ESM.pdf]

Table S5 - HDR donor templates. Related to ONLINE METHODS.

Target	Species	HDR donor template
PRKDC$^{\Delta PQR}$ HDR template	Homo sapiens	5’TTACTTCTATATTTTACAACAATTACAAGCACTATGATA<br>TATCATAACTAGATTTGAATAAATTGAGGTTCAGAGATA<br>AATAGAATTTATCAGCAGTCCAGAGTTAATTTGGAGAAT<br>TTTCCAAGATCTAATTCCTAGCCTTTTATTCTGTAGACTC<br>AGCAGTCAGATATCATCAGCACAGTAGGAGGTCAGAGG<br>TCAAC-3’
PRKDC$^{\Delta ABCDE}$ HDR template	Homo sapiens	5’GGGGTTCTTAGCACCTTTCAGTAGATAACAAAGTGTTA<br>TTCCTGTGGTGGGAGCAAGTGGCGTGTTTCTTACTTTAC<br>AAATGGGGACACTGCGGTCCAGGTTTAAAATTCAGTTA<br>ATGTTGCCTAGGCACTCACCATGCTGAAGAAATTGTTTT<br>CTGGGAGTTCCAGAAGAATCCTTCAGTAAGTTATACTCG<br>ATCCATT-3’
Prkdc$^{\Delta PQR}$ HDR template	Mus musculus	5’TTAGGTAATATGGACAATGGGAAGTCAGGAGTAGCTG<br>GAAGATTGGCTCCAGCAGGTGGGACACAGTTGGTCTTA<br>TCCAGACTGCTTGCAGCCTCAGCCACTGTTAACTCAGC<br>AGTCAGATTTTATCAGTTAGAGGTCAGGACCTCAACAG<br>AGTCACCATACAGACAGCAGAGGTAAGACAGCAGACA<br>TGGAAAATCTGG-3’
Prkdc$^{\Delta ABCDE}$ HDR template	Mus musculus	5’GCAGAGGCTAGAGGAGGGCGCAATCCCCTGAAACTG<br>GAAATTGAAGTTACAGATGTTTTGTAAACTAATATGTGA<br>CTTCTGAGAACTGAAGCCAGGTCCTGGGTGGCAGCAG<br>GCCTCTTTACCATCCAGTCAACATTTTGTCTTAAGTTTTT<br>TACAGACCCTCCCTTTCCAGCATACTGCCTGACCATAGT<br>ATGGAGACC-3’
