# Supplementary material for: Sensing of DNA double-strand breaks by the NHEJ system stabilizes RORγt transcriptional activity and shapes Th17 pathogenicity in autoimmunity
Source: Cell Res. 2026 Jan 7;36(5):340–58. doi: 10.1038/s41422-025-01204-6 (PMC13092643; doi:10.1038/s41422-025-01204-6)
Supplement: Supplementary file 19 — Supplementary information, Table S6 [file 41422_2025_1204_MOESM19_ESM.pdf]

Table S6 - 6×Motif reporters. Related to ONLINE METHODS.

TF of 6×Motif reporter	Species	Motif Target
RORC	Homo sapiens	TAACTAGGTCA
BATF	Homo sapiens	TGACTCA
FOSL2	Homo sapiens	GATGACTCA
IRF4	Homo sapiens	CGAAACCGAAACTA
STAT3	Homo sapiens	CTTCTGGGAAA
