# Supplementary material for: Sensing of DNA double-strand breaks by the NHEJ system stabilizes RORγt transcriptional activity and shapes Th17 pathogenicity in autoimmunity
Source: Cell Res. 2026 Jan 7;36(5):340–58. doi: 10.1038/s41422-025-01204-6 (PMC13092643; doi:10.1038/s41422-025-01204-6)
Supplement: Supplementary file 20 — Supplementary information, Table S7 [file 41422_2025_1204_MOESM20_ESM.pdf]

Table S7 - *TCR*$_{IRBP1-20}$ sequence. Related to ONLINE METHODS.

***TCR*$_{IRBP1-20}$**	**Species**	**V(D)J locus**	**CDR3**
Tcra	Mus musculus	*Trav7n-6 Traj32 Trac*	TGTGCCAGTACCCAGGGGGCTGGGGAACAGTACTTC
Tcrb	Mus musculus	*Trbv13-1 Trbj2-7 Trbc2*	TGTGCAGTGAGCATGGATGGGAGCAGTGGCAACAAGCTCATCTTT
