# Supplementary material for: Sensing of DNA double-strand breaks by the NHEJ system stabilizes RORγt transcriptional activity and shapes Th17 pathogenicity in autoimmunity
Source: Cell Res. 2026 Jan 7;36(5):340–58. doi: 10.1038/s41422-025-01204-6 (PMC13092643; doi:10.1038/s41422-025-01204-6)
Supplement: Supplementary file 21 — Supplementary information, Table S8 [file 41422_2025_1204_MOESM21_ESM.pdf]

Table S8 – Antibodies used

Antibody name	Sourse	Identifier
PE/Cyanine7 anti-H2A.X Phospho (Ser139) Antibody	BioLegend	Cat#613420; RRID: AB_2715784
PerCP/Cyanine5.5 anti-mouse CD4 Antibody	BioLegend	Cat#100434; RRID: AB_893330
PE anti-mouse CD45.1 Antibody	BioLegend	Cat#110707; RRID: AB_313496
Brilliant Violet 510™ anti-mouse CD45.2 Antibody	BioLegend	Cat#109837; RRID: AB_2650900
APC anti-mouse IFN-γ Antibody	BioLegend	Cat#505810; RRID: AB_315404
Brilliant Violet 421™ anti-mouse IL-17A Antibody	BioLegend	Cat#506925; RRID: AB_2632611
Alexa Fluor® 647 anti-mouse IL-17A Antibody	BioLegend	Cat#506911; RRID: AB_536013
PE/Cyanine7 anti-mouse IL-2 Antibody	BioLegend	Cat#503831; RRID: AB_2561750
APC anti-mouse GM-CSF Antibody	BioLegend	Cat#505413; RRID: AB_2721461
Brilliant Violet 785™ anti-mouse CD8a Antibody	BioLegend	Cat#100750; RRID: AB_2562610
FITC anti-mouse CD3ε Antibody	BioLegend	Cat#100306; RRID: AB_312671
APC anti-mouse/human CD44 Antibody	BioLegend	Cat#103011; RRID: AB_312962
Brilliant Violet 421™ anti-mouse CD25 Antibody	BioLegend	Cat#102033; RRID: AB_10895908
Alexa Fluor® 647 anti-mouse/rat/human FOXP3 Antibody	BioLegend	Cat#320014; RRID: AB_439750
Alexa Fluor® 488 anti-mouse IL-4 Antibody	BioLegend	Cat#504111; RRID: AB_493320
Brilliant Violet 421™ anti-human IFN-γ Antibody	BioLegend	Cat#502531; RRID: AB_10900083
Alexa Fluor® 647 anti-human IL-17A Antibody	BioLegend	Cat#512309; RRID: AB_961389
ROR gamma (t) Monoclonal Antibody (AFKJS-9), PE, eBioscience™	eBioscience	Cat#12-6988-82; RRID: AB_1834470
ROR gamma (t) Monoclonal Antibody (AFKJS-9), APC, eBioscience™	eBioscience	Cat#17-6988-82; RRID: AB_10609207
FITC anti-human CD279 (PD-1) Antibody	BioLegend	Cat#379205; RRID: AB_2922605
PE/Cyanine5 anti-human CD69 Antibody	BioLegend	Cat#310907; RRID:

		AB_314843
BD Horizon™ BV650 Mouse Anti-Human IL-17A	BD	Cat#563746; RRID: AB_2738402
FITC anti-mouse CD62L Antibody	BioLegend	Cat#104406; RRID: AB_313093
FITC anti-mouse IL-17A Antibody	BioLegend	Cat#506908; RRID: AB_536010
APC anti-mouse Ly108 Antibody	BioLegend	Cat#134609; RRID: AB_2728154
PE anti-mouse CXCR6 Antibody	BioLegend	Cat#151103; RRID: AB_2566545
APC anti-mouse TCR β chain Antibody	BioLegend	Cat#109212; RRID: AB_313435
APC anti-mouse CD45RB Antibody	BioLegend	Cat#103319; RRID: AB_2565229
PE/Cyanine7 anti-human Ki-67 Antibody	BioLegend	Cat#350525; RRID: AB_2562872
PE anti-Annexin V antibody	BD	Cat#556421; RRID: AB_2869071
Rabbit Anti-phospho-DNA-PKcs/PRKDC(Ser2056) Polyclonal Antibody, APC-conjugated	Bioss	Cat#bs-3734R-APC; RRID: AB_10857401
Rabbit Anti-DNA PKcs/APC Conjugated antibody	Bioss	Cat#bs-1359R-APC; RRID: AB_10852744
Rabbit Anti-Ku-70/FITC Conjugated antibody	Bioss	Cat#bs-2294R-FITC ; RRID: N/A
Rabbit Anti-XRCC4/PE Conjugated antibody	Bioss	Cat#bs-8510R-PE; RRID: N/A
Rabbit Anti-Ku-80/PE-Cy5 Conjugated antibody	Bioss	Cat#bs-1358R-PE-Cy5; RRID: AB_11103040
Rabbit Anti-DNA Ligase IV/PE-Cy7 Conjugated antibody	Bioss	Cat#bs-1677R-PE-Cy7; RRID: N/A
Brilliant Violet 421™ anti-human CD3 Antibody	BioLegend	Cat#300434; RRID: AB_10962690
PE anti-human CD4 Antibody	BioLegend	Cat#300508; RRID: AB_314075
Brilliant Violet 510™ anti-human CD8a Antibody	BioLegend	Cat#300934; RRID: AB_2814114
IER2 Rabbit anti-Human Polyclonal (aa46-95) (FITC) Antibody	LSBio	Cat#LS-C431083 ; RRID: N/A
PE Mouse IgG1 kappa Isotype Control	eBioscience	Cat#12-4714-42; RRID: AB_1944423

APC Mouse IgG2b, κ Isotype Ctrl Antibody	BioLegend	Cat#401209; RRID: AB_2941901
Brilliant Violet 785™ anti-human CD196 (CCR6) Antibody	BioLegend	Cat#353421; RRID: AB_2561372
PerCP/Cyanine5.5 anti-human CD161 Antibody	BioLegend	Cat#339907; RRID: AB_2265447
CD161 Antibody, anti-human, REAfinity	Miltenyi Biotec	Cat#130-119-621; RRID: AB_2751761
Mouse recombinant anti-IL-23R Antibody	R&D Systems	Cat#MAB1686; RRID: AB_2124650
DNA-PKcs Polyclonal antibody	Proteintech Group	Cat#19983-1-AP; RRID: AB_10642811
RORC Polyclonal antibody	Proteintech Group	Cat#29910-1-AP; RRID: AB_2935489
Phospho-DNA-PKcs (Ser2056) (E9J4G) Rabbit mAb	Cell Signaling Technology	Cat#68716; RRID: AB_2939025
Lamin B1 Polyclonal antibody	Proteintech Group	Cat#12987-1-AP; RRID: AB_2136290
Rabbit Anti-phospho-DNA PK/PRKDC(Thr2609) Polyclonal Antibody, Unconjugated	Bioss	Cat#bs-3735R; RRID: AB_10856717
IER2 Polyclonal antibody	Proteintech Group	Cat#23849-1-AP; RRID: AB_2879339
Mouse DYKDDDDK tag Monoclonal antibody	Proteintech Group	Cat#66008-4-Ig; RRID: AB_2918475
Beta Tubulin Polyclonal antibody	Proteintech Group	Cat#10094-1-AP; RRID: AB_2210695
Anti-rabbit IgG, HRP-linked Antibody	Cell Signaling Technology	Cat#7074; RRID: AB_2099233
HRP-linked Rabbit Anti-Mouse IgG (Light Chain Specific) (D3V2A) mAb	Cell Signaling Technology	Cat#58802; RRID: AB_2799549
Anti-ROR gamma (t) Monoclonal Antibody	eBioscience	Cat#14-6988-80; RRID: AB_1311291
Anti-rabbit IgG (H+L), F(ab')2 Fragment (Alexa Fluor 488 Conjugate)	Cell Signaling Technology	Cat#4412; RRID: AB_1904025
Anti-rabbit IgG (H+L), F(ab')2 Fragment (Alexa Fluor 594 Conjugate)	Cell Signaling Technology	Cat#8889; RRID: AB_2716249
Rabbit DYKDDDDK tag Monoclonal antibody	Proteintech Group	Cat#80010-1-RR; RRID: AB_2882940
Rabbit Anti-Histone H2A.X, phospho (Ser139) Monoclonal Antibody	abcam	Cat#ab81299; RRID: AB_1640564
Anti-TRF1 antibody	abcam	Cat#ab192629; RRID: AB_3675954

Rabbit Anti-DNA-PKcs (non-phosphorylation site of S2056)	United States Biological	Cat#313732; RRID: N/A
Rabbit Anti-DNA-PKcs (non-phosphorylation site of T2647)	United States Biological	Cat#313728; RRID: N/A
DNA PKcs Rabbit pAb (151-300aa)	Bioss	Cat#bs-1359R; RRID: AB_10852744
DNA-PKcs Recombinant monoclonal antibody (4106-4125aa)	Proteintech Group	Cat#85370-2-RR; RRID: N/A
