# Supplementary material for: Sensing of DNA double-strand breaks by the NHEJ system stabilizes RORγt transcriptional activity and shapes Th17 pathogenicity in autoimmunity
Source: Cell Res. 2026 Jan 7;36(5):340–58. doi: 10.1038/s41422-025-01204-6 (PMC13092643; doi:10.1038/s41422-025-01204-6)
Supplement: Supplementary file 22 — Supplementary information, Table S9 [file 41422_2025_1204_MOESM22_ESM.pdf]

Table S9 – Devices

Devices	Source
BD LSRFortessa flow cytometer	BD
LSM 980 With Airyscan 2 confocal microscopy	ZEISS
Novaseq 6000 sequencer	Illumina
BD FACSAriaFusion sorter	BD
Chromium single cell controller	10x Genomics
Amaxa Nucleofector II device	Lonza
Micron IV Retinal Imaging Microscope	PHOENIX
