# Supplementary material for: Sensing of DNA double-strand breaks by the NHEJ system stabilizes RORγt transcriptional activity and shapes Th17 pathogenicity in autoimmunity
Source: Cell Res. 2026 Jan 7;36(5):340–58. doi: 10.1038/s41422-025-01204-6 (PMC13092643; doi:10.1038/s41422-025-01204-6)
Supplement: Supplementary file 23 — Supplementary information, Table S10 [file 41422_2025_1204_MOESM23_ESM.pdf]

Table S10 – Software

Software name	Sourse	Identifier
FlowJo 10.8.1	FlowJo LLC	https://www.flowjo.com/
GraphPad Prism V9	GraphPad Software	https://www.graphpad.com/
ImageJ	National Institutes of Health	https://imagej.nih.gov/ij/
Rstudio 4.0.3	Rstudio	www.rstudio.com/
IGV 2.8.10	National Institutes of Health	https://igv.org/
ZEN 2.3	ZEISS	https://www.zeiss.com.cn/corporate/home.html
Bowtie2 0.20.0	Johns Hopkins University	http://bowtie-bio.sourceforge.net/bowtie2/manual.shtml
