# Supplementary material for: Sensing of DNA double-strand breaks by the NHEJ system stabilizes RORγt transcriptional activity and shapes Th17 pathogenicity in autoimmunity
Source: Cell Res. 2026 Jan 7;36(5):340–58. doi: 10.1038/s41422-025-01204-6 (PMC13092643; doi:10.1038/s41422-025-01204-6)
Supplement: Supplementary file 24 — Supplementary information, Table S11 [file 41422_2025_1204_MOESM24_ESM.pdf]

Table S11 – Deposited data

Deposited data name	Sourse	Identifier
ScRNA-seq data: Immune cell atlas from lymph nodes of naïve and EAU mice	This paper	GSA: CRA008977
ChIP-seq data: RORγt ChIP-Seq analysis of *in vitro* polarized WT (sgCtrl) or *PRKDC*^{KO} (sg*PRKDC*) or PQR-mutated (sg*PRKDC*$^{\Delta PQR}$) human Th17 cells	This paper	GSA: HRA006260
ChIP-seq data: RORγt ChIP-Seq analysis of *in vitro* polarized WT (sgCtrl) or *PRKDC*^{KO} (sg*PRKDC*) or PQR-mutated (sg*PRKDC*$^{\Delta PQR}$) human Th17 cells (Replicate of 2)	This paper	GSA: HRA012642
RNA-seq data: Human Th17 cells polarized by IL-6 + IL-1β or IL-6 + IL-1β + IL-23	This paper	GSA: HRA006243
ScRNA-seq data: Immune cell atlas of PBMCs from patients diagnosed with autoimmune uveitis and the paired healthy control donors	This paper	GSA: HRA001580
Single-cell TCR-sequencing (scTCR-seq) of IRBP-specific CD4+ T cells	This paper	GSA: CRA016165
ATAC-seq of human polarized pTh17 with PRKDC ablation	This paper	GSA: HRA013725
